# Improving Population-Level Maternal Health: A Hard Nut to Crack? Long Term Findings and Reflections on a 16-Community Randomised Trial in Australia to Improve Maternal Emotional and Physical Health after Birth [ISRCTN03464021]

**DOI:** 10.1371/journal.pone.0088457

**Published:** 2014-02-28

**Authors:** Rhonda Small, Lyndsey Watson, Jane Gunn, Creina Mitchell, Stephanie Brown

**Affiliations:** 1 Judith Lumley Centre, La Trobe University, Melbourne, Victoria, Australia; 2 Department of General Practice, University of Melbourne, Carlton, Victoria, Australia; 3 School of Nursing and Midwifery, Griffith Health Institute, Griffith University, Southport, Queensland, Australia; 4 Healthy Mothers Healthy Families Group, Murdoch Childrens Research Institute, Parkville, Victoria, Australia; University of Pennsylvania, United States of America

## Abstract

**Background:**

Community level interventions to improve maternal and child health have been supported and well evaluated in resource poor settings, but less so in developed countries. PRISM - Program of Resources, Information and Support for Mothers - was a primary care and community-based cluster-randomised trial in sixteen municipalities in Victoria, Australia, which aimed to reduce depression in mothers and improve their physical health.

The aim of this paper is to report the longer term outcomes of PRISM and to reflect on lessons learned from this universal community intervention to improve maternal health.

**Methods:**

Maternal health outcome data in PRISM were collected by postal questionnaire at six months and two years. At two years, the main outcome measures included the Edinburgh Postnatal Depression Scale (EPDS) and the SF-36. Secondary outcome measures included the Experience of Motherhood Scale (EOM) and the Parenting Stress Index (PSI). A primary intention to treat analysis was conducted, adjusting for the randomisation by cluster.

**Results:**

7,169/18,424 (39%) women responded to the postal questionnaire at two years −3,894 (40%) in the intervention arm and 3,275 (38%) in the comparison arm. Respondents were mostly representative on available population data comparisons. There were no differences in depression prevalence (EPDS≥13) between the intervention and comparison arms (13.4% vs 13.1%; ORadj = 1.06, 95%CI 0.91–1.24). Nor did women's mental health (MCS: 48.6 vs 49.1) or physical health scores (PCS: 49.1 vs 49.0) on the SF-36 differ between the trial arms.

**Conclusion:**

Improvement in maternal mental and physical health outcomes at the population level in the early years after childbirth remains a largely unmet challenge. Despite the lack of effectiveness of PRISM intervention strategies, important lessons about systems change, sustained investment and contextual understanding of the workability of intervention strategies can be drawn from the experience of PRISM.

Trial Registration. Controlled-Trials.com ISRCTN03464021

## Introduction

Thirty years ago there was little recognition that maternal depression following the birth of a baby was an important public health issue. That has now changed. Studies conducted since the late 1980s have shown maternal depression to be common, affecting between 10 and 20% of women after birth in high income countries, with rates higher again in low and middle income countries [1]–[3]. Associated risk factors have been elucidated [2], [3] and the consequences not only for women, but also for their children, are known to be significant, especially if maternal depression is unresolved or recurring [4]. There is strong evidence supporting targeted postnatal counselling interventions for reducing maternal depression in women identified as depressed [5], [6]. Universal (i.e. non-selective) interventions have also been tested in routine postnatal and primary care settings [5], [7], but only one trial – the study by MacArthur and colleagues of redesigned postnatal care – has demonstrated improved maternal mental health outcomes [8].

There are a number of reasons further investigation of universal strategies is warranted. For a start, the population prevalence of maternal depression has shifted little in the last 20 years in Australia [9]–[11] despite improved knowledge about effective responses. The capacity to predict which women are most likely to develop depression during and after pregnancy also remains elusive, in spite of good evidence regarding a range of psychosocial and other factors that are associated with maternal depression – social isolation, stressful life events, a history of prior depression and/or anxiety, intimate partner abuse, less severe relationship problems, and physical health problems [9], [10], [12]–[14]. Studies seeking to screen pregnant and postnatal women on the basis of psychosocial risk factors have not been able to demonstrate improved maternal postpartum health outcomes [15]. The question of when and how to screen women to identify risk of depression and/or current symptoms of depression is also problematic. Most intervention studies focus on pregnancy and/or the early weeks after childbirth [6]. Our combined work demonstrates that a majority of first time mothers who develop postnatal depressive symptoms experience the onset of symptoms later than three months postpartum, and that community prevalence of depressive symptoms in recent mothers remains high up to two years after having a baby [14], [16]. Thus intervention strategies focusing on pregnancy and/or the first three months postpartum are likely to miss a majority of cases.

The WHO Millennium Development Goals identify the importance of integrated primary health care as a cornerstone for improved maternal, newborn and child health outcomes. Community-based multi-component intervention strategies involving integration across sectors including primary health care, and implemented at a district or regional level - have been shown to work in a range of low and middle income settings [17], but universal approaches to improve maternal health have been less extensively implemented and evaluated in developed countries. Evaluation of community-based, multi-component, and by definition complex intervention strategies, present manifold challenges for diverse stakeholders. These challenges span everything from how to secure policy and operational support at local, regional and potentially national levels; the need to secure long-term funding for implementation and concurrent evaluation; and complex questions regarding ways to maximise sustainability, when it may be many months or years before outcomes of the intervention strategies are known.

PRISM was a primary care and community-based cluster-randomised trial conducted between 1999 and 2003 in sixteen municipalities in Victoria, Australia [18]–[22]. The aim was to reduce depression in recent mothers and to improve their physical health. PRISM implemented a range of strategies in intervention communities, including: maternal health and communication skills training for primary care providers (maternal and child health nurses and general practitioners) and provision of information resources for women, alongside community development activities and befriending opportunities to promote local support for mothers and reduce the isolation so commonly experienced by women with very young children.

From the outset, longer-term follow-up at two years postpartum was also planned. We aimed to explore two hypotheses: 1) that any reduction in intervention communities in maternal depression at six months would be sustained at two years postpartum; and 2) that, in the absence of the hypothesised reduction at six months, a reduction in maternal depression would be detected at two years. The rationale for investigating this second hypothesis was the very real possibility that strategies of greater support and more responsive primary health care services for recent mothers might take some time to effect change at the population level. Moreover, improvements for individual women might also be achieved in the longer term, but not be evident at six months postpartum.

Outcomes at six months were reported in 2006 and showed no differences between intervention and comparison communities in the prevalence of maternal depression at six months, or in women's overall mental or physical health status [21]. This paper reports the findings of two-year follow-up, investigating the second hypothesis above. We also reflect on the lessons learned in conducting PRISM.

## Methods

The protocol for this trial and supporting CONSORT checklist are available as supporting information; see Checklist S1 and Protocol S1. The cluster trial design [19] and the PRISM intervention program [18], [20]–[22] have been described in detail in previously published papers. Briefly, sixteen municipalities in the state of Victoria, Australia agreed to randomisation, with four metropolitan and four rural communities allocated to the intervention and to the comparison arms of the trial in a matched pair design.

It was not possible to undertake baseline data collection on maternal mental and physical health in each of the participating municipalities within the constraints of available funding awarded by the National Health and Medical Research Council. We had reliable prevalence data from our previous population-based statewide surveys in Victoria of between 14% and 17% [9], [10], but not reliable data for individual municipalities. This lack of prevalence data at municipal level was one reason for taking care in the process of pair-matching communities prior to randomization. To quote from our design paper:

“Pairing between communities was undertaken to minimize potential imbalance between the comparison and intervention arms of the study in the baseline level of primary outcomes and in associated risk factors, such as the size of each community, the size of the population of interest, and community capacity to implement the intervention.

Reliable maternal depression prevalence data by LGA were not available, so a range of data was obtained to assist with determining useful matching criteria, some of which were known to be associated with the risk of depression including income, maternal country of birth, and marital status.” [19, p 241]

PRISM strategies were embedded in the intervention communities over a 12 month period prior to outcome evaluation, and the strategies continued with program funding for a further twelve months. Outcome evaluation occurred via postal questionnaires sent six months and two years after birth to all mothers who had given birth in the participating communities over an eighteen month period (Feb 2000–August 2001). Mothers of infants who had died were excluded. Questionnaires were packaged with a covering letter and a stamped addressed envelope, grouped and mailed to municipalities where a name and address label was added from their maternal and child health program data system. Reminder cards were sent two and four weeks later. Questionnaires were returned direct to the research team to ensure anonymity and confidentiality.

The Ethics Committees of Monash University (1994: HEC No 78/94 – PRISM Ethics S1) and La Trobe University (1996: HEC No 96/62 – PRISM Ethics S2) approved the study, and all participating municipalities signed a Memorandum of Understanding agreeing to participation on behalf of their communities (available on the PRISM study website, at: http://www.latrobe.edu.au/__data/assets/pdf_file/0020/217028/MOU_bd.pdf). Ethics approvals included acceptance that women would not be individually invited to participate and that return of mailed questionnaires by women would be considered evidence of individual consent. PRISM is registered on the ISRCTN (Current Controlled Trials) Register with trial number: ISRCTN03464021, at: http://www.controlled-trials.com/ISRCTN03464021.

### Outcome measures at two years

The main outcome measures assessing women's health and well-being at two years mirrored those used at six months: the EPDS (a 10-item measure developed for use in the postnatal period, in which a score of ≥13 identifies probable depression) [23] and the physical and mental component scores (PCS and MCS) of the Short Form 36 (SF-36), a general health status measure [24]. The PCS and MCS were calculated using norms from the 1995 Australian National Health Survey [25] using appropriate female age-group sub-scale means adjusted for the specific female age range of the study group.

Secondary outcome measures – women's experiences of motherhood and overall parenting stress – were also assessed, using two standard measures, the Experience of Motherhood Questionnaire [26] and the Parenting Stress Index (Short Form) [27]. The former includes 20 items designed to assess how being a mother affects a woman's experiences of daily life, such as access to babysitting, social support, support with child care, enjoyment of sex, contact with friends, enjoyment of social life, confidence and fulfilment in being a mother, coping with stress and anxiety, and general satisfaction with life. The Parenting Stress Index (Short Form) includes 36 items designed to assess personal stress, stress related to interaction with the child and stresses resulting from the child's behavioural characteristics. A Total Stress score provides an indication of the overall level of parenting stress experienced.

### Sample size and power

Our original power calculations were done for our primary outcomes at six months with 80% power to determine a 3% difference in the proportion of women probably depressed on the EPDS and a difference of two in the SF-36 mental and physical health scores (MCS and PCS). For the two-year primary analyses we calculated that we had 73% power to determine a 3% difference in the proportion of women probably depressed on the EPDS and more than 90% power to determine a difference of two in the SF-36 mental and physical health scores (MCS and PCS), allowing for an inflation factor of two due to the cluster sampling, as previously determined [19].

### Analysis

The main trial outcomes at two years were analysed using logistic regression for binary response outcomes and linear regression for continuous variables, with adjustment for the randomisation by cluster using survey analysis procedure, and with pair matches broken to give greater efficiency with a small number of clusters [28]. Sub-group analyses undertaken to explore interaction effects found in the six month data between the intervention and pre-specified sub-groups were repeated for place of residence, maternal country of birth, family income and marital status.

All analyses were carried out using STATA [29].

### Data availability

Randomisation codes and data can be made available upon request.

## Results

### Respondent characteristics

**Figure 1:**
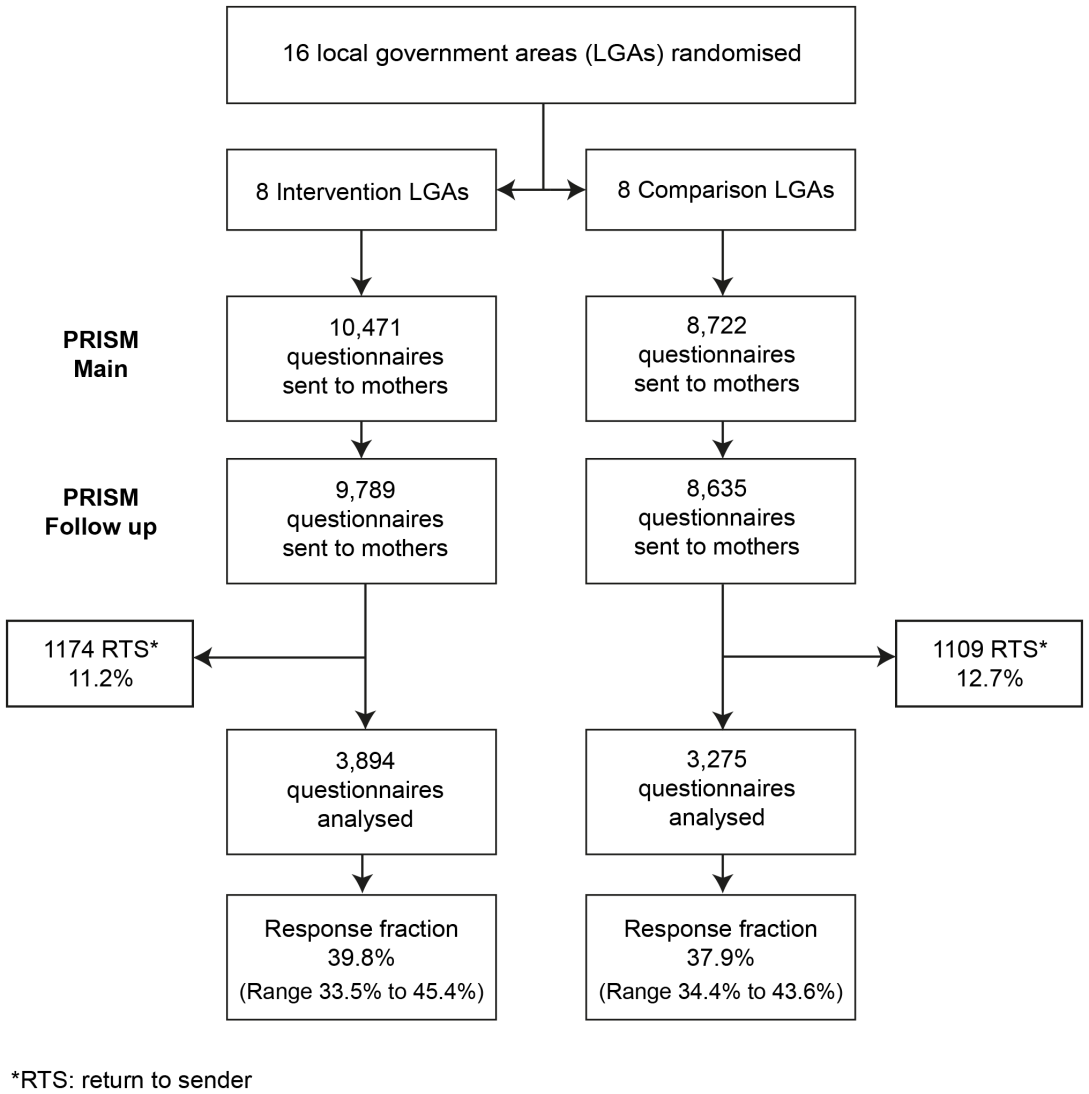
Trial flow diagram.

Figure 1 presents the trial flow diagram from randomisation of municipalities through to participant response at the two year follow-up. 7,169/18,424 (39%) women responded to the postal questionnaire at two years – 3,894 from intervention communities and 3,275 from comparison communities. The geometric mean for questionnaires mailed to women across all eight intervention communities was 1086, and the range was 346–1925. Across comparison communities the corresponding figures were 944, and 391–2049 . The geometric mean for participant responses in intervention communities was 428, and the range was 140–830. In comparison communities, the corresponding figures were 360, and 128–724. The overall response fractions were 39.8% in the intervention arm and 37.9% in the comparison arm, considerably lower than at the earlier six month follow-up (61.6% and 60.1%).

**Figure 2:**
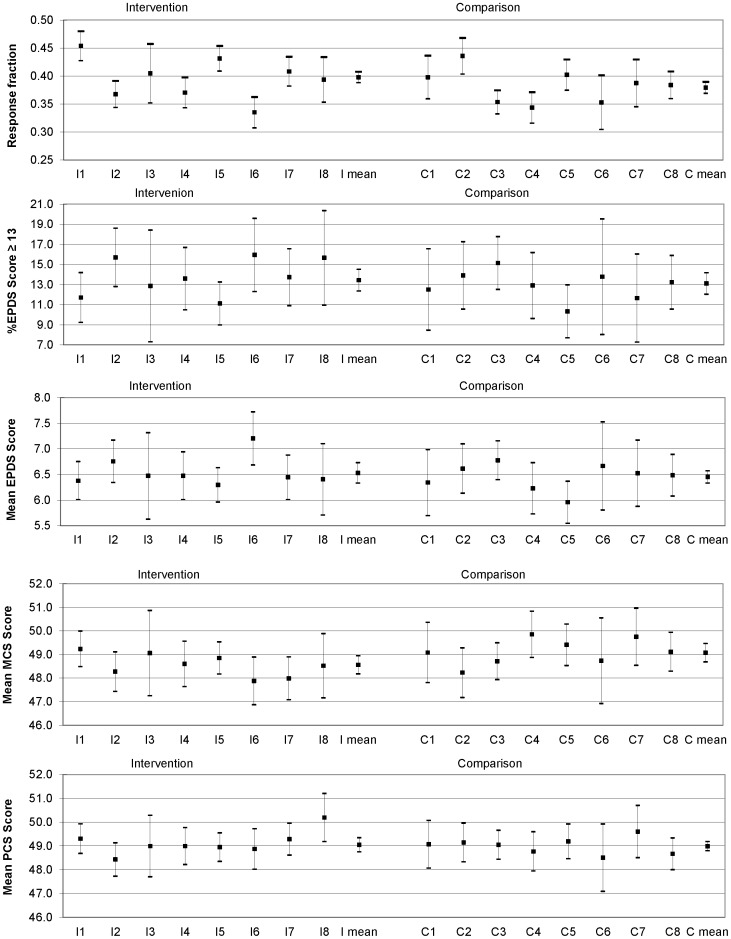
Response fractions and primary outcomes at two years in intervention and comparison communities.

The characteristics of women responding to the questionnaire sent two years after birth are shown in Table 1 for the intervention and comparison arms of the trial. Respondents differed little between the trial arms with regard to maternal age, education, marital status, family income, maternal country of birth and parity. The proportion of rural respondents was somewhat lower in the comparison arm than in the intervention arm (30.7% vs 37.3%). Figure 2 also displays response fractions for each intervention and comparison community.

**Table 1:** Characteristics of women responding at two years in intervention and comparison communities, compared with all women giving birth in PRISM communities.

	PRISM Follow-up				VPDC	
	Intervention		Comparison		Total	
	(n = 3894)	*%*	(n = 3275)	*%*	(n = 20333)	*%*
**Place of residence** *						
Metropolitan	2,444	*62.8*	2269	*69.3*	13,352	*65.7*
Rural	1,450	*37.2*	1006	*30.7*	6,981	*34.3*
**Maternal age in years** *						
≤19	54	*1.4*	33	*1.0*	734	*3.6*
20–24	326	*8.4*	209	*6.4*	2,593	*12.8*
25–29	1169	*30.0*	885	*27.0*	6,282	*30.9*
30–34	1490	*38.3*	1,381	*42.2*	6,966	*34.3*
≥35	852	*21.9*	760	*23.2*	3,757	*18.5*
Missing	3	*0.1*	7	*0.2*	1	*0.0*
**Highest education level attained** #						
Degree	1105	*28.4*	1053	*32.2*		
Diploma/Apprenticeship	1209	*31.0*	938	*28.6*		
Completed secondary school	664	*17.1*	536	*16.4*		
Did not complete secondary school	821	*21.1*	665	*20.3*		
Missing	95	*2.4*	83	*2.5*		
**Marital status** #						
Married	3213	*82.5*	2757	*84.2*		
Living with partner	403	*10.3*	325	*9.9*		
Single	138	*3.5*	88	*2.7*		
Separated/Divorced/Widowed	124	*3.2*	90	*2.7*		
Missing	16	*0.4*	15	*0.5*		
**Family income before tax in AUD per annum** #						
≤$30K	691	*17.7*	554	*16.9*		
30–70K	2023	*52.0*	1516	*46.3*		
>70K	902	*23.2*	953	*29.1*		
Missing	278	*7.1*	252	*7.7*		
**Country of birth**						
Australia	3446	*88.5*	2845	*86.9*	16,989	*83.6*
OSB: ES country	275	*7.1*	200	*6.1*		
OSB: NES country	165	*4.2*	223	*6.8*	3,318	*16.4*
Missing	8	*0.2*	7	*0.2*	26	*0.1*
**Parity** *						
Primiparous	1637	*42.0*	1,388	*42.4*	8,274	*40.7*
Multiparous	2257	*58.0*	1,887	*57.6*	12,059	*59.3*

* As at time of index birth.

# As at time of second survey.

AUD = Australian dollar.

OSB: ES country = Overseas-born English-speaking country.

OSB:NES country = Overseas-born non-English-speaking country.

VPDC = Victorian Perinatal Data Collection.

Table 1 further shows available data from the Victorian Perinatal Data Collection for all women who gave birth in participating communities during the PRISM study period, demonstrating that respondents to the two year questionnaire were largely representative with regard to place of residence, maternal country of birth and parity, but were less likely to have been under 25 years of age at the time of the PRISM index birth (8.6% vs 16.4%).

### Primary outcomes

Figure 2 shows data for the primary outcomes in each of the intervention and comparison communities: the proportion of women with EPDS scores ≥13, mean EPDS scores, mean mental health component scores (MCS) and mean physical health component scores (PCS) of the SF-36.

Table 2 summarises the differences in the main health outcome variables at two years after birth across intervention (I) and comparison (C) communities, adjusted for clustering. There is no evidence of differences on any outcome. The proportions of women with probable depression (EPDS≥13) two years after the index birth were 13.4% (I) and 13.1% (C), with an adjusted odds ratio of 1.06 (95%CI 0.91–1.24). The mean EPDS scores were 6.53 (SE_adj_ 0.10) and 6.45 (SE_adj_ 0.12).

**Table 2:** Probable depression (EPDS≥13 and mean scores) and SF-36 mental and physical component summary (MCS & PCS) scores and sub-scales, two years after birth.

	Intervention (n = 3894)				Comparison (n = 3275)				Statistical tests						
	n	%	mean	SE_adj_	n	%	mean	SE_adj_	p-value	OR_adj_	Diff_adj_	std err	95% CI		Inflation factor
**EPDS≥13**	3880	13.43			3266	13.10			0.92	1.01		0.08	0.85	1.20	1.26
**EPDS mean score**	3852		6.53	0.10	3235		6.45	0.12	0.61		0.03	0.14	−0.27	0.34	1.46
**SF-36**															
MCS*	3722		48.56	0.18	3111		49.08	0.18	0.06		−0.44	0.22	−0.92	0.03	1.00
PCS*	3722		49.05	0.14	3111		48.99	0.09	0.71		0.05	0.14	−0.24	0.35	1.00
**8 sub-scales**															
Physical Functioning	3864		88.95	0.43	3234		88.15	0.40	0.11		0.78	0.46	−0.19	1.74	1.36
Role-Physical	3826		80.23	0.40	3203		81.10	0.53	0.14		−0.98	0.63	−2.32	0.36	1.00
Bodily Pain	3889		75.74	0.38	3264		76.09	0.32	0.40		−0.31	0.36	−1.08	0.46	1.00
General Health	3857		73.60	0.54	3245		74.29	0.54	0.20		−0.45	0.37	−1.24	0.33	1.00
Vitality	3882		51.10	0.44	3255		52.19	0.32	0.04		−0.96	0.42	−1.86	−0.06	1.00
Social Functioning	3886		83.23	0.38	3269		84.13	0.31	0.11		−0.78	0.46	−1.77	0.20	1.00
Role-Emotional	3791		84.39	0.38	3185		85.09	0.43	0.30		−0.58	0.54	−1.73	0.58	1.00
Mental Health	3882		73.27	0.27	3255		73.74	0.41	0.23		−0.44	0.35	−1.18	0.31	1.00
Health Transition	3887		2.70	0.01	3263		2.74	0.02	0.10		−0.04	0.02	−0.01	0.08	1.05

*Scales adjusted for age/sex distribution of PRISM population, factor loadings and standard deviation using Australian National Health Survey values.

ABS. National Health Survey. SF-36 Population Norms Australia: Australian Bureau Statistics, Commonwealth of Australia Catalogue No. 4399.0; 1997.

In response to a separate question about feeling sad, blue or depressed for two weeks or more in the last two years, 25.4% of women in intervention communities reported that they had felt this way, compared with 26.6% of women in comparison communities. Very similar proportions of women also reported that they had no-one to talk to about how they were feeling: 5.9% (I) and 6.4% (C).

Overall physical and mental health status of women in both arms of the trial was similar. The adjusted mean PCS scores on the SF-36 were 49.1 (SE_adj_ 0.14) (I) and 49.0 (SE_adj_ 0.09) (C) and MCS scores were 48.6 (SE_adj_ 0.18) (I) and 49.1(SE_adj_ 0.18) (C). The SF-36 sub-scale scores are also shown in Table 2 and reveal no differences between the trial arms.

The pre-specified sub-group analyses repeated to investigate evidence of intervention interaction effects at six months among single women, women born overseas in non-English speaking countries and women on low incomes, found no evidence of such effects in favour of the intervention at two years (Table 3).

**Table 3:** EPDS and SF-36 mean scores two years after birth for selected pre-specified subgroups.

	Intervention				Comparison				Statistical tests					
	n	%	mean	SE_adj_	n	%	mean	SE_adj_	p-value	AOR	Diff_adj_	std err	95% CI	
**Single women**														
**EPDS≥13**	137	24.09			87	29.89			0.5	0.85			0.49	1.48
MCS*	131		45.41	1.03	86		44.93	1.59	0.6		0.96	1.76	−2.80	4.72
PCS*	131		48.47	0.72	86		47.61	0.99	0.5		0.93	1.29	−1.83	3.69
**Women born in non-English speaking countries**														
**EPDS≥13**	164	17.07			222	16.67			0.7	1.13			0.60	2.14
MCS*	151		49.51	1.04	199		48.68	0.39	0.3		0.92	0.91	−1.03	2.87
PCS*	151		47.59	0.42	199		47.75	0.57	0.4		−0.44	0.56	−1.64	0.75
**Income≤AUD20,000**														
**EPDS≥13**	334	24.55			257	26.85			0.7	0.89			0.52	1.53
MCS*	307		45.69	0.50	237		46.18	0.95	0.5		−0.67	1.06	−2.92	1.58
PCS*	307		47.86	0.35	237		47.98	0.42	0.7		−0.26	0.59	−1.51	0.99

*Scales adjusted for age/sex distribution of PRISM population, factor loadings and standard deviation using Australian National Health Survey values.

ABS. National Health Survey. SF-36 Population Norms Australia: Australian Bureau Statistics, Commonwealth of Australia Catalogue No. 4399.0; 1997.

### Secondary outcomes

Women's experiences of motherhood as measured by the mean scores on the Experience of Motherhood Questionnaire did not differ between intervention and comparison communities: 37.9 (I) and 37.8 (C), (t = −0.70, 95%CI −0.49 to 0.25). The proportions scoring over 40 on the scale indicating a more negative experience also did not differ: 34.5% (I) and 34.2% (C), OR_adj_ 1.01 (95%CI 0.90–1.13). Nor were there any differences evident in the proportion of women experiencing stress in their parenting role (raw score >85 on Total Stress Score on the Parenting Stress Index): 20.2% (I) and 21.7% (C), OR_adj_ 0.91 (95%CI 0.82–1.01).

Table 4 describes - for both intervention and comparison communities - women's reports of information received, their experiences with primary care services (GPs and MCHNs), their friendships in the two years since the birth, their views about the mother-baby friendliness of their communities, and their accounts of partner involvement with children, household division of labour and overall support received from their partners. There were no differences between intervention and comparison communities in women's reporting of any of these aspects of their lives two years after the birth, with the exception that, as expected, very few women in comparison communities reported having received the PRISM information kit after the birth of their two year old.

**Table 4:** Women's reports two years after birth about information received, primary care support, friendships, community support and partner support in intervention and comparison communities.

	Intervention		Comparison	
	N	%	N	%
**Information Kit**				
Received the kit shortly after birth of two year old *(photo of kit shown in questionnaire)*	3453	*88.8*	333	*10.2*
Still have it, or gave it to a friend *(% of those who received it)*	1769	*51.2*	74	*22.2*
**Activities for mothers**				
Participated in local activities for mothers in the last two years	1210	*31.4*	968	*29.8*
**Primary care support**				
MCHN very supportive and understanding *(% of those attending MCHN in the last 12 months)*	1559	*47.1*	1354	*48.3*
GP very supportive and understanding *(% of those attending MCHN in the last 12 months)*	2442	*68.5*	1954	*65.8*
**Friendships/‘time out’**				
Made new friends in the last two years	3306	*85.1*	2759	*84.4*
Had more social contacts in the local community than two years ago	1005	*25.9*	783	*24.0*
Has ‘time out’ from looking after children at least once a week	2085	*53.6*	1702	*52.0*
**Mother-baby friendliness of the local community**				
Very	667	*17.6*	540	*16.9*
Fairly	1608	*42.3*	1223	*38.3*
Mixed, or not mother-baby friendly	1522	*40.1*	1429	*44.8*
**Partner involvement with children** *(% of those married or living with a partner)*				
Coped well or fairly well with changes brought about by living with children	3439	*95.4*	2941	*95.8*
Spent at least four hours looking after the children in the last week	2024	*56.6*	1678	*55.4*
Happy with partner's involvement given work commitments	2990	*83.0*	2488	*80.9*
Agree with partner always or mostly about how to bring up children	3310	*91.8*	2796	*90.9*
**Partner involvement in household tasks**				
Share things evenly	1036	*28.8*	856	*27.9*
Mother does everything inside, partner outside	1114	*31.0*	932	*30.4*
Mother does everything outside, partner inside	5	*0.1*	6	*0.2*
Mother does most things inside and out	547	*15.2*	515	*16.8*
Partner does most things inside and out	42	*1.2*	53	*1.7*
Mother does most things due to partner's work	681	*19.0*	568	*18.5*
Other	168	*4.7*	136	*4.4*

	N	Mean	N	Mean
**Partner support score** (out of 8, four questions)	3616	4.78	3082	4.74
SE_adj_		0.05		0.04

[Difference: −0.034,SE_adj_: 0.06, p-value: 0.58].

## Discussion

At the population level it is clear PRISM did not demonstrate any improvements in the emotional or physical health of mothers two years after birth in intervention communities – just as it had not at six months postpartum. Response fractions for the two-year follow-up were lower than hoped, but it is unlikely this contributed to the finding of no effect, as respondents and non-respondents did not differ greatly in comparisons on available population data for the PRISM study period.

Why did PRISM fail to make a difference to women's health outcomes? One hypothesis is that it was too ambitious, especially given the time and the resources available to the trial. We explore this hypothesis in the following discussion of the trial's strengths and limitations and we sketch out some of the lessons to be learned from our experiences of conducting PRISM.

### Strengths, limitations and lessons

#### Overall design

PRISM had significant strengths, but also some important limitations. The trial was designed in the mid-1990s, on the very first wave of interest in the design and evaluation of complex interventions [30]. There were no examples of complex community interventions targeting depression for the investigator team to draw upon or learn from. Indeed, to this day, PRISM remains the only community randomised trial with depression as a primary outcome.

PRISM was a carefully designed and rigorously evaluated cluster randomised trial, powered appropriately to detect a small, but important effect. The intervention strategies were implemented over a two year period in eight municipalities spanning regional and metropolitan parts of Victoria, Australia. All clusters were retained to completion and PRISM involved detailed process and impact evaluation (and reported on the PRISM website blinded to trial outcomes), as well as an economic evaluation [31]. The intervention was characterised by clearly articulated key elements, with scope for local tailoring and the addition of locally initiated strategies [21], [22]. All these are aspects emphasised as important in recent literature about the design and reporting of complex interventions [32]. That PRISM failed to demonstrate a difference to the health of mothers in the intervention communities was probably not as a result of poor overall trial design.

#### Was a universal approach the right one?

PRISM was designed to address issues identified in descriptive epidemiological research. A series of studies undertaken by four members of the investigator team in the 1990s had shown that 95% of mothers experience health problems after childbirth; that one in six experience depression in the first year after childbirth; that lack of social support, social isolation and poor physical health are contributing factors to maternal depression; and that many women are reluctant to disclose health problems to primary care practitioners despite having considerable contact in the first six months after childbirth [10], [33]–[36].

Our rationale for adopting a universal approach, focusing on both physical and mental health, and engaging both primary care and community-based agencies in the implementation of intervention strategies was premised on several key assumptions. The first was that for the intervention strategy to be effective in encouraging participation and disclosure of health problems, it had to be non-stigmatising for mothers. The second was that it needed to be multi-faceted, community-wide and underpinned by inter-sectoral collaboration, with scope for tailoring of specific intervention strategies to local community contexts. By focusing on *all* mothers and not just those already experiencing depression, or ‘at risk’ of depression, PRISM strategies aimed to provide all mothers with more supportive local communities, thus making support for women with young children normative, rather than a sign of ‘failure’. In order to reach diverse groups of women and affect a range of aspects of women's lives – a whole of community approach, incorporating inter-sectoral strategies, with tailoring to local contexts was seen as essential to the likely success of intervention strategies [18].

#### Inadequate uptake of PRISM strategies: Was it clear whose job it was to do what?

In practice, achieving broad-based community level engagement was an enormous challenge and very difficult to sustain. Adoption of PRISM strategies by primary care practitioners and communities was variable. Possible reasons for this are easy to identify, especially in retrospect. While all local government authorities taking part in the study had expressed interest in participating and signed a memorandum of understanding regarding what this would entail, the changeover of councils and staff during PRISM meant that it was necessary to keep re-establishing commitment throughout the intervention period and afterwards. When PRISM commenced, local government in Victoria had not long been re-organised – entailing a reduction in the number of municipalities from over 200 to 78 – and all municipalities were struggling with the logistics of larger geographic boundaries, amalgamation of services and the introduction of compulsory competitive tendering [37].

It became apparent over time that not all maternal and child health teams had been sufficiently consulted or involved in making decisions about participation in the trial. Some teams and some individuals within teams embraced the intervention strategies, but others were resistant to them and saw implementation of PRISM as principally the Community Development Officer's responsibility [38]. For General Practice Divisions the fact that the PRISM intervention communities were based on local government areas, which have different geographic boundaries to General Practice Divisions was a barrier to engagement. An even more important barrier was the lack of effective systems for integrated service delivery involving general practitioners and maternal and child health nurses. It was an achievement of PRISM that Local Steering Committees (a key element of the intervention strategy) brought these diverse stakeholders together on a regular basis for the two year period of the trial. With the benefit of hindsight however, it is perhaps not surprising that the diverse actors involved found it difficult to achieve change on a sufficient scale in the available time to affect the primary outcomes measured in the trial.

#### Did PRISM lack reach? Were PRISM resources spread too thin?

There is no doubt that PRISM touched the lives of many people. Local Steering Committees did meet regularly, all maternal and child health teams and 15.4% of eligible general practitioners in intervention communities (range 10–70%) participated in training [39]. PRISM information kits were distributed to mothers, and a range of PRISM events and activities happened in all communities [21]. The level of commitment of the participating local government areas to supporting the evaluation process was outstanding; questionnaires and reminder postcards were mailed out every two weeks in all 16 municipalities over a three-year period. We acknowledge however, that the considerable variability in response fractions for the two year follow-up, as seen in Figure 1, may indicate distribution fatigue in some communities towards the end of data collection.

Diverse stakeholders did get involved in PRISM, including: general practitioners and maternal and child health nurse teams in each municipality; General Practice Divisions; elected local government officials and government appointed administrators; community services managers employed by local government; a multiplicity of community based agencies and community groups (eg libraries, neighbourhood houses, welfare agencies, church groups, and sports clubs); and an equally varied range of local businesses (eg shops, cafes, leisure centres) in each intervention community. Process evaluation conducted during the trial provided evidence that the key elements of PRISM ‘happened’, but was less effective in telling us about their reach. While outcome evaluation at six months showed that most women in intervention communities did receive PRISM information kits from their maternal and child health nurses, it appeared that other strategies (such as improving the responsiveness of all primary care providers to maternal health issues and enhancing opportunities for women to develop supportive local friendships post birth) did not achieve adequate potency or reach across the whole population of women giving birth. Without a means to identify how women were responding to the strategies prior to outcome evaluation, there was no opportunity to modify or improve them during the trial. Again with hindsight, more time and greater resources were probably needed to ensure all key elements were functioning optimally before outcome evaluation occurred.

Indeed adequate funding was a major issue for the trial. In order to resource key elements of PRISM, we continued throughout the implementation period to submit applications to a range of government and philanthropic funding bodies, eventually securing more than $964,749AUD to support implementation, alongside $1,152,793 for evaluation, including three grants from the National Health and Medical Research Council (Australia). This continual need for the lead researchers to spend time preparing funding applications, and the degree of uncertainty this generated for participating municipalities, undoubtedly had impacts on how the intervention unfolded and on our capacity to embed formative and iterative research strategies in the research design [40].

#### Would a targeted approach have been more effective?

PRISM represented a significant investment of time and effort. Would the limited resources available for implementation of the trial have been better targeted to those who are most vulnerable, for example women who are socially disadvantaged or socially isolated, such as young women or single mothers? At the time, we deliberately chose not to do this – both because other studies had done so [41], [42] and because of the findings of our prior research showing that single women and those experiencing economic hardship make up only a small proportion of women experiencing depression and physical health problems after childbirth [9], [34]. While it is possible to see the fact that PRISM failed to achieve an improvement in health outcomes as a failure of universalism, this would be missing the point of what the study set out to do and why it was designed in that way.

Nonetheless, diversity was an issue for PRISM intervention communities. It was difficult for local steering committees and other PRISM stakeholders to tailor PRISM strategies to meet the needs of diverse population groups, such as immigrants and refugees, Indigenous women and younger women. This was partly a matter of resources and a relatively short time frame for implementation of PRISM strategies, but also undoubtedly reflected priorities of local stakeholders and agencies represented on steering committees in each intervention community.

#### A lack of theory? Were key elements of the PRISM intervention program insufficiently conceptualised?

**Figure 3:**
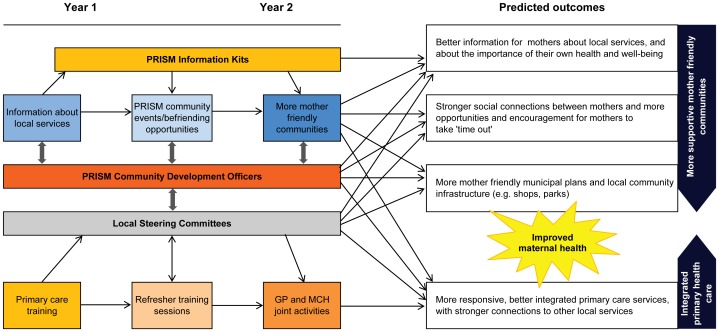
Schematic overview of conceptual thinking behind PRISM.

Fundamentally, PRISM was about change – changing communities to be more mother and baby friendly; changing the way that primary care practitioners provide support to mothers and moving towards more integrated systems of primary care; changing the way that mothers think about their own health and well-being and the actions they take to look after their own health, as well as that of their children and other family members. Figure 3 provides a schematic overview of our conceptual thinking about the intervention when we designed PRISM. The intervention strategies to achieve change were not underpinned by a particular conceptual theory or set of theories, and the study has been criticised for this [43]. Had we invested time in framing the intervention strategies according to a particular conceptual theory and then using this as the basis of process evaluation, we might have chosen to be less ambitious in the number of key elements implemented, and the number of stakeholders and agencies involved. Yet, the need to act at multiple levels was clear from our previous research and the key PRISM elements in fact combined individual strategies tried elsewhere, with at least some evidence of benefit [22]. Whether or not more conceptual framing of PRISM strategies would have enhanced the chance of PRISM being effective remains unclear, but a deeper understanding of the challenges of implementing change in complex systems, at the start of PRISM, would undoubtedly have led us to plan for significantly more than a 12 month ‘embedding’ phase to achieve the desired changes in primary care practice and community activity to support mothers.

#### Are other strategies the answer?

In the decade preceding PRISM and the decade since, millions of public health dollars have been spent on a range of initiatives: public awareness campaigns focusing on maternal depression, professional training in perinatal mental health care for general practitioners and maternal and child health nurses, partnership programs promoting greater integration across primary care and perinatal depression screening for women. None has involved concurrent evaluation of outcomes for women, and as noted earlier, the population prevalence of maternal depression in Australia has shifted little in the last 20 years [9], [10], [12].

In 2009 the Australian government began rolling out funding to support universal screening both in pregnancy and in the first few months after childbirth, despite lack of evidence yet that screening results in better population-level perinatal mental health. Implementation of this universal screening has been far from smooth [44] and its impacts are as yet unknown.

What *is* clear is that mothers continue to experience a high level of psychological and physical morbidity across the perinatal period, and current service models and community level programs are failing to provide adequate support to thousands of women every year. Thirteen per cent of women who returned PRISM questionnaires at two years postpartum had scores on the EPDS suggesting that they were probably clinically depressed. The true prevalence is likely to be higher as women vulnerable to depression – women experiencing intimate partner violence, young women, and women on low incomes – were also more likely to have moved and been lost to follow up at two years.

#### Key lessons from PRISM

While PRISM strategies failed to change primary care responsiveness to maternal health issues or demonstrate improved community support for mothers, and therefore, not surprisingly, PRISM failed to improve maternal health outcomes, we *can* and *should* learn from PRISM to improve future intervention strategies.

As noted already, some of the issues tackled in PRISM have longstanding ‘histories’ in Australia. In particular the lack of integration of maternal and child health services with general practice has roots in the different funding and payment systems supporting these services. The challenges of bringing about more integrated systems of maternal and child health care, and of working with diverse stakeholders across eight municipalities, each managing the transition to a new funding model, and each with a different set of local organisational issues, were undoubtedly under-estimated in PRISM. In addition, our ambitions for mobilising support for, and improving the health of mothers almost certainly required more time and more resources than we had been able to marshal, in order to build and sustain the truly collaborative partnerships necessary for implementing change at community level. While PRISM did include a range of process and impact evaluation strategies [21], [22], ultimately we had too little capacity to work with communities to modify or enhance implementation strategies sufficiently, ahead of outcome evaluation. Incorporating sufficient time in complex intervention trials for the collaborative development, implementation, assessment and modification of interventions as they become embedded in practice, is clearly an important lesson to be drawn from our experiences in PRISM.

### Next steps

PRISM demonstrated that there remains significant room for improvement in primary care responsiveness to women in the postpartum period in Australia. In both arms of the trial only around half of the women found their maternal and child health nurses very supportive. A slightly higher proportion found their general practitioners to be very supportive, but one in three women rated this care as less than optimal. As maternal and child health nurses and general practitioners are the key providers of postpartum care to women, these ratings must be a concern. Women's perceptions of the mother-baby friendliness of their local communities was also quite low, with fewer than 20% rating their communities as very mother-baby friendly, suggesting that here too, more needs to be done. The key to achieving the better outcomes PRISM sought for mothers may lie in strengthening the efforts made to improve the responsiveness of postpartum care and to enhance community support by more sustainably engaging the key stakeholders in both these sectors in the development, implementation and appraisal of change strategies.

PRISM had no capacity to effect change at a national policy or regulatory level. Our attempts to mobilise community support for mothers were aimed at the local community and at local government. But of course women's lives are affected by policy and legislative contexts far beyond their local communities. Are there national initiatives that would make a difference to maternal health outcomes? For the first time, the Australian Government introduced a universal parental leave scheme in 2011, offering employed women six months leave after childbirth, paid at the minimum wage. In 2013, two weeks of ‘Dad and Partner Pay’ was added to the scheme [45]. Although the effects of such measures on maternal health at the population level will remain unknown for some time yet, it is of interest that prior to the introduction of the national scheme, women participating in a pregnancy cohort study who had access to employer-supported parental leave were less likely to report intimate partner violence in the first year after birth than those who had no such access, even after controlling for education and income [46].

Increasing the availability of affordable childcare and flexible working hours for men and women with young children are also potentially relevant policy interventions to improve maternal (and paternal) health. Would, for example, the introduction of shorter working hours for both men and women while children are young make a difference, providing a symbol that the community values childrearing and encouraging more shared parental responsibility in caring for children, as well as reducing isolation for mothers? Forty-five per cent of partners provided less than four hours of child caring in the last week according to the women who responded to the two-year follow-up questionnaire in PRISM. Shorter working hours for parents of young children are legislated in Sweden, a country where the national prevalence of maternal depression is around 11% [47]. This is indeed somewhat lower than the 14% to 17% found over many years in population-based studies in Australia [9], [10], [12].

Fathers were not a major focus of PRISM strategies, although a leaflet with tips for fathers on supporting their partners after birth *was* included in the information kit provided to mothers in intervention areas. Should there be more focus on fathers/partners and their role in maternal wellbeing after childbirth? Lack of partner support remains an important contributing factor in maternal depression, yet universal, couple-focused interventions are rare. A recent exception is a non-randomised, but controlled study involving a nurse-facilitated, brief psycho-educational program for mothers, fathers and first newborns which focused on infant behaviour management and adjustment in the intimate partner relationship, with evidence of apparent benefit for maternal mental health outcomes [48].

### Conclusions

We remain of the view that change is needed, both at systems and community level to improve maternal health outcomes. The difficulties encountered by the municipalities that implemented PRISM intervention strategies are not specific to PRISM, they reflect challenges associated with implementing change in any setting. Our hope is that others will not be dissuaded from implementing and evaluating large scale intersectoral projects by the failure of PRISM to achieve changes leading to improved health outcomes. However, our findings do tell a cautionary tale about the need for high level and sustained engagement with key stakeholders in all stages of implementation and evaluation, and for researchers and funders to recognise the long-term commitment, substantial investment and collaborative efforts required to effect change. Despite much having been written about complex interventions since the PRISM trial ended [49]–[55], there remain enormous challenges for implementation science to develop the conceptual and practical tools to aid understanding of what makes interventions ‘workable’ in complex systems.

Finally, investment in ‘a healthy start to life’ is now widely recognised as the most effective strategy for reducing health inequalities across the life course [56]–[58] and there has been a recent call for integrating strategies to improve maternal mental health in universal maternal and child health care systems [59]. More than anything, such recognition reinforces the importance of finding effective strategies to promote maternal health as the cornerstone of child and adult health outcomes.

## Supporting Information

Checklist S1 **Completed CONSORT checklist for cluster trials.**
(DOCX)

PRISM Ethics S1 **Trial ethics approval from Monash University.**
(PDF)

PRISM Ethics S2 **Trial ethics approval from La Trobe University.**
(PDF)

Protocol S1 **PRISM protocol, BMC Public Health 2003.**
(PDF)
