# Supplementary material for: Improving Population-Level Maternal Health: A Hard Nut to Crack? Long Term Findings and Reflections on a 16-Community Randomised Trial in Australia to Improve Maternal Emotional and Physical Health after Birth [ISRCTN03464021]
Source: PLoS One. 2014 Feb 28;9(2):e88457. doi: 10.1371/journal.pone.0088457 (PMC3938427; doi:10.1371/journal.pone.0088457)
Supplement: PRISM Ethics S1 — Trial ethics approval from Monash University. (PDF) [file pone.0088457.s002.pdf]

M O N A S H   U N I V E R S I T Y

AUSTRALIA

MEMO

20 June 1994

Ms R Small
Centre for the Study of Mother's
and Children's Health
463 Cardigan Street
CARLTON 3053

Re: Project 78/94: Strategies for reducing depression after birth.

The Standing Committee on Ethics in Research on Humans has approved the above project and advice confirming clearance for this project has been forwarded to the NH&MRC.

A copy of the NH&MRC form is enclosed for your records.

Combined approval for two trials:

1. Community Intervention Trial (PRISM subsequently)
2. Debriefing ~~after~~ assisted delivery Trial

Christine Edwards

Ms Christine Edwards
Acting Secretary
The Standing Committee on Ethics in Research on Humans

Copy: Dr J Lumley, as above.

Encl.

CE:ER (SCERH)A:\MEETING.594 File 94\0734

76/94

18

NHMRC Project Grants
INSTITUTIONAL APPROVAL FORM FOR
RESEARCH INVOLVING HUMANS

One (1) copy of the completed approval form should be either attached to the original application or sent separately to the Secretary of Council no later than 31 July.

APPLICANT USE — Please complete in BLACK type or ink only

Chief Investigator

	Surname	Title	Initials
A	LUMLEY	DR	JM
B	SMALL	MS	RE
C	WATSON	MS	LW
D			

Scientific Project Title:

REDUCING DEPRESSION AFTER BIRTH: TRIALLING COMMUNITY STRATEGIES

Administering Institution

MONASH UNIVERSITY

ETHICS COMMITTEE USE

Does this Project comply with the provisions contained in the Council's document 'Statement on Human Experimentation and Supplementary Notes'? Y/N ☐

Does this Project comply with the regulations governing experimentation on humans within your Institution and within your State or Territory? Y/N ☐

Comments, provisos or reservations:

Name of responsible Ethics Committee

The Standing Committee on Ethics in Research on Humans

Name of Ethics Committee representative (Block letters):

Surname	Title	Initials
ADAMSON	ASSOC PROFESSOR	T M

Signature: [signature] Date: 14.6.94

Note: (1) This form has been produced in an effort to standardise and effectively record ethics approval for all projects submitted to the NHMRC. Should it prove inappropriate, an individual statement may be forwarded in lieu. As the Council cannot recommend support if ethics clearance is not provided, it is of utmost importance that this information is received.

(2) If there is no appropriate Ethics Committee at the institution concerned, the Head of Department, or, in the case of individual researchers, the applicants themselves, should ensure that the proposal is submitted to an established Ethics Committee at a hospital or university for consideration prior to completing and signing the rest of the form as an undertaking that the provisions of the NHMRC 'Statement on Human Experimentation and Supplementary Notes' will be observed.

AUSTRALIA

MEMO

17 June 1994

Ms R Small
Centre for the Study of Mother's
and Children's Health
463 Cardigan Street
CARLTON 3053

Re: Project 78/94: Strategies for reducing depression after birth.

The Standing Committee on Ethics in Research on Humans considered your submission at its meeting held on Tuesday 14 June 1994. The Committee agreed to approve the project as conforming to NH&MRC Guidelines. This approval is of the project as submitted and if any changes are subsequently made, the Committee should be advised. Please quote the project number above in any further correspondence.

Institutional Ethics Committees are requested by the NH&MRC to monitor research projects until completion to ensure that they continue to conform with approved ethical standards. The Committee undertakes this role by means of annual progress reports and termination reports. Please endure that the Committee is provided with a brief summary of the outcomes of your project when the project has concluded.

The Chief Investigators of approved projects are responsible for the storage and retention of original data pertaining to a project, for a minimum period of five years. You are requested to comply with this requirement.

Christine Edwards

Ms Christine Edwards
Acting Secretary
The Standing Committee on Ethics in Research on Humans

Copy: Dr J Lumley, as addressee.

CE:ER (SCERH)A:\MEETING.594 File 94\0738

M O N A S H   U N I V E R S I T Y

STANDING COMMITTEE ON ETHICS IN RESEARCH ON HUMANS

APPLICATION FOR ETHICAL APPROVAL OF A PROJECT USING HUMANS

Applications may be typewritten on this form or word processed on plain paper following the same format but ensuring that this page is retained, and should be forwarded to:-
The Secretary
Standing Committee on Ethics in Research on Humans
University Offices, Clayton

Project Number
(For Office Use Only)

Please refer to the Explanatory Notes attached when completing this form.

Short Project Title used on grant submission (see Note 1)
STRATEGIES FOR REDUCING DEPRESSION AFTER BIRTH

Name(s), Title(s), Qualifications and Department/Location of Chief, Associate and Co-Investigators (see Note 2) Where a student is involved, make clear who is the student and who is the supervisor.

CHIEF INVESTIGATORS: DR JUDITH LUMLEY (MB BS, PhD, FAFPHM), MS RHONDA SMALL (BA, DipEd, Grad Dip Lib), MS LYNDSEY WATSON (BSc, MSc) } CENTRE FOR THE STUDY OF MOTHERS' & CHILDREN'S HEALTH

ASSOC. INVESTIGATORS: MS STEPHANIE BROWN (BA Hons) } CENTRE FOR THE STUDY OF MOTHERS' & CHILDREN'S HEALTH
DR HILARY SCHOFIELD (BA, MEd, PhD) - Psychiatry, University of Melb.

Contact Person (See Note 3) RHONDA SMALL

Dept/Location
CENTRE FOR THE STUDY OF
MOTHERS' & CHILDREN'S HEALTH
463 CARDIGAN ST
CARLTON 3053

Contact Telephone Number 348 1047

Contact Facsimile Number 348 1129

Have you applied for external funding? (Yes) /No

If yes, indicate granting bodies
NH + MRC Public Health Research + Development Committee

Has funding been approved? Yes/ No (Pending)

Project Type - please mark appropriate boxes

(Research)   Teaching   Other

Student Research   Name of Degree..

If applicable please give previous Monash University project number: ...../.....

Proposed Commencement Date 1./1./95. **Proposed Duration of Project** .... Months/Years
Debriefing Trial: 4¼ year
Community Trial: 6y

Has this project been submitted to any other Ethics Committee? (see Note 4) Yes (No)

If Yes, to which committee?

Has approval been granted?

Yes / No / Pending

Attach copy of the approval if available

M&C
Centre for the Study of
Mothers' and Children's Health

463 Cardigan Street
Carlton Victoria 3053
Australia

Director Dr Judith Lumley

Telephone (03) 348 1211
(03) 347 0797
Facsimile (03) 348 1129

29 May 1994

Ms Christine Edwards
Secretary
Standing Committee on Ethics
in Research on Humans
Monash University
Clayton 3168

Dear Ms Edwards

Please find enclosed an application for ethical approval which we hope can go before the Standing Committee at its meeting on 14 June 1994.

The application relates to a program of research submitted earlier this year to the NH&MRC for funding from the Public Health Research and Development Committee. We are applying for ethical approval for two of the projects within this program:

i) Reducing depression after operative delivery: a trial of debriefing, and
ii) Reducing depression after birth: a trial of community strategies.

I hope that you will find all the necessary information attached. Please contact me should you wish me to provide anything further.

Yours sincerely

Rhonda Small

Rhonda Small
for the Research Team

SECTION A - GENERAL INFORMATION

1. Give a succinct but comprehensive statement of the aims, hypotheses and potential significance of the project
2. Give a succinct but comprehensive statement of the academic background to the project and project plan

Context:

Maternal depression in the year after birth is a major public health problem. The point prevalence was 15.4% [95%CI 12.8-18.0] using a well-standardised measure[3-5] 8 to 9 months after birth in a Victorian population survey[1], with similar rates using the same measure in NSW[2] and (using a different measure) in a prospective, hospital-based, Victorian study[6]. It may be prolonged: 2 years after birth 30% of the women who had been depressed were still depressed or depressed again[25]. Only one-third of the depressed women sought help from a health professional (though all were in contact with a GP and a Maternal and Child Health Nurse [MCHN]) and fewer than 10% sought help from a mental health professional[25]. The effects of maternal depression are not limited to women themselves but include adverse effects on child health and development[7]. Reducing the severity and duration of depression after birth is a nominated national health goal[8].

An association of depression with assisted delivery has been increasingly reported in cross-sectional studies[1,11,13] and in 2 recent prospective ones[14-15]. Associations with reduced availability of social support and negative life events have been consistently found[9] with more recent evidence linking depression also to physical ill-health and to bottle-feeding[1,13,29]

(Reference numbering here refers to the Literature Review contained in the attached copy of the grant application for this research.)

Objectives:

1 To reduce the prevalence of depression (score >12 on the Edinburgh Postnatal Depression Scale) six months after assisted birth (forceps or caesarean) from 24% to 16%.

2 To reduce the community prevalence of depression 6-9 months after birth from 15.5% to 12.5%, and to reduce the prevalence of depression 18-21 months after birth in women who were depressed at 6-9 months from 30% to 20%.

3 To reduce maternal morbidity at 3 and 6 months postpartum (assessed with the SF-36[52]) and to increase breastfeeding at 3 months from 35% to 50%

Methods:

The objectives are to be achieved by three different interventions each of them carried out within a randomised trial. <u>The first intervention</u> is a trial of 'debriefing' about the labour, birth and post-delivery events, offered after assisted delivery, a day or so before discharge by an experienced midwife/MCHN. Secondary aims are the reduction of dissatisfaction with maternity care and an increase in subsequent vaginal birth. <u>The second intervention</u> is a trial of community-based strategies to increase recognition of depression in all primary care contacts, to increase listening skills and explicit offers of 'time to talk' by GPs and MCHNs, to improve the recognition and treatment of common postnatal physical health problems in primary care; to increase the availability of support and 'time out' for recent mothers through a review of the availability and accessibility of relevant services, an information kit for new mothers and the establishment of a mother-to-mother support network based on the principle of non-professional befriending. Randomisation would be of 14 communities, within pairs, with pairs stratified on important covariates. <u>The third intervention</u> is a trial of an early postnatal appointment with a GP, a week after hospital discharge, to prevent some of the common physical and emotional problems of the first weeks and months and to increase breastfeeding.

(**NB.** Ethics approval is not being sought From Monash University Research and Ethics Committee for this third trial. Applications for ethical approval have been submitted to the hospitals where recruitment will take place and the results of these applications are pending. The rest of this application refers only to the first two trials.)

Benefits:

If successful there will be immediate benefits for the women involved with flow-on effects to children and families. In addition, both interventions are characterised by relative simplicity which would permit ready implementation within standard models of care for mothers and babies. The debriefing trial involves the development of a manual for use by midwives in implementing the intervention as a regular component of postnatal care. The community intervention trial provides relevant skills enhancement for primary care providers also of benefit to other clients, and develops a model for joint action on a significant public health issue between the primary care sector, local government and community agencies, a strategy which if successful has implications for other mental health and human service programs.

It is acknowledged that the generalisability of the findings will be limited largely to women of English speaking backgrounds given that the instruments used to measure outcomes will require a capacity to read and write in English.

The Centre is currently undertaking a study involving Vietnamese, Turkish and Filipino mothers which is exploring their experiences of maternity care and life with a baby, including their experiences of depression in the postnatal period and we are in communication with Prof Bryanne Barnett in Sydney regarding her study validating the use of the Edinburgh Postnatal Depression Scale in translation with various ethnic communities. It is hoped that the findings of these studies will provide sufficient information about the emotional well-being of mothers of non-English speaking backgrounds to ascertain whether the strategies to be trialled in the Program are also likely to be appropriate in these groups.

For further details of the Research Plan, please refer to the attached grant application.

3. Is there any alternative to using human, e.g. prior computer modelling, laboratory or animal experiments?

Yes / No (No circled)

4. Briefly describe all procedures to be used with humans

Debriefing trial:

The intervention in this trial is the provision of 'debriefing' about the labour, birth and post-delivery events and experiences. It will be offered a day or so before discharge and will be carried out by an experienced midwife/MCHN. The content of the discussion will be the experiences of labour and birth, any gaps in the woman's recollections (eg due to anaesthesia), any unanswered questions about the reasons for the operative delivery, what is in the medical record, choices and decision-making, her partner's concerns, physical health and recovery, implications for future births. (Not all these issues will be relevant to every woman). A manual for the debriefing will be developed and piloted by the research team in the first three months of the project. Women in the control group will receive a brief visit from the midwife to give them a leaflet outlining sources of help for new mothers.

Six months later all participants in the trial will be sent a postal questionnaire including the Edinburgh Postnatal Depression Scale (EPDS), the SF-36, (an extensively used and validated measure of physical, social and emotional well-being) and questions taken from the 1993 Survey of Recent Mothers regarding satisfaction with care (see attached copies of the questionnaire instruments).

Community Intervention Trial:

In this trial 14 local government areas (LGAs) will be selected, forming 7 matched pairs, with one LGA in each pair receiving the intervention program and the other becoming a control community for comparison purposes.

The intervention program will have two components, one directed to primary care, the other to community services (local government and community agencies), with a steering committee locally appointed to co-ordinate the implementation of the intervention.

A. In **primary care** the aims are:

* increased recognition of depression in mothers of young children at all primary care contacts;
* an active response to the recognition of depression by primary care providers;
explicit offer of time to talk by both MCHNs and GPs;
* increased recognition and treatment of physical problems which are common in the year after birth (eg mastitis, low back pain, sexual problems)[46]

The strategies will include:

i) provision of a training program for MCHNs and GPs, comprising information about the prevalence, associated factors and implications of depression after birth and health after childbirth generally, as well as involving skills training in non-directive counselling/active listening

ii) the establishment of professional peer support programs for both MCHNs and GPs

iii) with the assistance of the steering committees, local government and the general practice divisions, the development of networks between those in different primary care roles (GPs and MCHNs) and with existing self-help groups; the development of links between the primary care network and the local community psychiatric services so that support can be offered to mothers in a context where consultation, liaison and referral are readily available for GPs and MCHNs.

B. The range of activities with **local government and community agencies** to increase the availability of support and 'time out' for recent mothers will include:

i) an assessment of the availability of relevant services (occasional child care, recreational services, library, information and counselling services, neighbourhood houses, community centres and community health centres, as well as shopping centre facilities), their accessibility and the extent to which they are 'mother and baby friendly'.

ii) the development of an information kit for mothers comprising a listing of local services for mothers and babies, a brochure outlining some of the common difficulties of being a mother and some strategies for dealing with these which other women have found helpful, an information ("useful tips") sheet for fathers and a booklet of free service vouchers to recent mothers. The latter might for example include session(s) at the local occasional childcare service, free entry at the local swimming pool, a series of relaxation classes at the Maternal and Child Health Centre, etc. (The specific nature of such vouchers would be dependent on what each LGA considered appropriate and feasible.) The kit would be given to all recent mothers by the MCHN during the home visit made soon after hospital discharge.

iii) the establishment of a mother-to-mother support network based on the principle of non-professional befriending[31]. Such a network could take many forms, depending on the nature of the community, and it is proposed that the steering committee in each intervention community would choose the appropriate model. Various possibilities are likely:

- calling for volunteer older women to provide support to recent mothers via a monthly visiting program ("grandmother" scheme) perhaps co-ordinated through the council's Child and Family Services Department or the local neighbourhood house
- MCHNs offering to put two women who have babies the same age in touch with each other for mutual support such as occasional babysitting, getting out together etc. ("peer" support model)
- developing a pool of mothers with older children who could "adopt" a mother with a new baby for advice and support ("experienced" mothers model).

Referral of new mothers to such a network might be either self-referral (local advertising of the network once established would be required) or via the MCHN or GP. Again this likely to depend on the model chosen in each area.

Process evaluation to ensure that the intervention program is functioning adequately before impacts and outcomes are measured[54] would be undertaken in each intervention community via postal questionnaires to a random sample of 100 mothers and all MCHNs and GPs. This would occur at two time points during the trial (12-18 months after initial establishment and then again 12-18 months later) to assess whether all elements of the intervention are in operation, and continue to function over time. At the second time point a further workshop would also be offered to all MCHNs and GPs enabling them to review the implications of the previous training on their practice since. Another component of the process evaluation would involve interviews with steering committee members and other key informants in each of the intervention communities.

Assessment of depression and of general health would be made using the EPDS and the SF-36 mailed out to mothers 6 months

after birth by their local councils. The only identification on these questionnaires will be the local government birth registration number. Researchers at the centre would thus not have access to individual women's names and addresses. A reminder postcard would be mailed two weeks later, a strategy that is likely to achieve a response rate of at least 67%.

All women scoring as depressed on the 6 month EPDS as well as a random sample of those scoring as not depressed would also be sent an EPDS and an SF-36 two years after birth. The inclusion of a random sample of mothers not depressed would ensure confidentiality regarding women's depression status when the list of local government birth registration record numbers for women to be sent second questionnaires is given to local councils for the second mail out to mothers. Thus, local council officers would not be aware of women's depression status, nor would researchers at the Centre have any knowledge of women's names and addresses at any stage.

5. Give the number, type and age range of all participants including controls

Debriefing Trial:

The focus of the trial is women who have had an assisted delivery (caesarean section or forceps) as public patients in one of the three Melbourne teaching hospitals. The number of women to be approached for participation will be 1040 which allows for a 5% refusal rate and a possible loss to follow-up of 20% to achieve a sample size of 416 in each group, which is adequate to assess the primary aim of reducing depression 6 months after birth in women having an assisted delivery from an expected 24% in the control group to 16% in the intervention group.

Community Intervention Trial:

Just under 10,000 (4,800 each in intervention and control communities) recent mothers will be needed to respond to the outcome questionnaires in order to achieve a satisfactory sample size to detect a reduction in the community prevalence of depression of 3%. The selection of LGAs in terms of their annual number of births and the length of the data collection period will be determined taking this required sample size into account. All women who have babies during the data collection period in each of the intervention and control communities will be sent the questionnaires six months after the birth; and all those scoring as depressed and a random sample of those not scoring as depressed will be sent the questionnaires again when their babies are two.

6. Source and means of recruitment

Debriefing Trial:

Women who have had an assisted delivery at one of the three Melbourne teaching hospitals (yet to be determined) will be approached on the postnatal ward by an experienced midwife and invited to participate in the trial a day or so prior to discharge. Once the trial has been explained to women and their written consent gained, each participant will then be randomly assigned to the intervention or control group via a process of balanced block randomisation with telephone assignment at the Centre for the Study of Mothers' and Children's Health. To achieve the desired sample size recruitment is expected to take 27 months, based on expected operative delivery rates.

Community Intervention Trial:

There are two aspects to recruitment in this trial: recruitment of communities and the participation of individual women in outcome measurement. First, LGAs will be approached about their willingness to participate in the trial and given a full explanation of the nature of the proposed intervention program and of the process of randomisation into either the intervention or control group, with agreement being required from the relevant local council and their Family and Community Services Department.

In matching pairs of communities before randomisation within the pairs, consideration will also be given to sociodemographic characteristics, population size and distribution, geographic location and levels of activity in relation to community services in general and with regard to depression after birth in particular.

General Practice Divisions will also be approached and informed about the nature of the trial and their willingness to be involved in supporting the training program for general practitioners will be ascertained.

Once the trial is underway and the intervention program is established in 7 of the 14 communities, all recent mothers in all 14 communities having babies in an 18 month data collection period will be sent a letter explaining the trial and asking for their participation in completing and returning the EPDS and the SF-36 to assess their well-being 6 months after birth. This letter will also include a telephone number for contacting the research team should women wish to know anything further about the trial (see explanatory letter attached).

7. Will any special relationship exist between the recruiter and the participants?

No

8. Criteria for exclusion

Debriefing Trial:

Women who have had a stillbirth or early neonatal death, are suffering from a psychotic episode post-birth, are unwell themselves or who have babies in neonatal or special care nurseries, will not be approached to participate in the study, to avoid any possible extra distress. Women of non-English speaking background will also be excluded for the reasons already outlined (Q2). In addition, before questionnaires are sent to mothers at six months, hospital records will be checked to ensure that no mother who has had a neonatal death after hospital discharge, is sent a questionnaire.

Depending on which teaching hospital is selected for participation, it may be necessary to exclude private patients (the Royal Women's Hospital for example, does not allow any research with private patients) or special arrangements may need to be made to request doctors' permission for access. This will be determined once the hospital has been chosen.

Community Intervention Trial:

All women whose babies have been registered on local government birth registration systems during the data collection period will be sent the questionnaires to complete 6 months after the birth, with no mothers being actively excluded from this process. However, there is likely to be a low response rate from women from non-English speaking backgrounds to questionnaires in English, (and translations of the instruments have not been validated) so that the outcomes of the trial will not be able to be generalised to women from these backgrounds.

9. Details of any proposed payment

There will be no payment of participants in either trial.

10. Where will the procedures involving humans be undertaken?

Debriefing Trial:

As stated previously women will be visited on the postnatal ward of one of the three teaching hospitals. Women receiving the debriefing intervention will talk with the midwife in a space where privacy can be assured (ie not at their bedside if in a shared ward). Women in the control group will be visited

at their bedsides and provided with information about community support services.

Questionnaires sent to women will be mailed to them at their home address for return to the Centre in reply paid envelopes.

Community Intervention Trial:

The intervention strategies outlined in the research plan will all take place in community settings which recent mothers access frequently in the postnatal period, with the exception of the mothers' information kit which will be given to women personally by the maternal and child health nurse at the home visit made after hospital discharge.

The questionnaires assessing women's well-being will be posted to women at home for return to the Centre in reply paid envelopes.

11. What facilities are available for dealing with contingencies?

Not applicable

12. (a) Will radioactive substances be used?

Yes / No (No circled)

(b) Will the use of recombinant DNA techniques, toxins, mutagens, teratogens or carcinogens be involved?

Yes / No (No circled)

INFORMED CONSENT

13. Who will explain the project to the potential participant?

Debriefing Trial:

The trial will be explained by the midwife researcher.

Community Intervention Trial:

Members of the research team will explain the trial to local councils and other involved groups, such as general practitioners and Maternal and Child Health nurses. Explanations of the trial to individual women will be made in the covering letter sent with questionnaires.

14. Is there a special relationship between the person explaining the project, or any of the investigators, and a participant?

No.

15. When will the explanation be given?

Debriefing Trial:

As previously stated, the midwife will approach women on the postnatal ward and explain the nature of the trial before asking women to participate.

Community Intervention Trial:

The trial will be explained to interested local councils in the early development phase of selecting LGAs for the study and to individual women in a letter at the time they are sent questionnaires to complete (See also Q16).

16. Will the participants be capable of giving consent themselves?

Debriefing Trial:

Yes, see Information Sheet and Consent Form (Attachment).

If not, why? To whom will the project be explained and who will give consent?

In the Community Intervention Trial the project will be explained to local councils and relevant local government officers and consent to be involved in the trial will be obtained at that community level rather than via individual consent, which is not feasible in a trial of this nature.

The participation requested from individual women involves them in voluntarily completing questionnaires posted to them at home and returning them to the Centre. Thus, individual women are able to make a decision about their participation at this point and are free not to complete or return questionnaires if they do not wish to do so. This will also be made clear in the letter they receive accompanying the questionnaires (see attached explanatory letter).

17. Will written consent be obtained from all participants?

If not, please give reasons

Debriefing Trial:

Yes (circled) / **No**

Community Intervention Trial:

In negotiating agreement to participate with LGAs, the research team will provide to local councils and their nominated officers a written explanation of the research plan detailing:

i) what is expected of intervention and control communities

ii) a careful discussion of the ethics of randomisation including that agreement to participate involves an agreement to abide by the outcome of the randomisation to intervention or control community

iii) a timeline for the trial

iv) a description of the extent of support (financial, staff and otherwise) available from the project, and

v) an outline of the processes for disseminating the results of the trial to the communities involved.

Meetings would also be held with interested councils, council staff, local community representatives and health professionals to discuss the trial and answer any questions before LGAs agreed to participate. A written agreement outlining the basis for the participation would then be signed by each participating council.

In relation to the consent of individual women, it is proposed that voluntary completion and return of the questionnaires be taken as consent to participate, a process outlined as acceptable in the "Guidelines for Obtaining Informed Consent" received with this application form.

18. Who will act as witness?

Debriefing Trial:

In this trial, given the low risk nature of the planned intervention and the fact that the purpose and nature of the research are straightforward to convey to women, it is proposed that both the woman and the midwife sign the consent form, with the midwife signing a statement that she has fully explained the project to the woman concerned (see Consent Form attached), and that this process take the place of calling in a third party to witness the woman's consent.

Community Intervention Trial:

Given the issues discussed in Question 17, a witness's signature is not seen as feasible, nor applicable, to the consent issues for this trial.

SECTION B - ETHICAL CONSIDERATIONS

19. How will information be handled to safeguard confidentiality both during and after completion of the research project?

During both trials, computer data on Centre computers will only be accessible at the Centre with security passwords. Identifying information about individual participants will be stored separately from questionnaire responses. All paper records will be stored in a physically secure area within the Centre. After the conclusion of the research, essential data to be kept will be stored in this secure area for the required periods, with remaining data destroyed according to the Centre's data destruction policy with details of this process being kept at the Centre.

20. What demands, inconvenience or discomfort will be involved? Explain the possible dangers, risks or ill effects of these procedures and the precautions to be taken to prevent or minimise them?

We believe that both these trials pose very low levels of risk or ill effects to the women involved. In the Debriefing Trial, the lack of opportunity to discuss the events and experiences of birth with someone prior to going home from hospital is something which previous research indicates women find unsatisfactory about their hospital experience. It is acknowledged that discussing the birth may be distressing for some women and for this reason the midwife working on the project is someone with considerable counselling experience. A protocol for sensitively handling such situations is attached.

In the Community Intervention Trial the risks to individual women of completing and returning questionnaires about their physical and emotional well-being are also considered to be low. It is possible however, that filling in the questionnaires could be upsetting for some women who are feeling depressed. For this reason, the explanatory letter encourages women to contact the research team if they wish to talk about any issues the questionnaires may raise for them.

The steering committees in each of the intervention communities will also play an important monitoring role in terms of the impact of the intervention strategies in their communities, as will the formal strategies for process evaluation outlined in the grant application attached.

21. Are there any other ethical issues raised by the proposed project? What is your response to them? In many research projects involving humans there is trade-off to be made between the cost of interventions to those participating in them (eg in terms of discomfort, health risk, loss of privacy etc.) and the value to be achieved by carrying out the research. The Committee must be in a position to evaluate clearly that trade-off.

The key ethical issues in randomised clinical trials are:

* informed consent to participation including to randomisation, with the provisos that consent may be withdrawn at any time and that the care provided by the agency and its staff will not be influenced by decisions about participation

* genuine uncertainty about the benefits of the proposed intervention

* an adequate sample size to test the hypothesis

* an assessment of the intervention as unlikely to be harmful

The key issues in community-based trials have received less formal agreement:

* individual consent by residents is not sought but the intervention must receive approval from local agencies, including the local council.

* the proposal must satisfy the local agencies and professionals (as well as the relevant IEC) that it meets the criteria listed above for clinical trials.

We hope that we have demonstrated that in designing these two trials we have taken into account these important issues.

SECTION C - USE OF INFORMATION FROM FILES AND RECORDS

The Privacy Act applies to all "personal information" held by a "Commonwealth Agency" (Refer to Notes to Q.22 and 23 for an explanation of these terms). Research which uses any records subject to the Privacy Act must comply with its requirements for information privacy, ie. the form of the 11 Information Privacy Principles summarised in appendix A. IPP 11 requires that information may only be disclosed by a Commonwealth Agency to a researcher with the consent of the subject of the information. The only exception to this is where the research complies with the NH&MRC Guidelines for the Protection of Privacy in the Conduct of Medical Research, which set out a list of specific issues to be considered by researchers and the Ethics Committee. The Guidelines, are available on request from the Secretary to the Standing Committee on Ethics in Research on Humans [Ph. 905-2061]). This applies to all research, whether or not it is medical research.

NOTE THAT QUESTION 24 MUST BE ANSWERED.

Researchers should refer to the explanatory notes and must have read Appendix A before completing this section.

22 **Records to be Used**

(a) Describe the type(s) of information which will be collected eg. medical records.

- Not applicable -

(b) Who, or what organisation, holds the information?

(c) Has the organisation agreed to provide the information? (Attach a copy of letter)

(d) State any conditions imposed by the organisation on the release of information.

(e) Is any organisation listed in (b) above a "commonwealth agency"? (See Note to Q.22)

23 Personal Information and Privacy

(a) Does the data collection enable access to identifying information about any individual? (See Note to Q.23) Yes No (If no go to Q.24)

(b) If 'yes' will that individual's consent be sought by the researcher? If 'no' please give reasons. (See Note to Q.17) Yes No

(c) List the names of people who will have access to the original research data.

(d) Outline the arrangements for safeguarding individual privacy in accessing, collating, storing and publishing results.

(e) Describe the arrangements for retention of data and the eventual destruction of data.

24 **Does the Privacy Act Apply?**

If the data used are held or to be collected by a Commonwealth agency (see Q.22(e)) <u>and</u> collection will or might enable identification of any individual (see Q.23(a)) then the Privacy Act 1988 applies.

(a) Does the Privacy Act apply to the proposed data collection? Yes/No

................Judith Lumley................ ....29/5/94................
Signature of Chief Investigator Date

(b) If yes to (a), please provide details for consideration by the Ethics Committee as required by Sections 3.8 and 3.9 of the NH&MRC Guidelines. Your attention is drawn to the monitoring requirements in 3.13 - 3.16 of the Guidelines.

Declaration

I/We, the undersigned, have read the current NH&MRC Statement on Human Experimentation and the relevant Supplementary Notes to this Statement and accept responsibility for the conduct of the research detailed above, in accordance with the principles contained therein and any other condition laid down by the Monash University Standing Committee on Ethics in Research on Humans. In addition, I/we undertake to notify the Monash University Ethics Committee in writing immediately if any changes to the protocol are proposed after the approval of the Committee has been obtain. The Associate Investigator will assume responsibility for the project in the absence of the Chief Investigator.

Chief Investigator's or Supervisor's signature

Name (please print) JUDITH LUMLEY RHONDA SMALL LYNDSEY WATSON

Signature Judith Lumley Rhonda Small Lyndsey Watson Date 29/5/94

Associate Investigator's or Student's signature:

Stephanie Brown
[illegible] Date 27/5/94
30/5/94

Co-Investigator's signature/s

1. Date

2. Date

3. Date

4. Date

5. Date

Head of Department/Division

I certify that I am prepared to have this project undertaken within my department

Name (please print) JUDITH LUMLEY

Signature Judith Lumley Date 29/5/94

Department/Division Centre for the Study of Mothers' & Childrens Health

Debriefing Trial: Protocol for conducting debriefing sessions

1. Before starting, the midwife will indicate that the debriefing session may be interrupted or concluded at any time should women not wish to continue for any reason (eg to attend to their baby or if the woman is distressed).

2. The midwife will also say before beginning that giving birth is an experience which for most women has many positive aspects, but also may involve some negative ones and that reflecting on the experience may raise issues that women would like to talk more about with someone else after the session is over.

3. During the sessions, if women should become distressed and seek advice or assistance, (or at the conclusion of the session if this is more appropriate), the midwife will explore some possible avenues for obtaining further support in relation to women's individual situations. This will include: exploring with women whether they know anyone they feel able to talk to and encouraging them to do so if they feel they would understand and be supportive; discussing possible options for obtaining extra support (eg domiciliary midwifery support on discharge, extra domestic help, etc.).

4. If the midwife feels it is appropriate she will offer to facilitate putting women in contact with someone able to provide a listening ear and/or support (eg local GP, maternal and child health nurse, support groups for mothers, mother and baby units - general and psychiatric, council home help services).

5. At the conclusion of the debriefing session, all participants will be asked if they have any questions about the research or if they would like to raise any further issues not already discussed. They will also be asked whether they would like to be informed about the findings of the study.

6. Finally, women will be encouraged to ring the midwife or another member of the research team if they feel they need to have a further talk about any of the issues raised in the debriefing session.

M&C
Centre for the Study of Mothers' and Children's Health

463 Cardigan Street
Carlton Victoria 3053
Australia

Director Dr Judith Lumley

Telephone (03) 348 1211
(03) 347 0797
Facsimile (03) 348 1129

Community Intervention Trial: Explanatory Letter to Individual Participants

Dear

Please find enclosed two questionnaires which are being sent to you as part of an evaluation of community programs of support to mothers in the first six months after birth.

You are living in an area where your local council is participating in a research project either as a community which is trialling strategies of extra support to mothers or as an area which is providing the usual range of community support services.

The research is being conducted by the Centre for the Study of Mothers' and Children's Health at Monash University, with questionnaires mailed to you by your council on our behalf. All mothers who have babies over an 18 month period in fourteen different local government areas are being asked to fill in these questionnaires six months after the birth.

Your participation in this research is of course completely voluntary.

We hope however that you will find the time to complete the questionnaires and return them to us at the Centre in the enclosed reply paid envelope.

Your responses to the questionnaires are completely confidential. We do not have your name or address so these cannot be linked in any way with the replies you give. Responses to the questionnaires will only be used to help us get an overall picture of how recent mothers are feeling - physically and emotionally, six months after giving birth.

If you have any queries at all about the research or if you would like to discuss any of the issues raised in the questionnaires, please do not hesitate to contact Rhonda Small, the research co-ordinator, on 348 1047.

Thanking you

Yours sincerely

Centre for the Study of
Mothers' and Children's Health

463 Cardigan Street
Carlton Victoria 3053
Australia

Director Dr Judith Lumley

Telephone (03) 348 1211
(03) 347 0797
Facsimile (03) 348 1129

RECOVERY AFTER ASSISTED BIRTH

Description of the project

The process of recovery after an assisted birth can be longer and more difficult than it would be otherwise and we are trying to find out what can be done during the postnatal stay to contribute to a rapid and complete recovery.

You are invited to take part in a research study which compares two different methods of assisting women after assisted birth. One focuses on information about sources of practical help available in the community. The other is an opportunity to discuss the events and experiences of the birth itself. You can help us to find out which of the two is more effective by taking part in the research study in which half the women participating receive each of the two different approaches

If you agree:

* You will receive a visit from the research midwife at a mutually convenient time, in a private part of the ward, before you leave hospital.
* Her discussions will focus **either** on practical sources of help **or** on discussions about the events and experiences of birth. You have an equal chance of being in either group.
* Six months after birth you will be sent a questionnaire about your physical and emotional health (together with a stamped addressed envelope) to fill in and return to the research team.

All information collected during the project is completely confidential. The records are available only to the research team. Nothing written about the project will identify anyone who took part.

* You may withdraw from the project at any time.
* A final summary of the findings will be made available to you once the project is completed.

Whatever you decide about taking part in the study, you and your baby will get the best care that the hospital and staff can provide.

If you would like more information or to discuss any of this further contact one of the research staff:
Research midwife: Ms Lisa Donohue (03) 348 1211
Project director: Dr Judith Lumley

CONSENT FORM

FOLLOW-UP AFTER ASSISTED BIRTH

______________________________ (print name)

of ______________________________ (address)

____

____

Phone no: ______________________________

agree to take part in the research project being conducted by

______________________. The research has been fully explained to me and I have received a copy of the project information sheet.

I understand that I am free to withdraw from the project at any time. I have had the opportunity to ask questions about the project and am aware that I may contact the midwife to discuss any problems.

Signed: ______________________________

Witness: ______________________________

Date ____/____/19____

****

I have explained the nature of the research project to:

______________________ and have given her the opportunity to ask any questions.

Signed ______________________________

Date ____/____/19____

Summary of findings requested: YES/NO
