# Supplementary material for: Improving Population-Level Maternal Health: A Hard Nut to Crack? Long Term Findings and Reflections on a 16-Community Randomised Trial in Australia to Improve Maternal Emotional and Physical Health after Birth [ISRCTN03464021]
Source: PLoS One. 2014 Feb 28;9(2):e88457. doi: 10.1371/journal.pone.0088457 (PMC3938427; doi:10.1371/journal.pone.0088457)
Supplement: PRISM Ethics S2 — Trial ethics approval from La Trobe University. (PDF) [file pone.0088457.s003.pdf]

COPY 5.27030

LA TROBE
UNIVERSITY
Opportunity for Excellence

TO: Ms Rhonda Small, Centre for the Study of Mothers' and Children's Health

SUBJECT: Review of Human Ethics Committee Application No. 96/62: *PRISM: Program of Resources , Information and Support to Mothers.*

DATE: 24 July 1996

The Chairperson of the Human Ethics Committee (HEC), Dr Carl Parsons, has reviewed your application for ethics approval. The above named project has been assessed as complying with the National Health and Medical Research Council's *Statement on Human Experimentation and Supplementary Notes* and with University guidelines on *Ethics Approval for Research with Human Subjects* and has been granted ethics approval. The project has been approved from 24 July 1996 to 24 July 1997.

Please note that your application has been reviewed by the Chairperson of the HEC in the interest of facilitating a decision on your application before the next committee meeting. The decision to approve your project will need to be ratified by the full HEC and consequently approval for your project *may* be withdrawn or conditions of approval altered. However, your project may commence prior to the ratification of the approval decision. You will be notified if the approval status of your project is altered.

The following standard conditions apply to your project:

a. Complaints. If any complaints are received or ethical issues arise during the course of the project, researchers should advise the Chairperson of the La Trobe University Human Ethics Committee on telephone (03) 9479 1443;
b. Limit of Approval. Approval is limited strictly to the research proposal as submitted in your application while taking into account the conditions and approval dates outlined above;
c. Variation to Project. As a consequence, any subsequent variations or modifications you may wish to make to your project must be notified formally to the Committee for further consideration and approval using the appropriate form (*Application for Approval of Modification to Research Project*) which is available from the Research and Graduate Studies Office. If the Human Ethics Committee considers that the proposed changes are significant, you may be required to submit a new application for approval of the revised project; and
d. Progress Reports. You are required to submit a *Progress Report* form annually, if your project continues for more than 12 months, and at the conclusion of your project. The form can be collected (electronic or hard copy) from and, when completed, should be returned to Secretary of the HEC. Failure to submit a progress report will mean approval for this project will lapse.

If you have any further queries on these matters or require any clarification please contact me at the Research and Graduate Studies Office on telephone 9479 1443, facsimile 9479 1464 or e-mail address L.Jelicic@latrobe.edu.au.

L. Jelicic

Lebecca Jelicic
Secretary, LTU Human Ethics Committee

5.013.082

13

NHMRC Project Grants
INSTITUTIONAL APPROVAL FORM FOR
RESEARCH INVOLVING HUMANS

One (1) copy of the completed approval form should be either attached to the original application or sent separately to the Secretary of Council no later than 31 July.

APPLICANT USE Please complete in BLACK type or ink only

Chief Investigator

	Surname	Title	Initials
A	LUMLEY	PROF	J
B	SMALL	MS	R
C	BROWN	MS	S
D	WATSON	MS	L

Scientific Project Title:

PROGRAM OF RESOURCES, INFORMATION AND SUPPORT TO MOTHERS: A COMMUNITY TRIAL

Administering Institution

LA TROBE UNIVERSITY

ETHICS COMMITTEE USE

Does this Project comply with the provisions contained in the Council's document 'Statement on Human Experimentation and Supplementary Notes' ? Y/N ☐

Does this Project comply with the regulations governing experimentation on humans within your Institution and within your State or Territory? Y/N ☐

Comments, provisos or reservations:

NIL

Name of responsible Ethics Committee

LATROBE UNIVERSITY HUMAN ETHICS COMMITTEE

Name of Ethics Committee representative (Block letters):

Surname	Title	Initials
JELICIC	MS	L

Signature: K. Jelicic Date: 1/8/96

Note: (1) This form has been produced in an effort to standardise and effectively record ethics approval for all projects submitted to the NHMRC. Should it prove inappropriate, an individual statement may be forwarded in lieu. As the Council cannot recommend support if ethics clearance is not provided, it is of utmost importance that this information is received.

(2) If there is no appropriate Ethics Committee at the institution concerned, the Head of Department, or, in the case of individual researchers, the applicants themselves, should ensure that the proposal is submitted to an established Ethics Committee at a hospital or university for consideration prior to completing and signing the rest of the form as an undertaking that the provisions of the NHMRC 'Statement on Human Experimentation and Supplementary Notes' will be observed.

LA TROBE UNIVERSITY
Opportunity for Excellence

MEMORANDUM

TO: Professor Judith Lumley & Ms Rhonda Small, Centre for the Study of Mother's and Children's Health

FROM: Lebecca Jelicic. Secretary, University Human Ethics Committee

SUBJECT: HEC Application 96/62 *PRISM: Program of Resources, Information and Support to Mothers*

DATE: 29 July 1997

This is to advise that the ethics approval period for the above named ethics application has been extended to 31 December 2002, as requested the progress report you submitted on 15 July 1997.

If you would like further clarification of this matter please contact me on telephone x1443 or e-mail L.Jelicic@latrobe.edu.au.

L. Jelicic

Lebecca Jelicic

NHMRC Project Grants
INSTITUTIONAL APPROVAL FORM FOR RESEARCH INVOLVING HUMANS

One (1) copy of the completed approval form should be either attached to the original application or sent separately to the Secretary of Council no later than 31 July.

APPLICANT USE Please complete in BLACK type or ink only

Chief Investigator

	Surname	Title	Initials
A	LUMLEY	PROF	J
B	SMALL	MS	R
C	BROWN	MS	S
D	WATSON	MS	L

Scientific Project Title:

PROGRAM OF RESOURCES, INFORMATION AND SUPPORT TO MOTHERS: A COMMUNITY TRIAL

Administering Institution

LA TROBE UNIVERSITY

ETHICS COMMITTEE USE

Does this Project comply with the provisions contained in the Council's document 'Statement on Human Experimentation and Supplementary Notes'? Y/N Y

Does this Project comply with the regulations governing experimentation on humans within your Institution and within your State or Territory? Y/N Y

Comments, provisos or reservations:

NIL

Name of responsible Ethics Committee

LA TROBE UNIVERSITY HUMAN ETHICS COMMITTEE

Name of Ethics Committee representative (Block letters):

Surname JELICIC Title MS Initials L

Signature: L Jelicic Date: 29/7/97

Note: (1) This form has been produced in an effort to standardise and effectively record ethics approval for all projects submitted to the NHMRC. Should it prove inappropriate, an individual statement may be forwarded in lieu. As the Council cannot recommend support if ethics clearance is not provided, it is of utmost importance that this information is received.

(2) If there is no appropriate Ethics Committee at the institution concerned, the Head of Department, or, in the case of individual researchers, the applicants themselves, should ensure that the proposal is submitted to an established Ethics Committee at a hospital or university for consideration prior to completing and signing the rest of the form as an undertaking that the provisions of the NHMRC 'Statement on Human Experimentation and Supplementary Notes' will be observed.

29/07/97 15:29 LATROBE UNI -RESEARCH SERVICES - → CSMCH NO.369 P03

5.013.001

OFFICE USE ONLY

File

NHMRC
1996

IN CONFIDENCE

National Health and
Medical Research Council

PUBLIC HEALTH RESEARCH AND DEVELOPMENT COMMITTEE APPLICATION FOR A RESEARCH PROJECT GRANT

1 Chief Investigators:
Chief Investigator A will be considered the contact point for the Project Grant and will be understood to be acting for and in concurrence with all Investigators.

	Surname	Title	Given Name
A	Lumley	Dr	Judith
B	Small	Ms	Rhonda
C	Brown	Ms	Stephanie
D	Watson	Ms	Lyndsey

2 (a) Scientific Project Title:
State scientific title of project, be concise but informative. DO NOT exceed 75 characters including spaces.

Program of Resources, Information and Support to Mothers: A Community Trial

(b) Lay Project Title:
Provide a simple lay title for the project, for use in future media releases if required. DO NOT exceed 75 characters. Please complete in full – DO NOT INDICATE 'As Above'.

PRISM: Program of Resources, Information and Support to Mothers

3 (a) Administering Institution
(Full name and address required)

La Trobe University
Bundoora
Postcode 3083

(b) Institution Where Project Will Be Carried Out:
(if more than one institution, please indicate where project will be primarily conducted.)

Department / Institution: Centre for the Study of Mothers' and Children's Health, La Trobe University
Postcode 3083

4 Other Funding Agencies:

(a) Please complete below if seeking support for this Project from any other funding agency. e.g. NHMRC RADGAC, AKF, NHF, ARC, ATSIC, DEET etc.

Name and address of Agency:

VicHealth, 333 Drummond Street, Carlton Vic 3053

GPEP, Commonwealth Dept of Human Services and Health, Canberra ACT 2600

(b) Disclosure of Application to other Funding Agencies? Y/N: Y

5 Research Classification : Please see Instruction booklet for category description

a) Broad Research Area. (1 only to be selected from Category A) 005

b) General Grouping for Assigners' Panel consideration (1 only to be selected from Category B) 048

c) Specific Discipline Classification (identify at least 3 and up to 10 disciplines from Category C in descending order of relevance to your research)

(i)	970	(ii)	805	(iii)	325	(iv)	825	(v)	450
(vi)	637	(vii)		(viii)		(ix)		(x)	

d) Keywords DO NOT exceed 20 characters including spaces.

(i)	Community Study	(iv)	Physical Health
(ii)	Randomised Trial	(v)	Primary Care
(iii)	Maternal Depression		

e) Socio-Economic Objectives

130201	130208	130214	130215	
30%	30%	30%	10%	

6 Other General Questions: Please answer all questions

a) If research involves humans -
i. Sample size?
ii. What is the ratio of males to females in the study?

i 9600
ii 0 : 1

Y/N

(b) Is this an HIV/AIDS (CARG) application? N

(c) Is this a DVA application? N

(d) Number of years requested: 5

7 SYNOPSIS

Please give a brief description of the project
This should be a clear, stand-alone, summary of the context, objectives, methods and likely benefits of the project.
NOTE: It will be used by the granting committee to select assessors.

Context
The prevalence of depression in women after childbirth has been reliably shown to be in the region of 10-20% at any time in the first twelve months after birth. A Victorian population based study conducted in 1989 found the prevalence to be 15.4% (95% CI 12.8-18.0%) 8-9 months after birth. A repeat of the study in 1993 found a prevalence at 6-7 months of 16.9)95% CI 14.9-18.9). The significance of this public health problem for women, their children and the community has been recognised by inclusion in the national health goals and targets of the goal of reducing the severity and duration of maternal depression in the first postnatal year. There is now also evidence for significant physical morbidity in mothers after birth and associations between maternal depression and physical health problems have also been found.

A major component of the Centre's research program since its inception has been focused on the question of maternal depression: two Victorian population based prevalence studies have been carried out and a follow up study of women's experiences of depression, exploration of associated factors and sources of assistance was completed in 1993. Ongoing work at the Centre includes a study exploring satisfaction with maternity care and issues of emotional well-being in Vietnamese, Filipino and Turkish mothers, and the 1993 Survey of Recent Mothers and the Life as a Mother Project include more detailed assessment of use of services in the postnatal period and of the physical sequelae of childbirth. Each of these projects will inform the ongoing development of the proposed project as results become available. Our research on women's experience of depression indicates that a minority of women seek help when depressed, that those who do, turn to primary health care providers, that when help is sought acknowledgment of the common difficulties of motherhood is often lacking, and that having someone to talk to who listened and understood is of major assistance in recovery, as is having timeout from caring for children. There is evidence from our own work and that of others demonstrating considerable maternal physical morbidity in the first postnatal year combined with a lack of help seeking and poor detection of problems by care providers.

Aim
The principal aim of this Program of Resources, Information and Support to Mothers (PRISM) is to reduce the prevalence of depression and of physical health problems in mothers 6-9 months after birth and to reduce the proportion of women still depressed 18-21 months after birth in communities randomised to receive an intervention program.

Methods
PRISM is a randomised trial of community based strategies to increase recognition of depression in all primary care contacts, to increase listening skills and explicit offers of 'time to talk' by GPs and maternal and child health nurses, to improve recognition and treatment of common postnatal physical health problems in primary care; to increase the availability of support and 'timeout' for recent mothers through a review of the availability and accessibility of relevant services, an information kit for new mothers and the establishment of mother-to-mother support networks based on the principle of non-professional befriending.

Randomisation will be of 14 local government areas, within pairs, with pairs stratified on important covariates. Envisaged as a five year developmental program, process and impact evaluation will occur - to document and assess the different program elements and to enhance the reproducibility of the program if successful - prior to outcome evaluation of the program aims, which will occur in intervention and control communities via postal survey including the Edinburgh Postnatal Depression Scale and the SF-36 self report health questionnaire.

Benefits
The benefits of the project, if successful, include positive outcomes for the well-being of women, children and families, and thus for the community at large; and the development of clearly defined, relatively low-cost strategies which can be readily incorporated in ongoing programs of care and support to all mothers. In addition, the trial provides relevant skills enhancement for primary care providers also of benefit to other clients, and develops a model for joint action on a significant public health issue between the primary care sector, local government and community agencies, a strategy which if successful has important implications for other mental health and human service programs.

Please Note: This page will not be included in material sent to external Project Grant assessors

8 Nomination Of Assessors:

Chief Investigator A As for Q. 1.

Surname	Title	Initials
LUMLEY	DR	JM

Scientific Project Title As for Q. 2 (a)

Program of Resources, Information and Support to Mothers: A Community Trial

(a) Nomination Of Suitable Assessors

i)

Surname	Title	Initials
HAWE	DR	P

Discipline and area of expertise: COMMUNITY STUDIES, PUBLIC HEALTH

Address: DEPT OF PUBLIC HEALTH
EDWARD FORD BUILDING (A27)
UNIVERSITY OF SYDNEY NSW 2006

ii)

Surname	Title	Initials

Discipline and area of expertise

Address

iii)

Surname	Title	Initials

Discipline and area of expertise

Address

9 Chief Investigators

A

Surname	Title	Initials
LUMLEY	PROF	JM

Current Appointment	STD	Contact Phone No.	Ext.
DIRECTOR	03	9348 1211	–

Current work contact address:

Department: CENTRE FOR THE STUDY OF MOTHERS' & CHILDREN'S HEALTH [From 1/6/96]

Institution: Suburb: Postcode:

Most Recent and Highest Academic Qualifications:

Year	Conferring Institution	Degree
1991	RACP	FAFPHM
1972	MONASH UNIVERSITY	PhD
1966	MONASH UNIVERSITY	MBBS

Percentage of working time to be devoted to:

This project 20 % Other NHMRC research projects 10 % All other research projects 70 %

Should Investigator be absent during the Project Grant period complete the following:

Period of absence	Reason

B

Surname	Title	Initials
SMALL	MS	RE

Current Appointment	STD	Contact Phone No.	Ext.
RESEARCH FELLOW	03	9348 1047	–

Current work contact address:

Department: CENTRE FOR THE STUDY OF MOTHERS' & CHILDREN'S HEALTH

Institution: LA TROBE UNIVERSITY 463 CARDIGAN ST Suburb: CARLTON Postcode: 3053

Most Recent and Highest Academic Qualifications:

Year	Conferring Institution	Degree
1995	UNIVERSITY of MELBOURNE	Dip Epid
1981	RMIT	Grad Dip Lib
1974	UNIVERSITY of MELBOURNE	BA

Percentage of working time to be devoted to:

This project 90 % Other NHMRC research projects 5 % All other research projects 5 %

Should Investigator be absent during the Project Grant period complete the following:

Period of absence	Reason

C

Surname BROWN **Title** MS **Initials** S J

Current Appointment RESEARCH FELLOW **STD** 03 **Contact Phone No.** 9348 1047 **Ext.** -

Current work contact address:

Department CENTRE FOR THE STUDY OF MOTHERS' & CHILDREN'S HEALTH

Institution LA TROBE UNIVERSITY 463 CARDIGAN ST **Suburb** CARLTON **Postcode** 3053

Most Recent and Highest Academic Qualifications:

Year	Conferring Institution	Degree
1982	UNIVERSITY of MELBOURNE	BA (hons)

Percentage of working time to be devoted to:

This project 90 % Other NHMRC research projects 5 % All other research projects 5 %

Should Investigator be absent during the Project Grant period complete the following:

Period of absence	Reason

D

Surname WATSON **Title** MS **Initials** L

Current Appointment SENIOR RESEARCH FELLOW **STD** 03 **Contact Phone No.** 9348 1211 **Ext.** -

Current work contact address:

Department CENTRE FOR THE STUDY OF MOTHERS' & CHILDREN'S HEALTH

Institution LA TROBE UNIVERSITY 463 CARDIGAN ST **Suburb** CARLTON **Postcode** 3053

Most Recent and Highest Academic Qualifications:

Year	Conferring Institution	Degree
1990	LA TROBE UNIVERSITY	MSc
1963	UNIVERSITY OF MELBOURNE	BSc

Percentage of working time to be devoted to:

This project 10 % Other NHMRC research projects 5 % All other research projects 85 %

Should Investigator be absent during the Project Grant period complete the following:

Period of absence	Reason

10 Participants Other Than Those Included in Questions 9 and 14

NB: Documentary evidence from all parties is required to confirm any collaborative study.

(a) Associate Investigators

I, named below as an associate investigator on this project, certify that I have agreed to participate, and intend to devote the following number of hours to this collaboration:

	Surname	Title	Initials	Department	Institution
(i)	GUNN	DR	J.M.	PUBLIC HEALTH + COMMUNITY MEDICINE	UNIV OF MELBOURNE
	**Hours per week**	**Signature**	**Date**	**Qualifications**	
	2	J M Gunn	22.2.96	MBBS, DRACOG, FRACGP	
(ii)	Surname	Title	Initials	Department	Institution
	Hours per week	Signature	Date	Qualifications	
(iii)	Surname	Title	Initials	Department	Institution
	Hours per week	Signature	Date	Qualifications	

(b) Research Students / Technical Staff

State qualifications sought / role in Project

	Surname	Initials	State qualifications sought / role in Project
(i)	DAWSON	W	ADMIN OFFICER
(ii)	BEALE	D	ADMIN ASSISTANT
(iii)			
(iv)			
(v)			

Certification by Chief Investigators, Head of Department and Head of Organisation.

Signatures of Chief Investigators

In signing this page, you certify that all details given in this application are correct and you agree to carry out the project in strict accordance with the current NHMRC 'Conditions of Award for Project Grants'.

		Date
A	Judith Lumley	16/2/96
B	Rhonda Small	27/2/96
C	Stephanie Brown	27/2/96
D	Lyndsey Watson	27/2/96

Certification by Head of Department/Head of Research Committee

I certify that the project is appropriate to the general facilities available and that I am prepared to have the project carried out strictly in accordance with the current NHMRC 'Conditions of Award for Project Grants'.

Use Block Letters

Surname	Title	Initials	Department
WATSON	MS	LF	CENTRE FOR THE STUDY OF MOTHERS' & CHILDREN'S HEALTH

Signature	Date
Lyndsey F Watson	27/2/96

Certification by Head of Administering Organisation

(Head of Organisation or nominee)

I certify that this request satisfies all the requirements of this Organisation.

Use Block Letters

Surname	Title	Initials	Appointment

Department (if applicable)	Organisation

Signature	Date

12 Funding will be dependent on ethical clearances
Clearance Requirements [It is essential that each part is answered]

(a) Research involving humans - Please mark Y/N

	Y/N
(i) Does this project include research involving humans? If yes Complete Q 9 (b)	Y
(ii) Does this project involve the administration to humans, of drugs, chemical agents or vaccines?	N
(iii) With regard to privacy, does this project involve the use of personal information obtained from a Commonwealth department or agency (including Repatriation Hospitals)? If yes, specify the name of the department or agency.	N
(iv) If Yes to any of the above is the completed FINAL clearance form attached? Provisional clearances will not be accepted.	N

If the project involves experimentation or investigation on animals or research involving organisms being genetically manipulated or the use of potent teratogens or carcinogens, please contact the NHMRC Secretariat for advice/forms as appropriate.

Note: 1 copy of the **final** clearance must be forwarded to the Secretary of Council no later than 31 July if not available at the time of submission of this application. Failure to ensure that these requirements are met will affect funding of the application if successful.

(b) Ethical Implications of the Project - Research Involving Humans

The key ethical issues in randomised clinical trials are:

* informed consent to participation including to randomisation, with the provisos that consent may be withdrawn at any time and that the care provided by the agency and its staff will not be influenced by decisions about participation
* genuine uncertainty about the benefits of the proposed intervention
* an adequate sample size to test the hypothesis
* an assessment of the intervention as unlikely to be harmful

The other ethical issues which apply here are those relating to privacy and confidentiality : information about the participants to be stored securely (both computer security and a geographically secure area), identifying information to be separated from other data, only aggregated data to be published.

The key issues in community-based trials have received less formal agreement: individual consent by residents is not sought but the intervention must receive approval from local agencies, including the local council. The proposal must satisfy the local agencies and professionals (as well as the relevant IEC) that it meets the criteria listed above for clinical trials.

14 Aims and Objectives

What are the specific, quantifiable objectives of the project? Describe how the Research Plan will address the objectives.

Aim

To reduce the prevalence of depression and of physical health problems in mothers 6-9 months after birth and to reduce the proportion of women still depressed 18-21 months after birth in communities randomised to receive an intervention program.

Strategy Objectives

To conduct a developmental training program for primary health care providers, particularly GPs and Maternal and Child Health Nurses, providing information about recent research on the experience of depression after childbirth, about the common physical health problems mothers experience, and of simple intervention strategies found to be helpful.

Via such developmental programs to increase the availability and accessibility of 'someone to talk to' for recent mothers in the intervention communities.

With the assistance of local government and community agencies to assess and document existing support services to recent mothers at the local community level.

To provide recent mothers, soon after birth, with accessible information about the common difficult experiences of early motherhood (fatigue, low emotional wellbeing, physical health problems) and about the range of local support services available to them.

To encourage local government and community agencies to offer incentives to recent mothers to take up available support services, via the distribution to all recent mothers of free vouchers or discounts for services, thereby increasing community acknowledgement of mothers' needs for 'timeout'.

To establish mother-to-mother support programs with the help of maternal and child health and local agencies.

15 Benefits

What Public Health benefits can be expected from the project either directly or by subsequent application of results? Please comment on the relevance of the research topic.

The project addresses the problem of maternal depression after childbirth, a problem that is now commonly agreed to be a major primary care condition, and tests strategies for improving physical health and reducing depression after birth in the community setting. The design of the project as a randomised controlled trial enables effective evaluation of the intervention. If successful, immediate benefits will be achieved for the women involved in terms of improved emotional well-being which can be expected to have positive flow-on effects for their children and families. Beyond this, the strategies for reducing depression are characterised by relative simplicity enabling ready implementation within standard models of caring for mothers and babies in the postnatal period. The trial provides relevant skills enhancement for primary care providers also of benefit to other clients, and develops a model for joint action on a significant public health issue between the primary care sector, local government and community agencies, a strategy which if successful has implications for other mental health and human service programs.

It is acknowledged that the generalisability of the findings will be limited largely to women of English speaking backgrounds given that the instruments used to measure outcomes will require a capacity to read and write in English. The Centre is currently undertaking a study involving Vietnamese, Turkish and Filipino mothers which is exploring their experiences of maternity care and life with a baby, including their experiences of depression in the postnatal period. It is hoped that the findings of this study will provide sufficient information about the emotional well-being of mothers of non-English speaking backgrounds to ascertain whether the strategies to be trialled here are also likely to be appropriate in these groups.

Depression - prevalence after birth

The issue of depression among women in the first year after the birth of a child has received increasing attention from researchers and policy makers in the last 5 years. The point prevalence of depression in a 1989 population-based sample of 790 Victorian women, 8 to 9 months after birth, assessed with the Edinburgh Postnatal Depression Scale (EPDS score 13 or more) was 15.4% [95% confidence interval 12.8 to 18.0%][1]. Very similar rates have been reported, using the same scale, in a regional study from New South Wales six months after birth (16.3% [95% CI 12.5 to 20.1][2]. The EPDS has a sensitivity of 86%, 95%, 68% and a specificity of 78% 93% 96% when assessed against psychiatric diagnosis in three community settings[3-5]. Using a different measure (the Beck Depression Inventory) in a longitudinal hospital-based study of first births to married, English speaking women in Victoria the point prevalence of depression 4 months after birth was lower but within the same confidence interval[6]. Repeating the 1989 population based Victorian study in 1993, with a sample of 1336 women assessed 6-7 months after birth, the population prevalence of depression was found to be 16.9% (95% confidence interval 14.9 to 18.9%)(S Brown pers comm). Thus there is consistent recent evidence in Australia that this disorder is a major health problem in terms of prevalence.

The issue has implications which extend beyond the affected women themselves to other family members and there is substantial evidence of negative effects of maternal depression on child development[7]. One response to this accumulation of local evidence has been the inclusion in the national health Goals and Targets of the goal of reducing the severity and duration of depression in the year after birth[8].

Factors associated with depression

a) There is less agreement about the associations of depression, partly because many studies have been too small to have the power to identify differences between women who are or are not depressed[9]. The Victorian population-based study (n=790) found no significant associations of depression with maternal age, parity, (except for women aged 35 and older having first babies, a group less than 2% of all women giving birth), family income, maternal education, private health insurance, prior reproductive history (except previous caesarean birth), hospital admission during pregnancy, place of birth, birth weight, length of labour, labour pain, or length of postnatal hospital stay. Depression was associated with caesarean birth (both elective and emergency), with forceps delivery, with an increased number of antenatal procedures, with pethidine use, with epidural anaesthesia, and with a high score for intrapartum procedures[1]. Residence in a non-metropolitan region and breast feeding were both associated with lower rates of depression.

(b) A follow-up study of women who had scored as depressed on the Victorian population based survey found that 30% of women who had been depressed at 8 to 9 months were depressed 21 to 30 months after the index birth[10]. Thus, the problem is not a brief, self-limiting one. Only a third of women who had been depressed had sought help for their disorder from any health professional. If they did seek help it was usually from a GP or Maternal and Child Health Nurse (MCHN) and even fewer had sought help from a mental health professional (15%)[10]. Similarly, a study of 100 mother and baby admissions to a mothercraft hospital in Sydney found that although 39% of women were depressed (EPDS > 13) only 3 had been recognised as such on admission[11]. These findings suggest the need for improved identification and case finding of maternal depression in primary care settings, especially by Maternal and Child Health Nurses (MCHNs) and family physicians[12], and also by paediatricians. The findings confirm the statements made in other countries about this being a 'serious and neglected disorder'[13].

Women in the follow up study who had been depressed, reported less practical and emotional support from their partners and saw themselves as having less social support overall than women not depressed 8-9 months after birth. Women in the case group had also experienced more negative life events, had poorer health and were somewhat more likely to have a 'difficult' toddler[14]. Findings in relation to the social context of depression have been inconsistent[9], again due in part to the low power of most studies, but marital disharmony and lack of support are among the most consistent aspects.

Physical morbidity after birth

Serious maternal physical morbidity such as postpartum haemorrhage, eclampsia and third degree tears are well described, but relatively rare. Evidence from a number of descriptive studies has recently drawn attention to more widespread, but under-recognised health problems after childbirth[15,16,17,18,19,20]. The most common problems reported by Victorian women in the 1993 Survey of Recent Mothers were: extreme tiredness (70%), backache (43%), haemorrhoids (25%), problems with sex (26%), relationship difficulties (18%), painful perineum (21%) and mastitis (16%)[21]. Three studies document symptoms persisting up to 12 months postpartum and beyond[18,19,20]. Glazener and her colleagues found that only 50% of the women who reported health problems persisting after leaving hospital said they had sought any treatment, with much lower rates for particular morbidities, eg painful perineum (21%), urinary incontinence (27%)[18]. The significant association between depression and physical health problems in the postnatal period warrants further study and intervention, particularly in light of these recent findings that the prevalence of physical health problems following childbirth is much higher than is often thought.

Interventions

Despite the accumulating evidence that much maternal physical and emotional morbidity goes unrecognised and untreated, the first postnatal year is a time of increased contact with primary health care services. A recent utilisation review

of medical practitioner services based on Medicare data has found that the mean number of visits to GPs by mothers and babies in the six months following birth was 7.7, significantly higher than the 2.64 GP visits per six months expected for all Australians[22]. Few studies have been designed to evaluate the effectiveness of primary care offered in the postnatal period, although several recent papers have concluded there is an urgent need for such research[18,19]. Jane Gunn's current Victorian RCT evaluating a one week postnatal checkup is one notable example.

The advice women in the follow-up study had for other women who might be depressed emphasised the importance of finding someone to talk to and the need to have some timeout for oneself when someone else was responsible for caring for the child(ren)[14]. The effectiveness of the first of these is borne out by a randomised trial of health visitor 'listening therapy' for half an hour a week which reduced the prevalence of depression by one-third over 6 weeks in a Scottish study[23]. Related strategies providing social support, have been the development of 'befriending' schemes to decrease the isolation of new mothers and provide more social contact and support. A controlled evaluation of one such British scheme, Newpin, confirmed marked benefits to mothers themselves in terms of their mental well-being and ability to take charge of their own lives[24]. A "Community Mothers" program in Dublin also found that first-time mothers randomised to receive monthly visits from voluntary experienced mothers from their own community had better self esteem, felt less tired and miserable and had fewer negative feelings about the first twelve months with their babies than mothers assigned to the control group[25]. A similar scheme of support by older mothers, "Mum's Chums", operated on the Mornington Peninsula in Victoria some years ago, though it was never formally evaluated.

Whilst there has as yet been no research on the benefits for mothers' emotional well-being of providing women with periods of time away from caring for children, the provision of childcare is a feature of a number of community support schemes, such as Newpin. Interestingly, one local government authority in rural Victoria responded to a survey of women's views about services for mothers who were depressed by offering all new mothers in the area a free voucher to their occasional childcare centre - a small but important acknowledgment of mothers' needs for timeout. This area requires further research.

The Victorian follow-up study provided evidence that women who are depressed after birth may not view their depression as **postnatal** depression, with a third of women studied not identifying with this description[14]. This has important implications for intervention programs and suggests that programs targetted at "postnatal depression" will not reach a significant minority of women who may be depressed in the postnatal period. Furthermore, women also commented at length about the lack of societal recognition for their situation as mothers and the difficulties this created for them, first in acknowledging that they might have personal needs themselves (for timeout, for support, for care and attention) and second in feeling able to ask for help[26]. The fears expressed by women that admitting to being depressed would lead to their being labelled by health care providers and the community at large as inadequate mothers is also indicative of the lack of support women often felt as mothers[26].

Strategies for reducing depression after childbirth aimed at raising awareness of this common but often neglected problem among primary care providers and at better co-ordinated service provision to support mothers at the community level, have been recommended[12]. There has been no research to date however, to determine the effectiveness of such a community based or mental health promotion approach to the problem. Whilst several small community projects have received funding in the past few years in Victoria (eg in Ballarat, Springvale and Niddrie), these are very short term projects (6-12 months) and do not involve any controlled comparisons which would enable the assessment of the impact of any interventions on the prevalence of depression among recent mothers in these communities. This would also appear to be the case for the Community Depression Project in Western Australia, where the broad community and professional education strategies being employed to raise awareness about depression and its treatment are not subject to any controlled evaluation (L Johnston, pers comm). In each of these projects this constitutes a major problem in assessing the effectiveness of the strategies employed.

Regier at al argue for the importance of community based interventions in the mental health field for a number of reasons[27]. First, there is considerable epidemiological evidence that only a minority of the community seek professional help for the mental health problems they suffer, and that when they do, they turn mostly to professionals in the primary health care sector[e.g.28,29,30]. Second, there is also evidence that mental health problems are under-recognised in primary care even when help is sought[27,28]. Both these phenomena highlight a need for developing processes of community and professional education about mental health problems and their treatment. Third, there has been only limited success in identifying groups 'at risk' for mental health problems, perhaps most especially depression[27], so that attempts to identify "vulnerable" individuals and develop primary prevention programs targetted to particular groups are unlikely to make a major impact on community prevalence. These issues are also pertinent to depression after birth. Few women seek help, and most who do, turn to primary care providers[10]. There is evidence of under-recognition of depression after birth in primary care settings [10,29], and attempts to identify women at risk for depression in the postnatal period have been successful only in relation to identifying a small proportion of the women who do indeed become depressed (eg women with a previous psychiatric history are more likely to experience depression after birth, but they comprise only a small proportion of all women who become depressed).

Strengthening the capacity of primary care providers (GPs and MCHNS in particular) to deal with mental health issues, in this case depression after birth, is likely to be an important strategy in secondary prevention. For example, a training program for health visitors in the UK on the detection, treatment and prevention of depression among recent mothers

led to their greater confidence in dealing with maternal depression, a decrease over time in their need to refer to other agencies and reduced levels of depression in mothers[31]. There is also accumulating evidence that 'empathic' GPs are both more likely to ask questions which lead to the detection of depression in their patients and that people are more likely to report symptoms of depression to GPs who demonstrate a capacity to listen[27,29].

In assessing the often neglected therapeutic role of listening in the history of psychiatry and medicine, the eminent American psychiatrist Stanley Jackson concludes that "listening is central to learning about and coming to understand a sufferer... The healer learns about the sufferer in direct proportion to the quantity and quality of his listening"[32] (p1631). And Shorter argues that "listening is the main kind of informal psychotherapy the family doctor is able to conduct", and that this is critical in supporting patients to cope with psychological distress[33]. Given the evidence indicating that empathic listening skills can be taught[eg31,34], and that people often have a preference for non-pharmacological treatment strategies[35], then increasing the likelihood that recent mothers are cared for by primary care professionals knowledgeable about the benefits of empathic listening and trained in its use, is one universal, relatively simple and low cost strategy to reduce the prevalence of depression. This is not to deny the place of other treatments for some individuals, (clearly a combination of approaches in dealing with mental health problems may often be beneficial[36]), but rather to pay due attention to the therapeutic relationship in primary care settings as a basis for recovery in most cases.

Thus an integrated community approach to secondary prevention of depression and maternal physical morbidity after birth - involving professional education in the primary care sector, combined with strategies of community and social support to mothers and primary health care treatment strategies based on offering women non-directive counselling (a chance to talk and be heard) and better detection of physical morbidity, within a co-ordinated network of services - has a sound basis for implementation and controlled evaluation.

The research plan thus outlines a community-based intervention, involving community rather than individual randomisation, with primary care providers (GPs and MCHNs), community agencies, community psychiatric services and local government to reduce the prevalence of depression and physical morbidity after birth in recent mothers by increasing knowledge of common postnatal problems amongst primary care professionals, increasing the availability and accessibility of 'someone to talk to' and by providing information to mothers about local support for 'timeout'.

The two principal aims of this trial are :

i to decrease the prevalence of depression 6 to 9 months after birth from an expected rate of 16.9% in the communities without a specific intervention program to 13.9% (or less) in the intervention communities

ii to reduce the proportion of women still depressed 18-21 months after the birth from 30% of those who were depressed at six to nine months to 20%.

The objective of the proposed intervention is to increase the availability and accessibility of 'someone to talk to' and 'timeout' for recent mothers through developmental programs with GPs, MCHNs and local government. As such the trial represents a secondary prevention strategy.

Seven matched pairs of local government areas will be selected giving due consideration to socio-demographic characteristics, population size and distribution, geographic location (rural/metro). Randomisation will occur within pairs assigning one LGA to the intervention program, the other to form the control community for comparison purposes.

The intervention will have two components, one directed to primary care, the other to community services (local government and community agencies), with a steering committee locally appointed to co-ordinate the implementation of the intervention.

A. In **primary care** the objectives are:

* increased recognition of depression in mothers of young children at all primary care contacts;

* an active response to the recognition of depression by primary care providers;

explicit offer of time to talk by both MCHNs and GPs;

* increased recognition and treatment of physical problems which are common in the year after birth (eg mastitis, low back pain, sexual problems)[15].

The strategies[37] will include:

* provision of a training program for MCHNs and GPs, comprising information about the prevalence, associated factors and implications of depression after birth and health after childbirth generally, as well as involving skills training in non-directive counselling/active listening;

* the establishment of professional peer support programs for both MCHNs and GPs;

* with the assistance of the steering committees, local government and the general practice divisions, the development of networks between those in different primary care roles (GPs and MCHNs) and with existing self-help groups; the development of links between the primary care network and the local community psychiatric services so that support can be offered to mothers in a context where consultation, liaison and referral are readily available for GPs and MCHNs.

B. The range of activities with **local government and community agencies** where the objectives are to increase the availability and awareness of support and 'timeout' for recent mothers would include:

* an assessment of the availability of relevant services (occasional child care, recreational services, library, information and counselling services, neighbourhood houses, community centres and community health centres, as well as shopping centre facilities), their accessibility and the extent to which they are 'mother and baby friendly';

* the development of an information kit for mothers comprising a listing of local services for mothers and babies, a brochure outlining some of the common difficulties of being a mother and some strategies for dealing with these which other

women have found helpful, an information ("useful tips") sheet for fathers and a booklet of free service vouchers to recent mothers. The latter might for example include session(s) at the local occasional childcare service, free entry at the local swimming pool, a series of relaxation classes at the Maternal and Child Health Centre, etc. (The specific nature of such vouchers would be dependent on what each LGA considered appropriate and feasible.) The kit would be given to all recent mothers by the MCHN during the home visit made soon after hospital discharge;

* the establishment of a mother-to-mother support network based on the principle of non-professional befriending[19]. Such a network could take many forms, depending on the nature of the community, and it is proposed that the steering committee in each intervention community would choose the appropriate model. Various possibilities are likely:

- calling for volunteer older women to provide support to recent mothers via a monthly visiting program ("grandmother" scheme) perhaps co-ordinated through the council's Child and Family Services Department or the local neighbourhood house;

- MCHNs offering to put two women who have babies the same age in touch with each other for mutual support such as occasional babysitting, getting out together etc. ("peer" support model);

- developing a pool of mothers with older children who could "adopt" a mother with a new baby for advice and support ("experienced" mothers model).

Referral of new mothers to such a network might be either self-referral (local advertising of the network once established would be required) or via the MCHN or GP. Again this is likely to depend on the model chosen in each area.

The developmental work in setting up the trial would be considerable. It is envisaged that two of the chief investigators (RS and SB) would spend the first twelve months on protocol development; development of background materials for the professional training programs; liaison with the reference group for the project and with professional bodies; selection and randomisation of LGAs, after seeking statistical and other advice; identification of key people in intervention communities; selection of community field and support staff; and the implementation of the professional training programs; followed by the appointment of steering committees.

It is proposed that the professional training program be implemented as the first stage of the intervention in each community, ensuring that once other elements of the program are put into place, women receive an appropriate response from primary caregivers. It is also important in the process of appointing the steering committees to identify primary care professionals who are enthusiastic about the intervention and willing to encourage and rally their peers. Such individuals are likely to be identified through the initial training programs.

The development of the professional training programs would be undertaken in conjunction with Maternal and Child Health, local government Child and Family Services Departments, the Royal Australian College of General Practitioners, the General Practice Training Program and Divisions of General Practice, with a view to the programs, if successful, becoming self-sustaining through one or more of these bodies. It is proposed that the programs for MCHNs and GPs be developed separately and designed to meet the particular needs of both groups. Two program facilitators (one for MCHNs and one for GPs) will be employed to deliver the programs (envisaged as being one full-day or two half day sessions) in each of the intervention communities. It is estimated that there are likely to be between 10 and 20 MCHNs and between 50 and 80 GPs in each intervention area.

The developmental work necessary in intervention communities is also substantial, beginning with the setting up of a small steering committee of key people in each area. The employment of a community development/field worker is seen as essential to facilitate the establishment of the intervention in each LGA by:

* liaising with local government and non-government agencies, GPs, Maternal and Child Health Nurses and local psychiatric services

* assessing levels of community service provision and compiling information on services for mothers, soliciting voucher contributions from relevant bodies, and producing the package of information for mothers,

* assisting in the establishment of the mother-to mother support network through local government or a community agency as appropriate

* providing support to the steering committee in overseeing the intervention (in the initial 12-18 month establishment phase) and the integration of the community service program once established, within ongoing service provision by local government or community agencies.

Although the major elements of the local government and community agency intervention arm would be defined for each intervention community, (eg information kit to mothers, voucher scheme, mother-to-mother support network) some flexibility needs to be maintained which would allow each steering committee to make decisions about other supportive interventions as well as the appropriate implementation of the different elements of the intervention. For example some steering committees may feel that organising a community forum to raise awareness about the issue is appropriate, others may want to publicise the program in the local papers; and the nature of mother-to mother support programs is likely to vary as outlined above.

It is proposed that a regular newsletter be produced by the Research Team keeping the Steering Committees in touch with the development of the programs in each LGA, thus enabling the sharing of "good ideas", common difficulties and creative solutions to practical problems in establishing the programs.

Design, outcome measurement and sample size issues

As stated above, the current plan is to stratify communities (local government areas) into pairs, with some matching on key social and community factors and to randomise within the pairs. This matching would take into account socio-

demographic factors shown to have an association with depression, such as proportions of single mothers, mothers of NESB, mothers having an operative delivery, mothers over 34 having a first baby. Numbers of births, geographic size, and levels of community activity (generally on health and social issues and any previous activity around the issue of maternal depression)[36], would also be taken into account in matching LGAs.

Process evaluation to ensure that the intervention program is functioning adequately before impacts and outcomes are measured[38] would be undertaken in each intervention community via postal questionnaires to a random sample of 100 mothers and all MCHNs and GPs. This would occur at two time points during the trial (12-18 months after initial establishment and then again 12-18 months later) to assess whether all elements of the intervention are in operation, and continue to function over time. At the second time point a further workshop would also be offered to all MCHNs and GPs enabling them to review the implications of the previous training on their practice since. Another component of the process evaluation would involve interviews with steering committee members and other key informants in each of the intervention communities (See Box 1 at end of Research Plan for more details.)

Assessment of depression and of general health would be made using the EPDS and the SF-36[39] mailed out to mothers 6 months after birth. A reminder postcard would be mailed out two weeks later, a strategy that is likely to achieve a response rate of around 67%. All women scoring as depressed on the 6 month EPDS as well as a random sample of those scoring as not depressed would also be sent an EPDS and an SF-36 two years after birth. The inclusion of a random sample of mothers not depressed would ensure confidentiality regarding women's depression status as the mailout would occur using local government birth registration record numbers identified on the 6 month EPDS.

The sample size to detect the first stated aim, namely a depression prevalence reduction of 3% ($\alpha=0.05$, 2-sided, $\beta=0.20$), given individual randomisation, is 2160 in each group. However, the sample size given community (or cluster) randomisation is much greater[40], in the order of 4,800 in both the intervention and control communities. This number is obtained using power calculations[40], information on the number of births in LGAs in 1994/5 (Maternal and Child Health Annual Data Collection - unpublished data), depression prevalence data[1] and a cost analysis using the budget estimates of community and individual costs. It has been assumed that seven matched pairs of clusters, including two rural pairs, gives the maximum power most economically. The expected number responding from each cluster ranges from 500 to 900 and has a geometric mean of approximately 700. The response rate is estimated to be about 67% and a 12 month data collection period is likely to be sufficient to achieve the sample size required taking this loss to follow up into account. These calculations have been corroborated using another power calculation (ref Schesselman J, Elbourne D - pers comm) which enabled an estimate of the size of the correlation within randomisation pairs to be made and highlighted that 80% power can only be achieved if the pairs are highly correlated (>0.9) with respect to pre-intervention depression prevalence.

A complication in relation to the estimation of sample size is that if the strategies outlined above are successful their effectiveness is likely to increase slowly and be cumulative, so that an appropriate analysis would compare changes over time in the intervention and control communities[41]. There are approximately 30 LGA pairs which would appropriately form the basis for selection into the trial, (given similar numbers of births, proportion of single mothers, women of NESB, older primiparas, similar rates of operative delivery, etc.).

Data Management and Analysis

The process of data collection is quite complex given the necessity to mail out instruments and a reminder postcard two weeks later, to all mothers in both intervention and control communities 6 months after birth, on a rolling basis over twelve months; and then to send the instruments out to a sub-group of mothers two years after the birth, again on a rolling basis. To ensure confidentiality, mailouts will occur from city or shire offices using birth registration information, necessitating the setting up of appropriate data systems compatible with the local computerised systems in each LGA.

The returned questionnaires and instruments will be coded and entered at the Centre into a database (for instance EpiInfo or dBase) by the MRA in batches shortly after receipt of the questionnaire.

A data monitoring committee will be established to monitor safety and efficacy, with interim analyses as appropriate: Dr Janet Hiller (Chair), Department of Community Medicine, University of Adelaide and Dr John Carlin, Clinical Epidemiology and Biostatistics Unit, Department of Paediatrics, University of Melbourne, Royal Children's Hospital have agreed to participate.

At the end of the (first) data collection (1/1/2001) descriptive analysis of the characteristics of the mothers in the clusters will be made. Primary assessment of the overall effect of the intervention with respect to depression will be made using split-plot analysis of variance (adapted to the binary outcome) to take account of the cluster sampling process used[42]. Trends in depression rates over the 12 months will be assessed[43]. Analysis of the SF-36 will be made using similar techniques. Current statistical packages likely to be used include EpiInfo, SPSS and EGRET.

It is acknowledged that there is much ongoing development and debate about the appropriate methods of analysis for cluster randomised trials[44,45,46] and the research team will continue to monitor these developments for the purposes of refining the proposed data analysis for PRISM.

Timelines: **1997** protocol development, including the questions discussed above, identification of possible communities and key influential people; description of process measures, randomisation; appointment of support staff, data management systems designed, development of the professional training programs in intervention communities **1998** implementation of professional training programs, appointment of field staff, developmental work and establishment of the intervention program within the intervention communities, negotiation of depression measurement in control communities **1999** maintenance of the developmental work and implementation, process evaluation, data collection and data entry begin, beginning of impact and outcome evaluation by mid 1999, **2000-01** programs maintained, data collection for impact and outcome evaluations continues

2002 data collection complete by mid-year, data analysis and project written up by end of 2002. (See also attached timeline chart for clarity.)

Reference Group

Consultation about the aims and nature of the project has already occurred and a reference group has been formed comprising people with a range of interests and expertise to support the research:

Prof Helen Herrman, Director of Psychiatry, St Vincents Hospital, Professor of Psychiatry, University of Melbourne

Prof Roger Strasser, Director, Centre for Rural Health, Monash University

Ms Jan O'Connell, A/Manager, Community Child Health, (with responsibility for Maternal and Child Health), Primary Care Branch, Department of Health and Community Services Victoria

Ms Rae Presser, Maternal and Child Health Nurse, City of Yarra

Dr Regina Clarke, General Practitioner, Bendigo Community Health Service

Ms Chris Fawcett, Consumer advocate, mother of three children, with a community development background

Ms Christina Bryant, Clinical Psychologist, Mother and Baby Unit, Austin Repatriation Medical Centre, Larundel Campus

Dr Andrew Stocky, Director, Mother Baby Unit, Monash Medical Centre, Director Mother Baby Program, Melbourne Clinic

Ms Jenny Alter, Senior Project Officer, Client Services Unit, Psychiatric Services Branch, Department of Health and Community Services Victoria.

Dr David Legge, Senior Lecturer, Health Systems Sciences, La Trobe University

Victorian Municipal Community Services Association - a representative to be nominated

Assoc Prof Michael Hamel-Green - Co-ordinator BA (Community Development), Department of Social and Cultural Studies, Victoria University

Box 1: Process and Impact Evaluation

There is clearly a need for detailed process and impact evaluation to assess each intervention community's exposure to the intervention program and its immediate impact before outcome evaluation occurs. The central questions which need to be addressed in process and impact evaluation will be the focus of developmental work in the first twelve months of the trial. A preliminary overview of the sorts of monitoring which will be required include, in relation to each aspect of the intervention:

Professional education workshops:

Have the workshops consistently covered the content in the way intended? Is this true for all LGAs? (independent participant assessment)

Have the workshop personnel completed their tasks satisfactorily? (style, delivery, facilitating discussion - independent participant assessment)

Have the workshops been attended by the majority of primary health care professionals in the area? (attendance records compared to total numbers of GPs and MCHNs)

How was this program received/evaluated by them? (evaluation sheets completed at the end of all sessions)

Information material for mothers:

What responses do recent mothers have to the information kit? (focus group discussions with recent mothers in pilot/development phase; questions included in a survey of a random sample of 100 mothers in each intervention LGA during proces evaluation phase)

Are MCHNs happy to hand it out? (telephone survey of MCHNs)

Are all recent mothers receiving the information kit? (questions asked in mothers' survey)

Has there been sufficient local interest to generate at least 20 service vouchers for mothers? (documentation of contributors to voucher booklet in each LGA)

Is there evidence that women are using these "timeout" vouchers? (service providers to be asked to keep presented vouchers, telephone check of a sample of service providers in each LGA)

Primary care offers of "time to talk":

Are mothers being encouraged to talk about emotional health issues by MCHNs and GPs? (questions asked in mothers' survey)

Do mothers find this helpful? (questions asked in mothers' survey)

Do primary care professionals feel comfortable with this role? (telephone interviews with MCHNs and GPs)

Is there any evidence of networking between MCHNs and GPs and with specialist mental health services, concerning recent mothers? (telephone interviews with MCHNs, GPs and mental health services)

Local community support for the project:

Has there been a satisfactory level of local commitment to the project? (Steering Committee- regular meetings? good attendances? level of participation of members?/Council Community Services Department: council officer support for trial?/local GP Division: documentation of the role and activities of all of these in establishing the intervention in each LGA /voluntary agencies involvement) **Has the project received appropriate local publicity?** (Records kept of all publicity/media work around the trial in each LGA) **Has a non-professional befriending network been set up? How does this function? Who are the befrienders/ recipients?** (data collection on numbers of volunteers registering with network and mothers referred for "befriending"; focus group discussions with volunteers and recipients)

PRISM TIMELINES

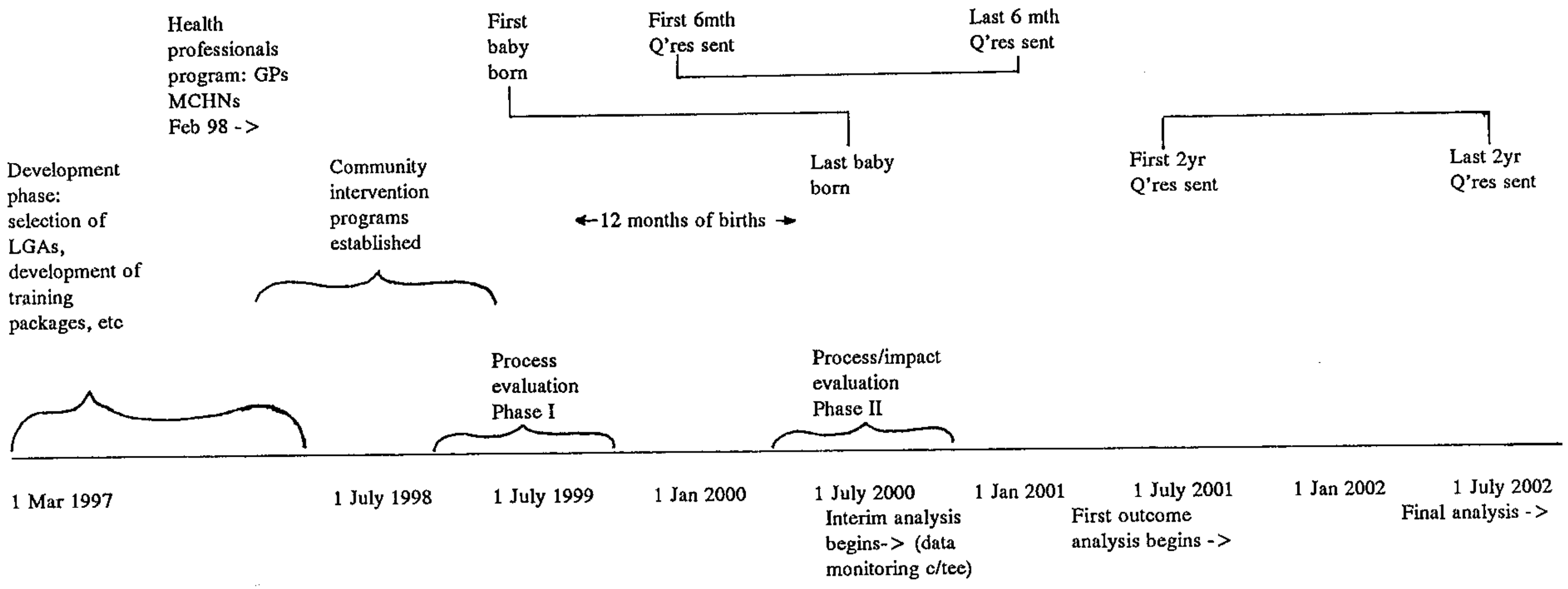

13e

17 (a) References Cited For This Project

(One page maximum)

1 Astbury J, Brown S, Lumley J, Small R. Birth events, birth experiences and social factors in depression after birth Aust J Public Health 1994;18:176-84.

2 Jayasuriya R et al. Measuring maternity services: quality and outcome. Illawarra Area Health Service, April 1993.

3 Cox JL et al. Detection of postnatal depression: development of the 10-item Edinburgh Postnatal Depression Scale. Br J Psychiatry 1987; 150: 782-6.

4 Harris, B et al. The use of rating scales to identify postnatal depression. Br J Psychiatry 1989; 154: 813-817.

5 Murray L, Carothers AD. The validation of the Edinburgh Postnatal Depression Scale on a community sample. Br J Psychiatry 1990; 157: 288-290

6 Dennerstein L et al. Postpartum depression: risk factors. J Psychosomat Obstet Gynaecol 1989; 10 (supplt): 53-656

7 Cox, A. Maternal depression and impact on children's development. Arch Dis Childh 1988; 63: 90-95

8 Nutbeam D et al Goals and targets for Australia's health in the year 2000 and beyond. Canberra: AGPS, 1993.

9 Romito P. Unhappiness after childbirth. In: Chalmers I, Enkin M, Keirse M (eds). Effective care in pregnancy and childbirth. Oxford: Oxford University Press, 1989: 1433-1466

10 Small R, Brown S, Lumley J, Astbury J. Missing voices: what women say and do about depression after childbirth J Reprod Inf Psychol 1994;12:89-103

11 Barnett B et al. Mood disorders among mothers of infants admitted to a mothercraft hospital. J Paed Child Health 1993;29:

12 Lloyd D, Redman S. Postnatal depression: prevalence, detection and usual care in a primary care setting. Proceedings of the 10th International Congress of Psychosomatic Obstetrics and Gynaecology, Stockholm, June 1992, Abstract F460, p151

13 Cox JL. Postnatal depression: a serious and neglected postpartum complication. Baillieres Clin Obstet Gynaecol 1989; 3:839-55

14 Small R, Astbury J, Brown S, Lumley J. Depression after childbirth: does social context matter? Med J Aust 1994;161:473-7

15 MacArthur C et al. Health after childbirth: an investigation of long term health problems beginning after childbirth in 11,701 women. London : HMSO, 1991

16 Glazener CMA, MacArthur C, Garcia J. Postnatal care: time for a change. Contemp Rev Obstet Gynaecol 1993;5:130-6

17 Bick D, MacArthur C. The extent, severity and effect of health problems after childbirth. Br J Midwifery 1995;3:27-31

18 Glazener C, Abdella M, Stroud P, Naji S, Templeton A, Russell I. Postnatal maternal morbidity: extent, causes, prevention and treatment. Br J Obstet Gynaecol 1995;102:282-7

19 Gjerdingen D, Froberg D, Chaloner K, McGovern P. Changes in women's physical health during the first postpartum year. Arch Fam Med 1993;2:277-83

20 Johansen R, Wilkinson P, Bastible A, Ryan S, Murphy H, O'Brien S. Health after childbirth: a comparison of normal and assisted vaginal delivery. Midwifery 1993;9:161-8

21 Brown S, Lumley J. Mothers' health after childbirth: the first nine months. Centre for the Study of Mothers' and Children's Health Conference, When research points to changing practice: what then? Melbourne, October 1995, 26.

22 Gunn J, Lumley J, Young D. Visits to medical practitioners in the first six months of life. J Paed Child Health (in press)

23 Holden JM et al. Counselling in a general practice setting: controlled study of health visitor intervention in the treatment of postnatal depression. Br J Psychiatry 1989; 298:223-624

24 Cox AD et al. Evaluation of a home visiting and befriending scheme: Newpin. J R Soc Med 1991; 84: 217-220

25 Johnson Z, Howell F, Molloy B. Community mothers' programme: randomised controlled trial of non-professioonal intervention in parenting. BMJ 1993;306:1449-52

26 Brown S, Lumley J, Small R, Astbury J. Missing voices: The experience of motherhood. Melbourne: OUP, 1994.

27 Regier DA, Hirschfeld RMA, Goodwin FK, Burke JD, Lazar JB, Judd LL. The NIMH depression awareness, recognition and treatment program: structure, aims and scientific basis. Am J Psychiatry 1988;145:1351-57

28 Cooper B, Eastwood R (eds). Primary health care and psychiatric epidemiology. London: Tavistock, 1992

29 Romans-Clarkson SE, Walton VA, Dons DJ, Mullen PE. Which women seek help for their psychiatric problems? NZ Med J 1990;103:445-48

30 Jenkins R, Newton J, Young R. The prevention of depression and anxiety: the role of the primary care team. London: HMSO, 1992

31 Gerrard J, Holden JM, Elliott SA, McKenzie P, McKenzie J, Cox JL. A trainer's perspective of an innovative programme teaching health visitors about the detection, treatment and prevention of postnatal depression. J Adv Nursing 1993;18:1825-32

32 Jackson SW. The listening healer in the history of psychological healing. Am J Psychiatry 1992;149:1623-32

33 Shorter E. Bedside manners: the troubled history of doctors and patients. Harmondsworth: Viking, 1986

34 Gask L. Teaching psychiatric interviewing skills to general practitioners. In: Jenkins R, Newton J, Young R (eds). The prevention of depression and anxiety: the role of the primary care team. London: HMSO, 1992, 39-45

35 Paykel ES. Pharmacological and psychotherapeutic approaches to the treament of affective disorders. In Mendlewicz J, Brunello N Langer SG, Racagni G, (eds). International Academy for Biomedical and Drug Research, vol 5, Basel:Karger, 1993:62-74

36 Eastwood R, Schneiderman G. Couch fellows. Lancet 1994;343:131-2

37 Elliott SA. Psychological strategies in the prevention and treatment of postnatal depression. Baillieres Clin Obstet Gynaecol 1989;3:879 903

38 Hawe P, Degeling D, Hall J. Evaluating health promotion: a health worker's guide. Sydney: MacLennan and Petty, 1990

39 Jenkinson C, Coulter A, Wright L. Short form 36 (SF36) health survey questionnaire: normative data for adults of working age. Br Med J 1993;306:1437-40

40 Shipley MJ, Smith PG, Dramaix M. Calculation of power for matched pair studies when randomization is by group. Int J Epidemiol 1989;18:457-61

41 Salonen JT, Kottke TE, Jacobs DR, Hannan PJ. Analysis of community-based cardiovascular disease prevention studies-evaluation issues in the North Karelia project and the Minnesota Heart Health Program. Int J Epidemiol 1986;15:176-182.

42 Maritz JS, Jarrett, RG. The use of statistics to examine the association between fluoride in drinking water and cancer death rates. Appl Statist 1983;32:97-101

43 Zeger SL, Liang K-Y. Longitudinal data analysis for discrete and continuous outcomes. Biometrics 1986;42:121-30.

44 Donner A, Klar N Methods for comparing event rates in intervention studies when the unit of analysis is acluster. Am J Epidemiol 1994;140:270-89

45 Murray DM, McKinlay SM, Donner A, Dwyer JH, Raudenbush SW, Graubard BI. Design and analysis issues in community trials. Evaluation Rev 1994;18:493-514

46 Koepsall TD, Diehr PH, Cheadle A, Kristal A. Invited commentary: Symposium on community intervention trials. Am J Epidemiol 1994;142:594-9

17 (b) Publications of Chief Investigators for Track Record Consideration:

Please ensure that you list only those papers that have been published, are in press or for which you have received a final acceptance from the journal's editor. (Show date of acceptance) Documentary evidence of all final acceptances must be available to Regional Grant Interviewing Committees. Do not include abstracts. A maximum of 6 publications per Chief Investigator considered to best reflect research contributions to date may be asterisked (*).

Publications 1991-5 - Judith Lumley

46 McLachlan Z, Milne J, Walker B, Lumley J. A randomized trial of ultrasound treatment for post-partum breast engorgement. Aust J Physiother 1991; 37: 23-28.

48 Lumley J. Preventing and managing prematurity. **Int J Technol Assessment in Health Care** 1991; 7: 460-477.

49 Bell R, Lumley J. Low birthweight and socio-economic status in Victoria. **Aust J Public Health** 1992; 16: 15-19.

51 The Victorian Infant Collaborative Study Group (VICS). Regional impact of improved perinatal care for infants of birthweight 500-999g. **Arch Dis Childh** 1991;66: 765-769.

52 Biro M-A, Lumley J. The safety of team midwifery: a decade of care at the Monash Birth Centre. Med J Aust 1991; 155: 478-480.

53 Ponsonby A-L, Jones MEJ, Dwyer T, Gilbert N, Lumley J. An investigation of the contribution of climatic temperature to regional variations in SIDS incidence in the Australian States. **Med J Aust** 1992; 156: 246-251.

54 Ponsonby A-L, Jones MEJ, Lumley J, Dwyer T, Gilbert N. SIDS: an investigation of contributing factors to the incidence difference between Victoria and Tasmania. **Med J Aust** 1992; 156: 252-254.

55 Lumley J. Stopping smoking - again. **Br J Obstet Gynaecol** 1991; 98: 847-852.

56 Bell R, Lumley J. Antenatal uterine activity monitoring in women at high risk of preterm labour. **Eur J Obstet Gynecol Rep Biol** 1992;46:65-72.

* 57 Halliday JL, Lumley J, Sheffield LJ, Robinson HP, Renou P, Carlin JB. Importance of complete follow-up of spontaneous fetal loss after amniocentesis and chorion villus sampling. **Lancet** 1992; 340: 886-90.

58 Small R, Brown S, Lumley J. To stay or not to stay: are fears about shorter postnatal hospital stays justified? **Midwifery** 1992; 8: 170-177.

59 Bower C, Raymond M, Lumley J, Bury G. Trends in neural tube defects in three Australian states. Med J Aust 1993; 158: 152-154.

60 Brown S, Lumley J. Antenatal care: a case of the inverse law? **Aust J Public Health** 1993; 17: 95-102.

61 Halliday J, Lumley J, Sheffield LJ, Lancaster PAL. Limb deficiency defects and their association with chorion villus sampling. Am J Med Genet 1993; 47: 1096-1098.

17 (b) Publications of Chief Investigators for Track Record Consideration:

Please ensure that you list only those papers that have been published, are in press or for which you have received a final acceptance from the journal's editor. (Show date of acceptance) Documentary evidence of all final acceptances must be available to Regional Grant Interviewing Committees. Do not include abstracts. A maximum of 6 publications per Chief Investigator considered to best reflect research contributions to date may be asterisked (*).

Number *

62 Jonas H, Lumley J. Triplets and quadruplets born in Victoria between 1982 and 1990: impact of IVF and GIFT on rising birth rates. **Med J Aust** 1993; 158: 695-663.

* 63 Kilkenny M, Lumley J. Ethnic differences in the incidence of the sudden infant death syndrome in Victoria, Australia 1985-89. **Paed Perinat Epidemiol** 1994; 8: 27-40.

64 Lumley J. Unhappy babies, unhappy mothers (Annotation). **J Paed Child Health** 1993; 29: 264-5.

* 65 Lumley J. The epidemiology of preterm birth. **Bailliere's Clin Obstet Gynaecol** 1993; 7: 477-498.

66 Lumley J, Bakoula C. Perinatal mortality in Greece and in Greek-born women in Victoria. What does a 'natural experiment' suggest? **Eur J Obstet Gynaecol Repr Biol** 1993; 50: 65-70.

67 Lumley J, Brown S. Attenders and non-attenders at childbirth preparation classes in Australia: how do they and their births differ? **Birth** 1993; 20: 123-129.

68 Venn A, Lumley J. Births after a period of infertility in Victorian women 1982-1990. **Aust NZ J Obstet Gynaecol** 1993; 33: 379-384.

69 Victorian Infant Collaborative Studies Group (VICS). Cost of improving the outcome for infants of birthweights under 1000g in Victoria. **J Paed Child Health** 1993; 29: 56-62.

70 Victorian Infant Collaborative Studies Group (VICS). Improving the quality of survival for infants of birthweight < 1000g born in non-Level III centres in Victoria. **Med J Aust** 1993; 158: 24-27.

71 Brown S, Lumley J. Satisfaction with labor and birth: a survey of 790 Australian women. **Birth** 1994; 21: 4-13.

72 Venn A, Lumley J. Clomiphene citrate and pregnancy outcome - a review. **Aust NZ J Obstet Gynaecol** 1994; 34: 56-66.

73 Rice P, Ly B, Lumley J. Childbirth and soul loss: the case of a Hmong woman. **Med J Aust** 1994; 160: 577-578.

74 Astbury J, Brown S, Lumley J, Small R. Birth events, birth experiences, social factors and depression after birth. **Aust J Public Health** 1994; 18: 176-184.

75 Halliday JL, Lumley J, Bankier A. Karyotypic abnormalities in fetuses diagnosed as abnormal on ultrasound before 20 weeks gestational age. **Prenatal Diagnosis** 1994; 14: 689-697.

17 (b) Publications of Chief Investigators for Track Record Consideration:

Please ensure that you list only those papers that have been published, are in press or for which you have received a final acceptance from the journal's editor. (Show date of acceptance) Documentary evidence of all final acceptances must be available to Regional Grant Interviewing Committees. Do not include abstracts. A maximum of 6 publications per Chief Investigator considered to best reflect research contributions to date may be asterisked (*).

76 Small R, Astbury J, Brown S, Lumley J. Depression after childbirth: the social context. **Med J Aust** 1994; 161: 473-477.

77 Small R, Brown S, Lumley J, Astbury J. Missing voices: what women say and do about depression after birth. J Reprod Infant Psychol 1994; 12: 89-103.

78 Bell R, Palma S. Lumley J. The effect of vigorous exercise in pregnancy on fetal outcome. **Aust NZ J Obstet Gynaecol** 1995; 35: 46-51

* 79 Halliday J, Lumley J, Watson L. Comparison of women who do and do not have amniocentesis and chorion villus sampling in Victoria. Lancet 1995; 345: 704-709

80 Yates J, Lumley J, Bettio J, Bell R. Method for cohort and case control studies: the prevalence, timing and effectiveness of obstetric ultrasound. **Paed Perinatal Epidemiol.** 1995; 9: 225-240.

* 81 Halliday J, Watson L, Lumley J, Sheffield L, Danks D. New estimates of Down syndrome risks at chorion villus sampling, amniocentesis and livebirth in women of advanced maternal age from a uniquely defined population. **Prenatal Diagnosis** 1995; 15: 455-465

82 Kilkenny M, Riley M, Lumley J. Follow-up validation study of the Victorian Congenital Malformations Register. J Paed Child Health 1995; 31; 323-325

83 Victorian Infant Collaborative Study Group (VICS). Outcome to five years of age of children 24 to 26 weeks gestational age born in the State of Victoria. Med J **Aust** 1995; 163: 11-14.

* 84 **Venn A,** Watson L, Lumley J, Giles G, King C, Healy D. Incidence of breast and ovarian cancer after infertility and *in vitro* fertilization Lancet 1995; 346: 995-1000

85 Yates J, Lumley J, Bell R. The extent and timing of obstetric ultrasound utilisation in Victoria 1991-2: a population-based study. **Aust NZ J Obstet Gynaecol** 1995; 35: 375-379

86 Lumley J. Monitoring systems to evaluate the quality of perinatal care. **Sozial- und Präventivmedizin** 1995: 40: 337-402

Papers in press:

Lumley J, Bastian H. Competing or complementary? Ethical considerations and the quality of randomized trials. Int J Technol Assess in Health Care.

Potter A, Lumley J. New risk factors: do they explain ethnic and place of birth differences in SIDS incidence in Victoria. Early Human Development

Gunn J, Lumley J, Young D. Visits to medical practitioners in the first six months of life. J Paed Child Health

17 (b) Publications of Chief Investigators for Track Record Consideration:

Please ensure that you list only those papers that have been published, are in press or for which you have received a final acceptance from the journal's editor. (Show date of acceptance) Documentary evidence of all final acceptances must be available to Regional Grant Interviewing Committees. Do not include abstracts. A maximum of 6 publications per Chief Investigator considered to best reflect research contributions to date may be asterisked (*).

Number *

Publications 1990-96 - Stephanie Brown

1. Refereed journals

1. Small R, Lumley J, Brown S. To stay or not to stay: Victorian women's experiences of length of hospital stay after birth, *Midwifery,* 1992;8; 170-177.

2.* Brown S, Lumley J. Antenatal care: a case of the inverse care law? *Aust J Public Health*, 1993; 17(2); 95-103.

3. Lumley J, Brown S. Attenders and non-attenders at childbirth preparation classes in Australia: how do they and their births differ? *Birth*, 1993; 20(3); 123-130.

4.* Brown S, Lumley J. Satisfaction with labor and birth: an Australian survey *Birth* 1994, 21, 4-13.

5. Astbury J, Brown S, Lumley J, Small R. Birth events, birth experiences and social differences in depression after childbirth. *Aust J Public Health,* 1994, 18, 176-184.

6. Small R, Astbury J, Brown S, Lumley J. Depression after childbirth: does social context matter? *Med J Aust*, 1994;161.

7. Small R, Brown S, Lumley J, Astbury J. Missing voices: what women say and do about depression after childbirth. *Journal of Infant and Reproductive Psychology* 1994;12;89-103.

2. Books and reports

1. Brown S, Lumley J. *Consumer Views of Childbirth Services: A Survey for Recent Mothers*, Centre for the Study of Mothers' and Children's Health, Monash University, 1991, pp47, [24 individual hospital reports].

2. Brown S, Lumley J, Small R, Astbury J. *Missing Voices. The Experience of Motherhood* Oxford University Press, Melbourne, 1994.

3.* Brown S, Lumley J, Small R. *Reasons to stay, reasons to go: Victorian women talk about early discharge*, Centre for the Study of Mothers' and Children's Health, Melbourne, 1995, pp72.

17 (b) Publications of Chief Investigators for Track Record Consideration:

Please ensure that you list only those papers that have been published, are in press or for which you have received a final acceptance from the journal's editor. (Show date of acceptance) Documentary evidence of all final acceptances must be available to Regional Grant Interviewing Committees. Do not include abstracts. A maximum of 6 publications per Chief Investigator considered to best reflect research contributions to date may be asterisked (*).

Number *

Publications - Rhonda Small

A. Refereed journals

1.* Small R, Lumley J, Brown S. To stay or not to stay: Are fears about shorter postnatal hospital stays justified? *Midwifery*, 1992;8:170-7.

2. Astbury J, Brown S, Lumley J, Small R. Birth events, birth experiences, social differences and depression after birth. *Aust J Public Health*, 1994;18:176-84.

3.* Small R, Astbury J, Brown S, Lumley J. Depression after childbirth: does social context matter? *Med J Aust* 1994;161:473-7.

4.* Small R, Brown S, Lumley J and Astbury J. Missing voices: what women say and do about depression after birth. *J Reprod Infant Psychol* 1994;12:89-103.

B. Reports/Books

1. Lumley J, Small R, Yelland J. *Having a baby in Victoria: Final Report of the Ministerial Review of Birthing Services in Victoria*. Melbourne: Health Department Victoria, 1990.

2. Brown S, Lumley J, Small R, Astbury J. *Missing voices: the experience of motherhood*. Melbourne: Oxford University Press, 1994.

3. Brown S, Lumley J, Small R. *Reasons to stay, reasons to go: Victorian women talk about early discharge,* Centre for the Study of Mothers' and Children's Health, Melbourne, 1995, pp72.

Publications: **Lyndsey Watson**

1 Kune GA, Kune S, Watson LF. The role of heredity in the etiology of large bowel cancer. data from the Melbourne Colorectal Cancer Study. **World J Surg** 13:124-131, 1989.

2* Kune GA, Kune S, Watson LF. Children, Age at first birth, and colorectal cancer. data from the Melbourne Colorectal Cancer Study. **Am J Epidemiol** 129:533-542, 1989.

3 Kune GA, Kune S, Watson LF, Pierce R, Field B, Vitetta L, Merenstein D, Hayes A, Irving L. Serum levels of beta carotene, vitamin a, and zinc in male lung cancer cases and controls. **Nutr Cancer** 12:169-176, 1989.

17 (b) Publications of Chief Investigators for Track Record Consideration:

Please ensure that you list only those papers that have been published, are in press or for which you have received a final acceptance from the journal's editor. (Show date of acceptance) Documentary evidence of all final acceptances must be available to Regional Grant Interviewing Committees. Do not include abstracts. A maximum of 6 publications per Chief Investigator considered to best reflect research contributions to date may be asterisked (*).

Number *

4 Pierce RH, Kune GA, Kune S, Watson LF, Field B, Merenstein D, Hayes A, Irving LB. Dietary and alcohol intake, smoking pattern, occupational risk and family history in lung cancer patients: results of a case-control study in males. **Nutr Cancer** 12:237-248, 1989.

5 Kune GA, Kune S, Watson LF. Dietary sodium and potassium intake and colorectal cancer risk. **Nutr Cancer** 12:351-359, 1989.

6 Kune GA, Kune S, Watson LF. Body weight and physical activity as predictors of colorectal cancer risk. **Nutr Cancer** 13:9-17, 1990.

7 Kune GA, Kune S, Watson LF. Oral contraceptive use does not protect against large bowel cancer. **Contraception** 41:19-24, 1990.

8* Kune GA, Kune S, Field B, White R, Brough W, Schellenberger R, Watson LF. Survival in patients with large bowel cancer. A population based investigation from the Melbourne Colorectal Cancer Study. **Diseases of the Colon and Rectum** 33: 938-946, 1990.

9 Kune GA, Kune S, Watson LF, Bahnson CB. Personality as a risk factor in large bowel cancer. Data from the Melbourne Colorectal Cancer Study. **Psych Med** 21: 29-41, 1991

10 Kune GA, Kune S, Watson LF, Rahe R. Recent life change and large bowel cancer. Data from the Melbourne Colorectal Cancer Study. **J Clin Epidemiol** 44: 57-68, 1991.

11 Kune GA, Kune S, Read A, MacGowan K, Penfold C, Watson LF. Colorectal polyps, diet, alcohol and family history of colorectal cancer: A case control study. **Nutr Cancer** 16: 25-30, 1991.

12* Becker NG, Watson LF, Carlin JB. A method of non-parametric back-projection and its application to AIDS data. **Statistics in Medicine** 10: 1527-1542, 1991.

13 Kune GA, Kune S, Vitetta L, Watson LF. Smoking and colorectal cancer risk: Data from the Melbourne Colorectal Cancer Study and brief review of literature. **Int J Cancer** 50: 1-4, 1992.

14 Kune GA, Kune S, Watson LF. The effect of family history of cancer, religion, parity and migrant status on survival in colorectal cancer. **Eur J Cancer** 28a, 8/9: 1484-1487, 1992.

15 Kune GA, Bannerman S, Watson LF. Attributable risk for diet, alcohol and family history in the Melbourne Colorectal Cancer Study. **Nutr Cancer** 18: 231-235, 1992.

16 Kune GA, Bannerman S, Field B, Watson LF, Cleland H, Merenstein D, Vitetta L. Diet, alcohol, smoking, serum beta-carotene, and vitamin A in male melanocytic cancer patients and controls. **Nutr Cancer** 18:237-244, 1992.

17 Kune GA, Kune S, Field B, Watson LF, Cleland H, Merenstein D, Vitetta L. Oral and pharyngeal cancer, diet, smoking, alcohol and serum vitamin A and β-carotene levels: A case-control study in men. **Nutr Cancer** 20: 61-70, 1993.

17 (b) Publications of Chief Investigators for Track Record Consideration:

Please ensure that you list only those papers that have been published, are in press or for which you have received a final acceptance from the journal's editor. (Show date of acceptance) Documentary evidence of all final acceptances must be available to Regional Grant Interviewing Committees. Do not include abstracts. A maximum of 6 publications per Chief Investigator considered to best reflect research contributions to date may be asterisked (*).

Number *

18* Becker NG, Watson LF, Marschner IC, Motika M, Carlin JB. Assesing the extent of the Australian HIV epidemic from AIDS surveillance data. **Aust J Public Health** 17: 226-31, 1993.

19 Kune GA, Kune S, Watson LF. Perceived religiousness is protective for colorectal cancer. Data from the Melbourne Colorectal Cancer Study. **J Roy Soc Med** 86: 645-7, 1993.

20 Marschner IC, Watson LF. An Improved EMS Algorithm for back-projection of AIDS incidence data. **J Stat Computation and Simulation** 50: 1-20, 1994.

21 Halliday J, Lumley J, Watson L. Comparison of women who do and do not have amniocentesis and chorion villus sampling. Lancet 1995; 345: 704-709.

22 Halliday JL, Watson LF, Lumley J, Danks DM, Sheffield LJ. New estimates of Down syndrome risks at chorion villus sampling, amniocentesis and livebirth in women of advanced maternal age from a uniquely defined population. Prenatal Diagnosis 1995; 15: 455-465.

23 Cooper R, Potter A, Watson L, Yelland J. Co-sleeping in infancy. (Letter) J Paed & Ch Health 1995;31:60-61.

24 Parsons CDF, Spicer MJ, Richardson M, Peterson C, Watson LF. Infection control and HIV/AIDS: Perception of risks among nurses and hospital domestic workers. Aust J Public Health 1995;19:492-500.

25 Venn A, Watson L, Lumley J, Giles G, King C, Healy D. Breast and ovarian cancer incidence after infertility and in vitro fertilisation. Lancet 1995;346:995-1000.

26 Venn A, Watson L, Lumley J, Giles G, King C, Healy D. Authors reply. Lancet 1995;346:1627-8.

In Press

Bonawit VL, Watson L. Nurses who work in General Practitioners' rooms: A Victorian survey. AJAN.

Potter A, Lumley J, Watson L. The 'new' risk factors for SIDS: is there an association with the ethnic and place of birth differences in incidence in Victoria, Australia? Early Human Dev.

18 Budget Items:

COMPONENTS		Details of financial support requested for this project				
(1) PERSONNEL (position, level and % of year.	Salary Rate p.a.	$ Year 1	$ Year 2	$ Year 3	$ Year 4	$ Year 5
Res Fell B.06 X 2	50,111	100,222	100,222	100,222	100,222	100,222
Admin Off HE0 6.05, 0.4	37,774	15,110	15,110	15,110	15,110	15,110
Admin-Ass HEO 3.05, 0.2	26,050	5,210	5,210	5,210	5,210	5,210
MRA HEO 5.01, 1.0 X 2yrs 0.5 X 2yrs	30,083		30,083	30,083	15,041	15,041
	Sub total	**120,542**	**150,625**	**150,625**	**135,583**	**135,583**
(2) EQUIPMENT (specify)						
Filing cabinets		700				
	Sub total	**700**				
(3) MAINTENANCE						
Printing instruments		1,000	3,043	7,085	1,000	
Mothers' information kits			37,500			
Resource kits for professionals		3,920				
Training workshops		58,000		17,800	2,000	
Computing support		4,000				
Production of reports						28,000
Recruitment costs		1,500				
Stationery		1,000	1,000	1,000	1,000	1,000
Envelopes		4,302				
Telephone		2,574	2,200	2,200	2,200	2,200
Postage		400		13,883	14,883	14,884
Other: data monitoring, travel, c/tee support		1,984	5,534	6,934	6,484	7,184
	Sub total	**78,680**	**49,277**	**48,902**	**27,567**	**53,268**
	TOTAL $	**199,922**	**199,902**	**199,527**	**163,150**	**188,851**

Request for salary loading: Y/N N

Full or half loading: F/H

Name :

Qualifications:

Justification for award of a salary loading:

19 Justification of Budget

Justify budget for each year of required funding.

Personnel

Research Fellow Level B06 X 2 - RS and SB are currently funded at this level on VicHealth Project grants. The complex co-ordination of such a large community trial requires appointments at this level over the full course of the project. Although responsibility for many tasks will be shared (eg staff appointments and training; support and liaison with LGAs; analysis and reporting) RS will have primary responsibility for data management and administration tasks and SB for resources development and process and impact evaluation.

Administrative Officer, HEO 6.05 La Trobe Univ General Staff Scales - a .4 appointment is requested at this level to provide administrative support - personnel, finance and general - to the project.

Administrative Assistant HEO 3.05 La Trobe Univ General Staff Scales - this .2 appointment in the trial is necessary to provide basic administrative support for the field staff and trial co-ordinators in production of resources for the project and the newsletter, as well as mailing of process and impact evaluation questionnaires.

Medical Records Administrator, HEO 5.01 La Trobe Univ General Staff Scales - this appointment over four years of the trial is required for the setting up of data systems in each of the 7 intervention communities to enable the rolling mailouts of questionnaires to mothers. The appointee would also be responsible for setting up the data management system at the Centre. As the position requires liaison with local government and an ability to handle a diversity of local government computing systems, some years of experience are required.

Seven Field Staff, HEO 7.01 La Trobe Univ equivalent ($38,507) - THIS COMPONENT OF THE BUDGET IS NOT INCLUDED IN THIS APPLICATION AS FUNDING WILL BE SOUGHT FROM OTHER SOURCES - VicHealth, GPEP, Trusts - sources more appropriate to the health promotion nature of these positions: 7 full-time community development field officers are to be appointed from March 1998 - March 2000, at a level equivalent to that of community development officer appointments in local government (Band 5-7, $36,000 - $40,000). A high level of organisational and personal skills will be required in these positions in assisting steering committees implement the intervention and in liaising with a wide range of individuals and organisations.

Equipment: $700 is the cost of two securable four-draw filing cabinets.

Maintenance

Printing instruments (EPDS and SF-36 and reminder postcards) - a total of 17,000 copies of each instrument is required. Design and printing for the EPDS = $2040 and the SF-36 = $4080. Design and printing of 17,000 reminder postcards = $4500. Printing of 2800 questionnaires for process evaluation = $1508

Mothers' information kits - this item includes the costs of design and printing of 7,500 kits for mothers ($37,500: $5.00 unit cost for 2 leaflets on common difficulties and hints for fathers; 12 page booklet on local services, voucher booklet with 20 vouchers in a card folder).

Resource kits for professionals - background information to accompany the training program in the community intervention trial: article reprints; course guide, etc. $7.00 unit cost for 80 GPs and MCHNs per LGA X 7 LGAs = $3,920.

Computing support - $6,000 for advice on data systems and management involving different local government systems, in the context of a community trial design - $4,000 in Year 1 and $2,000 in Year 4.

Professional workshops - the cost here is for one day workshops for up to 20 participants and calculated on rates charged by psychologists/ psychiatrists skilled in non-directive counselling: $1,000/day (equivalent to $50 per participant) - 1 workshop per LGA for MCHNs (10-20 nurses) and 3 workshops for GPs (50-80 GPs per LGA with 600+ births per year) = 4 workshops X $1,000 X 7 LGAs = $56,000; plus the cost of a half day workshop as part of reviewing practice 18 months after initial training: 4 X $600 X 7 LGAs = $16,800. Room-hire/catering: $3,000.

Stationery - $1,000 per year to cover basic stationery needs for trial coordinators, field and admin staff, newsletter production etc.

Envelopes - a total of 40,000 A5 envelopes are required: 80 boxes X 500 envelopes at a cost of $35.00 per box = $2,800. Half of these will need to be printed with reply paid information at a cost of $1232 for 17,000 envelopes, giving a total envelope cost of $4032. 20,000 sticky address labels = $270.

Postage - 20,000 questionnaires will be mailed out in total at 85c each, plus the same cost for their return by reply paid mail: $1.70 X 20,000 = $34,000. Postage costs for reminder postcards = 17,000x45c = $7650. General postage costs for project administration/communication = $2400 split over the project.

Telephone - the costs requested here, $11,000 over five years, are based on an average of 10 calls per day for each of the trial coordinators, and estimating that approx 1/5 of calls will be STD given that the trial requires considerable telephone communication with the intervention LGAs, two of which are in rural areas. The cost of a new internal phone connection and handset is included in Year 1 = $374.

19 Justification of Budget (cont.)

Justify budget for each year of required funding.

Production of Reports/Presentations - costs include $300 for slides/OHPs and $2000 for the project report (1000 copies). A figure of $2,000 for each of the 14 participating LGAs has also been requested in the final year to enable the trial findings to be launched in each community and for the production of a brochure summary of the findings to be distributed within each community via MCHNs, GPs and community groups.
Recruitment costs - costs for advertising and appointing eight staff to the project = $1500.
Other: Travel/Accommodation/Data monitoring costs/Committee support - total expenditure = $27,120. This comprises accommodation for: visits to rural intervention LGAs by RS/SB (3 times/year X 2 LGAs) and the professional workshop facilitator to run the training program in the two rural LGAs (22 nights X $110 per diem allowance = $2420), and travel of 3,000km (at 50c/km = $1500: $750 in Yr2 and $750 in Yr3) for the two rural community field researchers to attend 4 project meetings in Melbourne and for RS/SB to make 4 visits per year to the rural areas (3000km at 50c/km = $1500 each year for 5 years = $7,500); and for the facilitator (1200km at 50c/km = $600). Included also here are data monitoring costs - $700/year in years 3-5 of the trial to cover travel and expenses for the two members of the data monitoring committee. A total of $14,000 is also requested for providing a small amount of support ($500/year over 4 years) to cover expenses for the 7 steering committees over the life of the project.

20

Salary Requested For Any Named Chief Investigator (A,B,C, or D)

Where salary support for any of the above named investigators is sought within the budget of this application, please provide additional information

(a) Chief Investigator A,B,C or D B

Current Appointment: RESEARCH FELLOW

Current salary: $ 51,111

Source of salary support: Vic Health Project Grant

Salary level sought: $ 50,111

Designation: Level B.06

If present salary is provided within NHMRC Project Grant, specify:

(i) Chief Investigator — Surname / Title / Initials

(ii) Project Title

(iii) Commencing year of Grant

(b) Chief Investigator A,B,C or D C

Current Appointment: RESEARCH FELLOW

Current salary: $ 51,111

Source of salary support: Vic Health Project Grant

Salary level sought: $ 50,111

Designation: Level B.06

If present salary is provided within NHMRC Project Grant, specify:

(i) Chief Investigator — Surname / Title / Initials

(ii) Project Title

(iii) Commencing year of Grant

(c) Chief Investigator A,B,C or D

Current Appointment:

Current salary: $

Source of salary support:

Salary level sought: $

Designation:

If present salary is provided within NHMRC Project Grant, specify:

(i) Chief Investigator — Surname / Title / Initials

(ii) Project Title

(iii) Commencing year of Grant

(d) Chief Investigator A,B,C or D

Current Appointment:

Current salary: $

Source of salary support:

Salary level sought: $

Designation:

If present salary is provided within NHMRC Project Grant, specify:

(i) Chief Investigator — Surname / Title / Initials

(ii) Project Title

(iii) Commencing year of Grant

21 Research Personnel

For known personnel for whom salaries at Research Assistant level (using NHMRC salary scales) and above are sought within the budget (Q. 20), other than those listed as Investigators (Q. 9), please complete the following:

(a) Surname Title Initials

Current Appointment Current salary $

Source of salary support

Salary level sought $ Designation

If present salary is provided within NHMRC Project Grant, specify

(i) Chief Investigator Surname Title Initials

(ii) Project Title (iii) Commencing year of Grant

Qualifications:

Year	Conferring Institution	Degree

(b) Surname Title Initials

Current Appointment Current salary $

Source of salary support

Salary level sought $ Designation

If present salary is provided within NHMRC Project Grant, specify

(i) Chief Investigator Surname Title Initials

(ii) Project Title (iii) Commencing year of Grant

Qualifications:

Year	Conferring Institution	Degree

22 NHMRC RESEARCH SUPPORT

Please list in the following order:
(i) PAST SUPPORT (for previous six years, excluding any projects currently supported
(ii) CURRENTLY HELD (at time of submission of this application
(iii) REQUESTED (for next year excluding this application)
TIME COMMITMENT TO ALL GRANTS (for next year, including this and all other applications, as well as any grants already held for next year)

Any Chief Investigator on this application who holds another NHMRC grant for next year (or is named as a Chief Investigator on another NHMRC application for next year must provide details of their proposed level of commitment (ie listing the average number of hours per project) on ALL NHMRC grants that could be held next year.

TITLE (Do not vary from NHMRC recorded title)	INVESTIGATORS	HRS/WK Each Investigator	YEARS From 19__ to 19__	FUNDS for each year	PUBLICATIONS See Instruction Booklet
(i) The Congress Alukura Research and Development Project	Bell S<br>Lumley J	4<br>*	1991	31,393	n/a
*two visits to Alice Springs					
Evaluation of pre-pregnancy counselling and information in inner urban Melbourne	Lumley J		1991-94	18,263	
Short-term predictionof HIV infection and AIDS in Australia (CARG)	Becker, NG<br>Carlin, JB<br>Watson, LF		1990<br>1991	74,978<br>64,191	LW 12, 18, 20
(ii) Postnatal and infancy study of Vietnamese Turkish and Filipino women (b)	Lumley J<br>Small R<br>Yelland J<br>Rice P	2.5<br>20.0<br>8.0<br>4.0	1994-96	48,312<br>48,310<br>15,324	see report
PHRDC Public Health Travelling Fellowship: Design of randomised trials for evaluating perinatal and early postnatal public health interventions	Brown S	12 wks F/T	Sept - Dec 1996	16,330	
Childbearing and cultural beliefs and practices amongst Southeast Asian women (PHRDC)	Rice PL<br>Watson, L	15<br>4	1996<br>1997<br>1998	56,928<br>53,271<br>41,033	

If the space provided is insufficient, please insert a photocopy of this sheet, to record further NHMRC research support

22 NHMRC RESEARCH SUPPORT

Please list in the following order:
(i) PAST SUPPORT (for previous six years, excluding any projects currently supported
(ii) CURRENTLY HELD (at time of submission of this application
(iii) REQUESTED (for next year excluding this application)
TIME COMMITMENT TO ALL GRANTS (for next year, including this and all other applications, as well as any grants already held for next year)

Any Chief Investigator on this application who holds another NHMRC grant for next year (or is named as a Chief Investigator on another NHMRC application for next year must provide details of their proposed level of commitment (ie listing the average number of hours per project) on ALL NHMRC grants that could be held next year.

TITLE (Do not vary from NHMRC recorded title)	INVESTIGATORS	HRS/WK Each Investigator	YEARS From 19__ to 19__	FUNDS for each year	PUBLICATIONS See Instruction Booklet
(iii) Home early trial: a randomised trial of very early obstetric discharge					
	Lumley J	4	1997 -	191,766	
	Brown S	30	2000	197,580	
	Small R	4		196,480	
	Crowther C	3		151,619	
	Jackson T	1			
	Pannifex J	1			
	Turnbull D	4			
	Waldenstrom U	2			

If the space provided is insufficient, please insert a photocopy of this sheet, to record further NHMRC research support

(20) a

23 RESEARCH SUPPORT FROM OTHER SOURCES

Please list in the following order:
(i) PAST SUPPORT (for previous six years, excluding any projects currently supported)
(ii) CURRENTLY HELD (at time of submission of this application)
(iii) REQUESTED (for next year)

TITLE	INVESTIGATORS	YEARS From 19__ to 19__	FUNDS for each year	Source of Support
(i) Satisfaction with maternity care	Lumley J	1989-90<br>1yr	26,995	VHPF
The effect of exercise in pregnancy on fetal outcome	Bell R<br>Lumley J	1989-92<br>(3 yrs)	24,733<br>24,733<br>23,317	VHPF
Ultrasound as a screening device for fetal malformations	Lumley J<br>Bell R<br>Yates J	1990-92	21,243<br>22,538<br>5,293	VHPF
A randomised trial of therapeutic ultrasound in the treatment of postpartum breast engorgement	McLachlan Z<br>Milne J<br>Lumley J<br>Walker B	1987-89	5,000	Queen Victoria Hospital Research Fund
Mothers' and Children's Health	Lumley J	1991-94<br>Feb	281,110<br>305,278<br>331,556	Program grant VHPF
Postnatal depression: a follow-up study	Lumley J<br>Brown S<br>Small R<br>Astbury J	1990-93<br>Aug	61,001<br>65,137<br>19,232	VHPF
Pregnancy outcomes in IVF and GIFT	Lumley J<br>Venn A	1990-93<br>Aug	39,893<br>40,842<br>41,321	VHPF

If the space provided is insufficient, please insert a photocopy of this sheet, to record further NHMRC research support

23 RESEARCH SUPPORT FROM OTHER SOURCES

Please list in the following order:
(i) PAST SUPPORT (for previous six years, excluding any projects currently supported)
(ii) CURRENTLY HELD (at time of submission of this application)
(iii) REQUESTED (for next year)

TITLE	INVESTIGATORS	YEARS From 19__ to 19__	FUNDS for each year	Source of Support
(i) Does prolonged breastfeeding protect against premenopausal breast cancer? [extended to July 1994; * return to US]	Lewis P*<br>Giles G<br>Lumley J	1991-92<br>Mar Mar	51,749	VHPF
Breast cancer and IVF	Venn A<br>Lumley J<br>Healy D<br>King C	1993	5,000<br>2,000	Monash IVF<br>IVF Friends
Trends in risk factors for SIDS in Victoria	Lumley J	1992-93	10,987	SIDRF
Family environments at very low and very high risk of SIDS	Lumley J<br>Gifford S<br>Potter A<br>Yelland J	1990-93	46,377<br>43,275<br>43,275	National SIDS Council
Intra-uterine hypoxia and SIDS	Cooper R<br>Lumley J	1992-93	18,000	SIDA, ACT
Breast cancer and IVF	Venn A<br>Lumley J<br>Healy D<br>King C	1993-94<br>1yr	30,068	RADGAC
Intra-uterine hypoxia and SIDS	Cooper R<br>Lumley J	1993	52,400	South Aust SIDS Research Foundation

If the space provided is insufficient, please insert a photocopy of this sheet, to record further NHMRC research support

23 RESEARCH SUPPORT FROM OTHER SOURCES

Please list in the following order:
(i) PAST SUPPORT (for previous six years, excluding any projects currently supported)
(ii) CURRENTLY HELD (at time of submission of this application)
(iii) REQUESTED (for next year)

	TITLE	INVESTIGATORS	YEARS From 19__ to 19__	FUNDS for each year	Source of Support
(i)	A survey of nurses employed in General Practitioners' rooms	Bonawit, V<br>Watson, L	1993<br>1994	1,914	School of Nursing, Faculty of Health Sciences, La Trobe University
	A survey of registered midwives in Victoria	Donohue, L<br>Watson. L<br>Potter, A	1995<br>1996	7,042	School of Nursing, Faculty of Health Sciences, La Trobe University
(ii)	Postnatal and infancy study of Vietnamese, Turkish and Filipino women (a)	Lumley J<br>Small R<br>Yelland J<br>Rice P	1994-96	84,330<br>84,849<br>29,071	VHPF
	Experience of childbirth, satisfaction with care and emotional well-being of recent mothers	Lumley J<br>Brown S	1994-96	77,595<br>57,103<br>58,245	VHPF
	Depression after birth:Developing methods for transcultural assessment.	Small R<br>Lumley J	1996	28,000	Australian Rotary Health Research Fund
	Study of breast and ovarian cancer after infertility and IVF	Venn, AJ<br>Watson, LF<br>Healy, DL<br>Giles, GG	1996<br>1997<br>1998	50,000<br>50,000<br>50,000	Kathleen Cunningham Breast Cancer Research Foundation
			1996	12,299	Anti-Cancer Council of Victoria
	Childbirth and health of women from South East Asia	Rice, PL<br>Watson, LF	1996<br>1997<br>1998	30,523<br>36,078<br>37,163	Victorian Health Promotion Foundation

If the space provided is insufficient, please insert a photocopy of this sheet, to record further NHMRC research support

(21) 6

24 Progress Report on Current Project Grant(s):

Project Number and Scientific Project Title

944128 Postnatal and infancy study of Vietnamese, Turkish and Filipino women

Chief Investigator(s):

	Surname	Title	Initials
a)	Lumley	Dr	JM
b)	Small	Ms	R
c)	Yelland	Ms	J
d)	Rice	Dr	PL

Administering Institution: La Trobe University

Period of Grant Support: 1994-96

Progress Report:

The project aims to compare experiences of the maternity care system, satisfaction with care, events and procedures during birth, and to make a standardised assessment of emotional well-being (Edinburgh Postnatal Depression Scale and SF-36) in Turkish, Vietnamese and Filipino women 6-7 months after birth. A major feature of the project has been the employment and training of bilingual research workers to conduct the interviews with women and the careful attention paid to the development of culturally appropriate translations via standard professional translation procedures used in conjunction with focus group assessment of translations and extensive piloting of instruments.

Recruitment for the project was completed in November 1995 with 151 Vietnamese, 147 Filipino and 140 Turkish women agreeing to take part in the study. Follow-up personal interviews at home, conducted by the bicultural interviewers in women's own languages 6-7 months after the birth of their babies, have been progressing well: 256 are now complete (92 Filipino, 82 Vietnamese and 82 Turkish). The target of at least 100 interviews in each group will be reached by April 1996.

Coding and data entry of hospital recruitment questionnaires is approaching completion and the coding of the home interviews has been undertaken concurrently with their completion and as much as possible in order to provided feedback to the interviewers. Translation and transcription of a small selection of the interviews for quality control purposes has been undertaken and further transcriptions of a random 20% of the interviews is in progress for purposes of qualitative analysis.

Two conference presentations were given during 1995, dealing with the transcultural methodological issues tackled in the project. Data analysis will commence as soon as the final interviews are completed.

Publications arising from this Grant:

Prepared By: Rhonda Small | Signature: Rhonda Small
