# Supplementary material for: Improving Population-Level Maternal Health: A Hard Nut to Crack? Long Term Findings and Reflections on a 16-Community Randomised Trial in Australia to Improve Maternal Emotional and Physical Health after Birth [ISRCTN03464021]
Source: PLoS One. 2014 Feb 28;9(2):e88457. doi: 10.1371/journal.pone.0088457 (PMC3938427; doi:10.1371/journal.pone.0088457)
Supplement: Protocol S1 — PRISM protocol, BMC Public Health 2003. (PDF) [file pone.0088457.s004.pdf]

BMC Public Health

BioMed Central

Study protocol

Open Access

PRISM (Program of Resources, Information and Support for Mothers) Protocol for a community-randomised trial [ISRCTN03464021]

Judith Lumley*[1], Rhonda Small[1], Stephanie Brown[1], Lyndsey Watson[1], Jane Gunn[2], Creina Mitchell[3] and Wendy Dawson[4]

Address: [1]Centre for the Study of Mothers' and Children's Health, La Trobe University, 251 Faraday St, Carlton, Victoria 3053, Australia, [2]Department of General Practice, University of Melbourne, 200 Berkeley St, Carlton, Victoria 3053, Australia, [3]School of Nursing, La Trobe University, Bundoora, Victoria 3086, Australia and [4]Casey Hospital, Southern Health, Locked Bag 29, Clayton South Victoria 3169, Australia

Email: Judith Lumley* - J.Lumley@latrobe.edu.au; Rhonda Small - R.Small@latrobe.edu.au; Stephanie Brown - Stephanie@latrobe.edu.au; Lyndsey Watson - L.Watson@latrobe.edu.au; Jane Gunn - J.Gunn@unimelb.edu.au; Creina Mitchell - C.Mitchell@latrobe.edu.au; Wendy Dawson - Wendy.Dawson@southernhealth.org.au

* Corresponding author

Published: 20 November 2003

BMC Public Health 2003, **3**:36

Received: 28 July 2003

Accepted: 20 November 2003

This article is available from: http://www.biomedcentral.com/1471-2458/3/36

© 2003 Lumley et al; licensee BioMed Central Ltd. This is an Open Access article: verbatim copying and redistribution of this article are permitted in all media for any purpose, provided this notice is preserved along with the article's original URL.

Abstract

Background: In the year after birth one in six women has a depressive illness, and 30% are still depressed, or depressed again, when their child is 2 years old, 94% experience at least one major health problem (e.g. back pain, perineal pain, mastitis, urinary or faecal incontinence), 26% experience sexual problems and almost 20% have relationship problems with partners. Women with depression report less practical and emotional support from partners, less social support overall, more negative life events, and poorer physical health. Their perceptions of factors contributing to depression are lack of support, isolation, exhaustion and physical health problems. Fewer than one in three affected women seek help in primary care despite frequent contacts.

Methods/Design: PRISM aims to reduce depression and physical health problems of recent mothers through primary care strategies to increase practitioners' response to these issues, and through community-based strategies to develop broader family and community supports for recent mothers.

Eligible local governments will be recruited and randomised to intervention or comparison arms, after stratification (urban/rural, size, birth numbers, extent of community activity), avoiding contiguous boundaries. Maternal depression and physical health will be measured six months after birth, in a one year cohort of mothers, in intervention and comparison communities. The sample size to detect a 20% relative reduction in depression, adjusting for cluster sampling, and estimating a population response fraction of 67% is 5740 × 2. Analysis of the physical and mental health outcomes, by intention to treat, will adjust for the correlated structure of the data.

Background

Maternal health after birth

Depression

Depression is relatively common in the months after birth. The point prevalence of probable depression, assessed with the Edinburgh Postnatal Depression Scale

(EPDS score ≥ 13) was 16.9% (95%CI 14.9–18.9) six months after birth [1], and 15.4% (95%CI 12.8–18.0) eight to nine months after birth [2] in two Australian population-based studies. The EPDS was developed to avoid problems with somatic and social items (e.g. change in appetite, sleep disturbance, 'getting out') in other standard measures which cannot be interpreted as morbidity in women with a small baby [3]. It has been found to have a sensitivity of 86%, 95%, 68% and a specificity of 78%, 93%, 96% when assessed against a psychiatric diagnosis of depression in three studies in the UK [3-5]. The EPDS has also been validated against clinical psychiatric diagnosis in an Australian population [6]. In a subsequent Australian study all women with two EPDS scores >12 in the 3 months after birth had a diagnosis of depression at subsequent psychiatric interview [7]. Maternal depression has an impact beyond the affected women themselves to other family members, with substantial evidence of negative effects of maternal depression on child development [8-10].

A follow-up study of women who had – and had not – scored as depressed in the first population-based survey found that 30% of women who had been depressed at eight to nine months were depressed 21 to 30 months after the index birth [11]. Their depression was not necessarily brief, nor self-limiting. Only a third of women who had been depressed had sought help from any health professional. When they had sought care it was usually in primary care, from a general practitioner (GP) or maternal and child health nurse (MCHN). Only 15% of women with depression had sought help from, or been referred to, a mental health professional [11]. These findings demonstrate the necessity for facilitating disclosure and improving the recognition of maternal depression in primary care settings.

Women in the follow-up study who had been depressed after birth, reported less practical and emotional support from their partners and saw themselves as having less social support overall than women who had not been depressed [12]. Women in the case group had also experienced more negative life events, were somewhat more likely to have a child with a 'difficult' temperament, and to be in poorer physical health as assessed by standardised questionnaires [12]. Their own unprompted responses to a question asking about factors contributing to depression gave a picture very congruent with the questionnaires: feeling unsupported, isolated, exhausted and having physical health problems. Half had turned to family and friends but half had talked to no-one about how they felt: marital disharmony and lack of support have been described as among the most consistent social associations of depression after birth [13].

When asked two years after birth what advice they would give to other women in the same circumstances the major response was 'find someone to talk to'. This someone did not have to be a health professional or a counsellor. Women who did find someone who listened with empathy described this as very helpful in dealing with depression and with physical health problems. The second piece of advice was to get some 'time-out' from looking after the baby [11].

Physical health problems after birth

A population-based study in Victoria, Australia [1] and large hospital cohorts in the UK [14,15] and USA [16], using different study designs, have consistently identified specific health problems as very common in the year after birth (e.g. back pain, perineal pain, mastitis, haemorrhoids, urinary incontinence). Sexual problems and relationship difficulties are also common, and severe fatigue affects two in three women: 94% of Victorian women experienced at least one of these problems in the first six months after birth [1]. Fewer than a quarter sought help from their GP [1]. Complex associations between physical health problems and depression were also identified [17]. Despite the persistence of symptoms for one year or more in two of the UK studies, only half of affected women sought treatment, with even lower consultation rates for perineal pain (21%), urinary incontinence (27%) [18] or faecal incontinence (14%)[19].

As large numbers of women giving birth in Australia were born overseas in countries where English is not the first language, a home-based interview project using the same questions as the second state-wide survey was carried out with Vietnamese, Turkish and Filipino women six months after birth (*Mothers in a New Country, MINC)*. Findings with respect to physical and emotional health problems, and unwillingness to discuss these with GPs were very similar. The factors most commonly identified by women in that study as contributing to their depression were similar to those described by Australian women, with the addition of some associated with migration [20,21].

Utilisation of primary care after birth

Contacts with MCHNs in Victoria, where the population-based surveys were carried out, according to the *Maternal and Child Health Programme Report 1995–6*, included a home visit soon after hospital discharge to 94% of mothers and participation rates in the key infant 'ages and stages' visits at two, four and eight weeks, and four to eight months of 87–96%. New mothers' groups run by the MCHN were attended by 60% of first-time mothers [22]. Thus the lack of identification of maternal health problems in the state-wide Maternal and Child Health Program is not a matter of lack of contacts. However, the *Maternal and Child Health Consumer Survey Project Report*

[23] indicated much lower levels of satisfaction with the service in relation to maternal issues (support and reassurance on maternal health, counselling, referral and information), than with child issues (monitoring child development support and reassurance on child health, feedback on the child's progress).

Analysis of the Health Insurance Commission data for a random sample of recent mothers in Victoria showed that the mean number of visits to GPs by a mother/baby dyad in the six months following birth was 7.7 [24]. Similarly, in a large Victorian survey over 92% of GPs described themselves as involved in postnatal care, but neither the common physical health problems described above, nor depression, were issues which GPs considered part of routine postnatal care, and more tellingly, both were areas where the GPs rated themselves as not very confident [25].

Our response to this body of evidence was that what was needed were universal, community-based, primary care strategies which would re-focus the existing health care contacts on maternal health issues, both emotional and physical; local strategies for support and increasing the availability and accessibility of 'time-out' for mothers; better information about local services and encouragement and incentives to use them.

Evidence available from randomised trials, by 1996, on reducing depression after birth

One randomised trial of an antenatal intervention to reduce postnatal depression, in women at increased risk of depression, had been published [26] as had one antenatal controlled but non-randomised trial, also recruiting women at increased risk [27], and one innovative antenatal cluster-randomised trial recruiting unselected pregnant women, not adjusted for clustering [28]. The randomised trial was not able to detect a significant reduction in depression.

One postnatal trial, a group intervention led by psychologists, offered to women assessed as being probably depressed, with a focus on sharing experiences and problem-solving, did not reduce depression [29]. Two other postnatal trials which recruited women with clinically confirmed depression reported a marked reduction in depression with individual counselling at home over six [30] or eight weeks [31], carried out by child health nurses [30] and health visitors [31]. A subsequent dissemination project in the UK identified greater confidence in dealing with maternal depression, and a decrease over time in health visitors' need to refer to other agencies, as well as reduced levels of depression [32]. Thus, although there were no postnatal trials of interventions applicable to all recent mothers and no trials of community-implemented interventions, there was evidence that with additional training the existing primary care providers – MCHNs have a similar role to health visitors and child health nurses – could provide an effective therapeutic response to women who are depressed.

Other evidence relevant to the proposed intervention

The importance of community-based interventions in mental health had been argued by Regier and colleagues [33] because only a minority seek professional help for mental health problems, when they do they turn to the primary health sector; and even when help is sought mental health problems are under-recognised in primary care. These arguments were reinforced by the data presented earlier about women's non-disclosure of depression after birth. The effectiveness of teaching 'empathic' listening skills to GPs for improved care of patients with depression and anxiety had been similarly demonstrated [34]. Systematic reviews, summarised by Davis and colleagues [35], showed that changing GP knowledge, skills, and confidence requires multifaceted interventions. These findings informed the interventions developed for working with GPs and MCHNs in *PRISM*.

In 1996, social support interventions for new mothers were supported by very limited trial evidence [36,37] and whole community natural helping interventions were largely supported by case study evidence only [38,39].

In 1996, there were three planned or on-going randomised trials in the UK designed to reduce depression and improve physical health: the provision of mothers' information kits and the establishment of mothers' groups within a factorial design in Scotland; additional practical home support in Sheffield, England; and extended and re-focussed, evidence-based community midwifery care in the West Midlands Region, England [40]. All three planned to use the same health outcome measures (the Edinburgh Postnatal Depression Scale (EPDS) and the health status measure Short Form 36 (SF-36), and all planned an economic evaluation. The first two of these trials developed interventions to be delivered alongside existing services and women had the option of taking up the innovative programs. The third trial in the West Midlands involved 'enhanced' care within the clusters allocated to the intervention. *PRISM* will include both 'enhanced' primary care and additional community components. These trials offer complementary strategies alongside or within existing services.

Rationale for the choice of cluster (community) randomisation

Evaluation of any intervention, including measurement of health outcomes is essential. There were three reasons for randomising communities rather than individuals. The first was that the components of the overall intervention needed to occur at a community level (changing

environments, 'befriending' activities, MCHN training and development). The second was that social ecological theory proposes that the person-centred (information, vouchers, encouraging help-seeking) and environmental change aspects are synergistic and mutually supportive [41]. The third was that if the intervention were to be effective it would need to be implemented at a community level because of the key role of local government, the organisation of the MCHN service and Divisions of General Practice.

Design of the intervention

Aims

The Program of Resources, Information and Support for Mothers (*PRISM*) has two principal aims:

1. to decrease the prevalence of depression six to nine months after birth from an expected rate of 16.9% in the communities without a specific intervention program to 13.9% (or less) in the intervention communities, and to improve the health status of women on all domains of the SF-36 in intervention communities compared with comparison communities;

2. to reduce the proportion of women still depressed two years after the birth from 30% of those who were depressed at six to nine months to 20%.

The secondary aims are:

3. to develop and test a model for inter-sectoral collaboration on a public health issue at the local level involving primary care (general practitioners and maternal and child health nurses), mental health services, local government and community organisations, alongside locally facilitated social network interventions;

4. to measure the effectiveness of the proposed intervention by locating the program within a randomised trial design and including process, impact and health outcome evaluation, as well as a comprehensive economic evaluation.

The intervention

The intervention is based in social ecological theory [41]. This hypothesises that for both success and sustainability, programs should be developed around existing high impact 'leverage points' in communities (such as GPs and community organisations) and interventions should be both person-centred (e.g. aimed at encouraging help-seeking and information-seeking by mothers) and environment-centred (e.g. creating mother-and-baby-friendly environments).

The intervention will have two components, one directed to primary care, the other to community services (local government and community agencies), with a small steering committee of key stakeholders (local government, GPs, MCHNs, community and consumer organisations) locally appointed to coordinate the implementation of the intervention, supported by a community development officer (CDO) in each intervention community.

In primary care the objectives are:

- increased recognition of depression in mothers of young children at all primary care contacts;

- an active response to the recognition of depression by primary care providers; explicit offer of time to talk by both MCHNs and GPs;

- increased recognition and treatment of physical problems which are common in the year after birth.

The strategies will include:

- provision of a training program for MCHNs comprising information about the prevalence, associated factors and implications of depression after birth and health after childbirth generally, as well as skills training in non-directive counselling/active listening;

- provision of a multifaceted GP education program (*Guidelines for Assessing Postnatal Problems: GAPP*)[i] involving written guidelines for care based on thorough reviews of research, an information session on common problems, skills training in active listening, practice audit, opportunity to apply the guidelines using a simulated patient followed by feedback, peer discussion, professional support and a practice-reinforcing strategy;

- the establishment of professional peer support programs for both MCHNs and GPs;

- with the assistance of the steering committees, local government and Divisions of General Practice, the development of networks between those in different primary care roles (GPs and MCHNs) and with existing self-help groups; the development of links between the primary care network and the local community psychiatric services so that support can be offered in primary care in a context of accessible consultation, liaison and referral.

The professional training programs will be implemented as the first stage of the intervention in each community, ensuring that once other elements of the program are put into place, women receive an appropriate response from primary caregivers. We intend to identify, through the

initial training programs, primary care professionals who are enthusiastic about the intervention and willing to encourage their peers, and to seek their involvement in local steering committees, along with other key stakeholders (local government, community and consumer organisations).

In local government and community agencies the objectives are:

• to increase the availability and accessibility of support and 'time-out' for recent mothers;

• to provide better information about local services to mothers and families, with encouragement and incentives to use them;

• to increase the 'mother-and baby-friendliness' of local environments (e.g. shopping centre car spaces for parents with prams, improved baby-change facilities);

• to increase inter-organisational collaboration and advocacy for parents and young children.

The strategies will include:

• an assessment of the availability and accessibility of relevant services (occasional child care, recreational services, library, information and counselling services, neighbourhood houses, community centres and community health centres, as well as shopping centre facilities) with a focus on the extent to which they are 'mother-and baby-friendly';

• the development of an information kit for mothers comprising a listing of local services for mothers and babies, a brochure outlining some of the common difficulties of being a mother and some strategies for dealing with these which other women have found helpful, an information ('useful tips') sheet for fathers and a booklet of free service vouchers for recent mothers. The latter might for example include session(s) at the local occasional child-care service, free entry at the local swimming pool, a series of relaxation classes at the maternal and child health centre, etc. The specific nature of such vouchers will depend on what is feasible and appropriate in each local community. The kit will be given to all recent mothers by the MCHN during the statutory home visit made soon after hospital discharge;

• the establishment of a mother-to-mother support network based on the principle of non-professional befriending. The format of the network will be selected after focus group work and individual interviews in each intervention community. Examples of some models are: calling for volunteer older women to provide support to recent mothers via a monthly visiting program ('grandmother' scheme); putting two women who have babies the same age in touch with each other for mutual support such as occasional babysitting, getting out together etc. ('peer' support model); developing a pool of mothers with older children who could 'adopt' a mother with a new baby for advice and support (experienced' mothers model). Both self-referral and referral from primary care will be considered.

A community development officer will be appointed in each intervention community to:

• liaise with local government and non-government agencies, primary caregivers and local psychiatric services;

• assess levels of community service provision, compile information on services for mothers, solicit voucher contributions from relevant bodies, and assist in the production of the mothers' information kits;

• assist in the establishment of the supportive social network;

• provide support to the steering committee in overseeing the intervention (in the initial 12–18 month establishment phase) and the integration of the community service program once established, within ongoing service provision by local government or community agencies.

Although the major components of the community intervention are defined, flexibility is essential to allow each local steering committee to make decisions about other supportive interventions as well as the appropriate implementation of the different elements of the intervention. For example some steering committees may feel that organising a community forum to raise awareness about the issue is appropriate, others may want to publicise the program in the local papers. It is proposed that a regular newsletter be produced by the research team keeping the steering committees in touch with the development of the programs in each LGA, thus enabling the sharing of ideas, common difficulties and creative solutions to practical problems in establishing the programs.

Methods/ Design

The first stage will be mapping and documentation with respect to all Victorian local government authorities (LGAs), Department of Human Services regions, and General Practice Divisions. This process includes an assessment of the degree of overlap between these areas, compilation of the most recent census data for LGAs; the identification of key personnel in each LGA; a review of each of the Municipal Public Health Plans; the

identification of research and intervention projects in each area to assess recent community activity and to identify projects relevant to *PRISM* objectives and strategies. The next step is seeking support from the peak local government body, the Municipal Association of Victoria, and when that is achieved preparing and distributing an information package about the intervention and evaluation to all eligible LGAs.

Cluster participants

Eligibility for community participation is defined in terms of the number of births/year (300 to 1,500). That range was chosen to facilitate both rural and metropolitan participation, to provide diversity of participating LGAs, to take into account the probable workload for a Community Development Officer, and to ensure an adequate sample size.

Thirty-five local government authorities (LGAs) in Victoria had 300 to 1,500 births in 1996. Two will be excluded from the invitation to participate because of difficulties which they pose for matching[ii]. The remaining 33 LGAs will be approached with information about *PRISM* and offered a detailed briefing on site. The presentation will include the background to the study, the rationale for the intervention, the rationale for carrying out the intervention as a community-randomised trial, the important role of comparison communities in trials, a specification of the roles and responsibilities of the research team and the LGA in *PRISM*, and a specification of what will be provided by each of the parties in terms of direct and indirect costs and in-kind support. A draft Memorandum of Understanding will be provided for discussion.

The aim is to recruit LGAs to be matched in pairs, taking into account the number of births/year, socio-demographic characteristics, area characteristics such as geographic size, rural/metropolitan location, and the extent of recent and current community activity. Randomisation of the LGAs will occur within pairs assigning one LGA to the intervention program, the other to be a comparison community. The LGAs will be non-contiguous to reduce the likelihood of 'contamination' of the comparison communities. Randomisation will take place at a public event to reassure communities about lack of bias in the process and its freedom from external political and other pressures.

Individual participants

In each participating community the individuals whose health outcomes will be measured are women giving birth over a 12 month period who have a live-born child surviving the neonatal period. Women having multiple births will be included as will women having a second or later child.

Sample size

The sample size to detect the first stated aim, namely an absolute reduction in the community prevalence of depression of 3% (16.9 to 13.9%), that is a relative reduction of just under 20%, with an $\alpha = 0.05$ (2-sided), $\beta = 0.20$, would be 2337 in each group if individuals were randomised. This sample size would be more than able to detect differences of clinical importance (e.g. it would be possible to detect half the mean difference between population scores and people of the same age/sex consulting a GP for a minor medical condition) [42] in the SF-36 domains, or differences of clinical importance in the summary mental and physical scores of the SF-36.

The sample size given community (cluster) randomisation is increased by the need to take into account the clustered binary response data, to an estimated 3,800 in both intervention and comparison communities. This estimate was obtained using power calculations [43], the number of births in each LGA in 1996, the depression prevalence data described above and a cost analysis using the budget estimates of community and individual costs [44]. This shows that seven matched pairs of clusters, including two rural pairs, gives the maximum power most economically. The expected number responding from each cluster (about 67% of those surveyed) ranges from 200 to 1000, with a geometric mean of approximately 820, giving 5,740 births in each arm. A 12 month data collection period is likely to be sufficient to achieve the sample size required, based on the proportion of recent mothers who have responded to a mailed questionnaire in two statewide surveys (72% and 63%) [45,46].

Given the depression prevalence estimates of 16.9% to 13.9% and an adjusted response fraction to the postal survey of 67% there would be 535 to 650 women with depression responding at six months from intervention communities and 650 from comparison communities. If we assume a response fraction of 80% by these women to the second survey at 24 months, the sample size is large enough to identify a one-third difference in the prevalence of depression (20% vs. 30%), $\alpha = 0.05$ (2-sided), $\beta = 0.20$, using the same design effect for clustering as at six months. The power of the follow-up study is likely to be substantially increased beyond that estimation by the availability of women's earlier EPDS and SF-36 scores and the inclusion of these earlier scores as covariates in the analysis of health outcomes. Thus the follow-up study is robust to a lower response fraction than predicted. Alternatively, it could detect a smaller difference than a one-third reduction in the prevalence of depression two years after birth.

Although it would be desirable to have baseline data on the EPDS and the SF-36 in each of the participating

communities at the start of *PRISM* that will not be possible, primarily because of the costs of measurement. The point prevalence of maternal depression six to nine months after birth which has been measured across Victoria in two population-based surveys [1,2] will be measured in a third state-wide survey immediately before the start of health outcome assessment in *PRISM*. Data on the SF-36 three and six months after birth will be available from the standard care arms of two postnatal randomised trials being carried out in Victoria (Jane Gunn *unpublished data*, Rhonda Small *unpublished data*).

An interim analysis will be carried out after the first three months of outcome data collection so that the Data Monitoring Committee (Professor Janet Hiller, University of Adelaide and Professor John Carlin, University of Melbourne) can assess the extent of intra-cluster correlation, and review the study plan, providing advice on whether the sample size, and thus the period of outcome data collection, needs to be increased.

Data collection

Outcome evaluation

Outcome evaluation will not begin until there is evidence that implementation is established in all intervention communities.

In Victoria, births to mothers resident in each LGA are notified by the hospital of birth and home birth practitioners to the LGA. This process facilitates the statutory home visit by the Maternal and Child Health Program soon after postnatal hospital discharge. These birth data will be the basis for the measurement of maternal health outcomes by postal questionnaire. The questionnaire format will be based on the earlier state-wide surveys of recent mothers [1,2]. The content will be piloted extensively, and there will also be tests of the mail-out processes in all participating LGAs.

Assessment of the major health outcomes (depression and general health) will be made using the EPDS and the SF-36 health status measure mailed out to mothers six months after birth. Specific health problems will also be identified in the questionnaire [1,14,15].

The questionnaires, covering letters and prepaid reply envelopes will be prepared at the research site and sent in batches to the LGAs, where an LGA identifier will be added to the questionnaire. The research team will not have access to the LGA data systems. In each LGA a defined liaison person will send out questionnaires each week to women whose baby's date of birth was in the corresponding week six months earlier. A reminder/thank you card will be sent two weeks later. Women whose babies have died will be excluded. The covering letter will give a brief explanation of the study, provide the names and telephone numbers of research team members for queries, and give the name and contact number for the La Trobe University Human Research Ethics Committee, in case any recipient wishes to seek more information or make a complaint.

All women scoring as 'probably depressed' on the six month EPDS, and an equivalent random sample of those scoring as not depressed, will be sent an EPDS, an SF-36 and a specific health problems questionnaire two years after the birth, again on a rolling basis. The inclusion of a random sample of mothers not depressed will ensure confidentiality regarding women's depression status as the mail-out will use the same local government birth registration records at two years as at six months.

Process and impact evaluation

There is clearly a need for detailed process and impact evaluation to assess each intervention community's exposure to the intervention program and its immediate impact before outcome evaluation occurs. The central questions which need to be addressed in process and impact evaluation will be the focus of developmental work in the first twelve months of the trial. A preliminary overview of the sorts of monitoring which will be required is given in Table 1 [see Additional file 1].

Professional education

Have the workshops consistently covered the content in the way intended? Is this true for all LGAs? (independent participant assessment)

Have the workshop personnel completed their tasks satisfactorily? (style, delivery, facilitating discussion – independent participant assessment)

Have the workshops been attended by the majority of primary health care professionals in the area? (attendance records compared to total numbers of GPs and MCHNs)

How was this program received/evaluated by them? (evaluation sheets completed at the end of all sessions)

Information kit for mothers

What responses do recent mothers have to the information kit? (focus group discussions with recent mothers in pilot/development phase; questions included in a survey of a random sample of 100 mothers in each intervention LGA during process evaluation phase)

Are MCHNs happy to hand it out? (telephone survey of MCHNs)

Are all recent mothers receiving the information kit? (questions asked in mothers' survey)

Has there been sufficient local interest to generate at least 20 service vouchers for mothers? (documentation of contributors to voucher booklet in each LGA)

Is there evidence that women are using these 'time-out' vouchers? (service providers to be asked to keep presented vouchers, telephone check of a sample of service providers in each LGA)

Primary care offers of 'time to talk'

Are mothers being encouraged to talk about emotional health issues by MCHNs and GPs? (questions asked in mothers' survey)

Do mothers find this helpful? (questions asked in mothers' survey)

Do primary care professionals feel comfortable with this role? (telephone interviews with MCHNs and GPs)

Is there any evidence of networking between MCHNs and GPs and with specialist mental health services, concerning recent mothers? (telephone interviews with MCHNs, GPs and mental health services)

Local community support for the project

Has there been a satisfactory level of local commitment to the project? (Are there regular steering committee meetings? good attendances? level of participation of members? Is there council community services officer support for trial? Is there local GP Division: support?) Is there documentation of the role and activities of all of these in establishing the intervention in each LGA and of voluntary agencies involvement) Has the project received appropriate local publicity? (Are there records of all publicity/media work around the trial in each LGA) Has a non-professional befriending network been set up? How does this function? Who are the befrienders and the recipients? (Is there data collection on numbers of volunteers registering with network and mothers referred for 'befriending'; focus group discussions with volunteers and recipients)

Changes in comparison communities over the intervention period will be assessed by 'unobtrusive monitoring' [47], using document reviews, media analysis, information on policy and practice development and on the provision of resources relevant to maternal health by local, regional, State and Commonwealth departments.

Economic and 'ecological' evaluation

An economic evaluation will be carried out in a parallel but independent study *EcoPRISM*[iii].

Data analysis plan

• Comparison of the numbers of women who had a live-born, surviving infant during the study period in intervention and comparison communities; data on all births from the Victorian Perinatal Data Collection Unit (VPDCU);

• Comparison of the adjusted response fraction, taking into account questionnaires returned to sender, in each intervention and comparison community;

• Comparison of the social and reproductive characteristics of respondents and non-respondents in each intervention and comparison community, using data on all births in these communities from the VPDCU;

• Analysis by intention to treat, adjusting for clustering:

• Proportion of women with EPDS $\geq$ 13 comparing intervention and comparison communities;

• Comparison of mean(sd) SF-36 scores for each of the SF-36 domains, in intervention and comparison communities;

• Comparison of the mean (sd) SF-36 physical and mental component scores in intervention and comparison communities;

• Odds ratio for probable depression (EPDS $\geq$ 13) comparing intervention and comparison communities, adjusting for any imbalance in major covariates;

• Descriptive statistics for aspects of the process evaluation in intervention communities, e.g. extent of mother-and-baby friendly environments;

• Descriptive statistics with respect to opportunities to meet new people and to make new friends, to have 'time-out', having 'someone to talk to', being able to talk to own GP, being able to talk to own MCHN, being able to talk to partner.

Assessment of the effect of the intervention with respect to depression will be made adjusting for the correlated structure of the outcome data [43]. Trends in the proportions of women depressed over the 12 months will also be assessed because, if the intervention is effective, its effectiveness is likely to be cumulative, so that an appropriate analysis would compare changes over time in the intervention and comparison communities. Analysis of the SF-

36 will also take into account clustering and change over time. It is likely that the analysis will require hierarchical modelling. We recognise that there is continuing development and debate about the appropriate methods of analysis for cluster-randomised trials and the research team will continue to monitor these developments for the purposes of refining the proposed data analysis.

Ethical issues

The key ethical issues in randomised *clinical* trials are:

• a worthwhile question in terms of the size of the problem addressed and its associated mortality, morbidity, disability or resource implications;

• informed consent to participation, including consent to randomisation, with the additional proviso that consent may be withdrawn at any time, and that the care provided by the agency/institution and its staff will not be influenced by decisions about participation;

• genuine uncertainty about the effectiveness of the intervention;

• an assessment of the intervention as unlikely to be harmful;

• an adequate sample size to identify the pre-specified effects of the intervention;

• prior systematic review of any previous trials or similar interventions.

The application of these principles to *community*-based trials is relatively straightforward, and PRISM satisfies all but one of the above criteria. The issue of informed consent is more complex in community-randomised trials. Formal consent to randomisation and to implementation will be sought from local government authorities (LGAs) on behalf of the population they are responsible for, and the staff they employ, within a Memorandum of Understanding between the LGA and La Trobe University. Women will be sent a postal questionnaire six months and two years after the birth, with a covering letter making it clear that they do not have to return it. If they do complete the questionnaire that is taken as consent. The covering letter will provide names and telephone numbers of the researchers for contact if there are any problems or queries and will also identify the contact person for La Trobe University Human Research Ethics Committee to whom complaints can be made.

Other ethical issues which are relevant to this trial are those relating to confidentiality and privacy, including confidentiality and privacy with respect to individual communities. Information about participants will be stored securely: geographic security of questionnaires, separation of names from data wherever women have given their names, locked filing cabinets, password protected computer access. Only aggregated data will be published and data from individual communities will not be identified in publications.

This project was approved by the Research Ethics Committees of Monash University (1994) and La Trobe University (1995).

Discussion

Changes to the protocol after the final research application in March 1998

More detailed costing suggested that it would be possible to fund the intervention in eight rather than seven communities, and to measure the outcomes in 16 rather than 14 communities. High levels of interest and willingness to participate by rural local government authorities during the recruitment period (March to May 1998) led to a decision to include four rural LGA pairs and four metropolitan LGA pairs, rather than the original plan of two rural pairs and five metropolitan pairs (April to June, 1998).

As a result of piloting the mailing procedures it was decided that two reminder cards rather than one would be sent, at two and four weeks after the mail-out of the questionnaires. These combined a 'thank-you' to everyone who had returned a questionnaire and a reminder to anyone who had not yet done so. This process was foreshadowed in the covering letter which accompanied the questionnaire.

Quality assurance processes were set up when the mail-out of questionnaires to women began to ensure: weekly monitoring of mail-outs in each community; immediate referral of all telephone queries about the outcome questionnaire or the reminder cards, or about *PRISM*, to a member of the *PRISM* research team; systematic documentation of all such calls, including the reason for the call, the response and any further follow-up.

Plans for an interim analysis were cancelled. The time taken for most health outcome questionnaires to be returned was up to six or eight weeks after they were mailed out. There was an additional time requirement of about four weeks for coding them. Thus by the time the first three months of questionnaires were ready for data entry, let alone analysis, the third quarter of the 12 months mail-out was just starting. Thus an interim analysis could not be carried out in time to modify the study plan.

The period of outcome data collection was extended from 12 months to 18 months in response to two findings early in the measurement of health outcomes. The first was differences between the numbers of births in participating LGAs in 1999–2000, and their birth numbers in 1996 when the protocol was developed. The second was a lower adjusted response fraction to the outcome questionnaire than to earlier population-based surveys of recent mothers in Victoria. A additional factor was that the use of the geometric instead of the arithmetic mean in the original power calculation provided a slight under-estimate of the total number of participants required. All 16 LGAs agreed to the request to extend the data collection period (May to August, 2001).

A full data analysis plan was developed in 2002 prior to the completion of cleaning and coding the data:

• Table: comparison of the numbers of women who had a live-born, surviving infant during the study period in intervention and comparison communities; data from the Victorian Perinatal Data Collection Unit (VPDCU) and from the LGA Maternal and Child Health databases. *Note*: both sources have known small errors: the VPDCU in relation to assignment of postcodes to LGAs in some boundary zones; the Maternal and Child Health databases in relation to possible duplicate entry for women who move house within the LGA, and incomplete updating of vital status;

• Estimation of the adjusted response fraction, taking into account questionnaires returned to sender, by intervention and comparison communities, pooled for intervention and comparison;

• Participant flow diagram (CONSORT guidelines [48], and CONSORT guidelines adapted for cluster randomised trials [49]); all analyses by 'intention to treat';

• Table: characteristics of respondents and non-respondents with respect to social and reproductive factors available in the VPDCU, by intervention and comparison communities, pooled for intervention and comparison;

• Figure 1: plot of the proportion of women probably depressed (EPDS >≥ 13), with 95% confidence intervals (CI), for each intervention and each comparison community (not identified); comparison of the mean of the cluster means;

• Table: mean SF-36 summary scores (PCS and MCS), by intervention and comparison communities, pooled for intervention and comparison, adjusted for any imbalance in key covariates, and adjusted for clustering using survey analysis procedures (STATA statistical software package Release 8.0, 2002, Stata Corporation, College Station, USA);

• Table: mean EPDS scores, by intervention and comparison communities, pooled for intervention and comparison, adjusted for any imbalance in key covariates, and adjusted for clustering;

• Odds ratio for probable depression (EPDS ≥ 13), comparing pooled intervention and comparison communities, adjusting for any imbalance in major covariates and adjusting for clustering;

• Table: descriptive statistics for aspects of the process evaluation in intervention communities;

• Table: descriptive statistics for ratings of making new friends, mother-and-baby friendly environments, 'time-out', 'someone to talk to', able to talk to GP, able to talk to MCHN, able to talk to partner, comparing pooled intervention and comparison communities, adjusting for any imbalance in major covariates and adjusting for clustering;

• Pre-specified subgroup analyses of the primary health outcomes, looking for interactions: rural/urban, NESB/ESB, low income/other, no partner/partner, with adjustments as described above, if appropriate.

Process evaluation strategies are summarised in Table 1 [see Additional file 1].

Changes to the planned intervention during implementation

The objective of improving linkages between primary care and community mental health services was not implemented successfully. This was largely because the policy focus in the relevant Division (Community, Aged and Mental Health) of the Victorian Department of Human Services at that time was serious mental illness, and the Department's priority in relation to postnatal depression was the establishment of in-patient mother and baby units in each health region (Meeting held by senior staff of the Division with the research team in August 1998).

The research contribution to developing appropriate 'befriending' activities was not implemented. The plan had been that the social network interventions would be preceded by formative research with mothers, partners and service networks. This plan was guided by theory and evidence highlighting the importance of tailoring interventions to precise social network properties (size, density, reciprocity, intimacy, instrumental, informational and emotional support) [39,50]. We were conscious that certain properties of networks can be a source of stress and

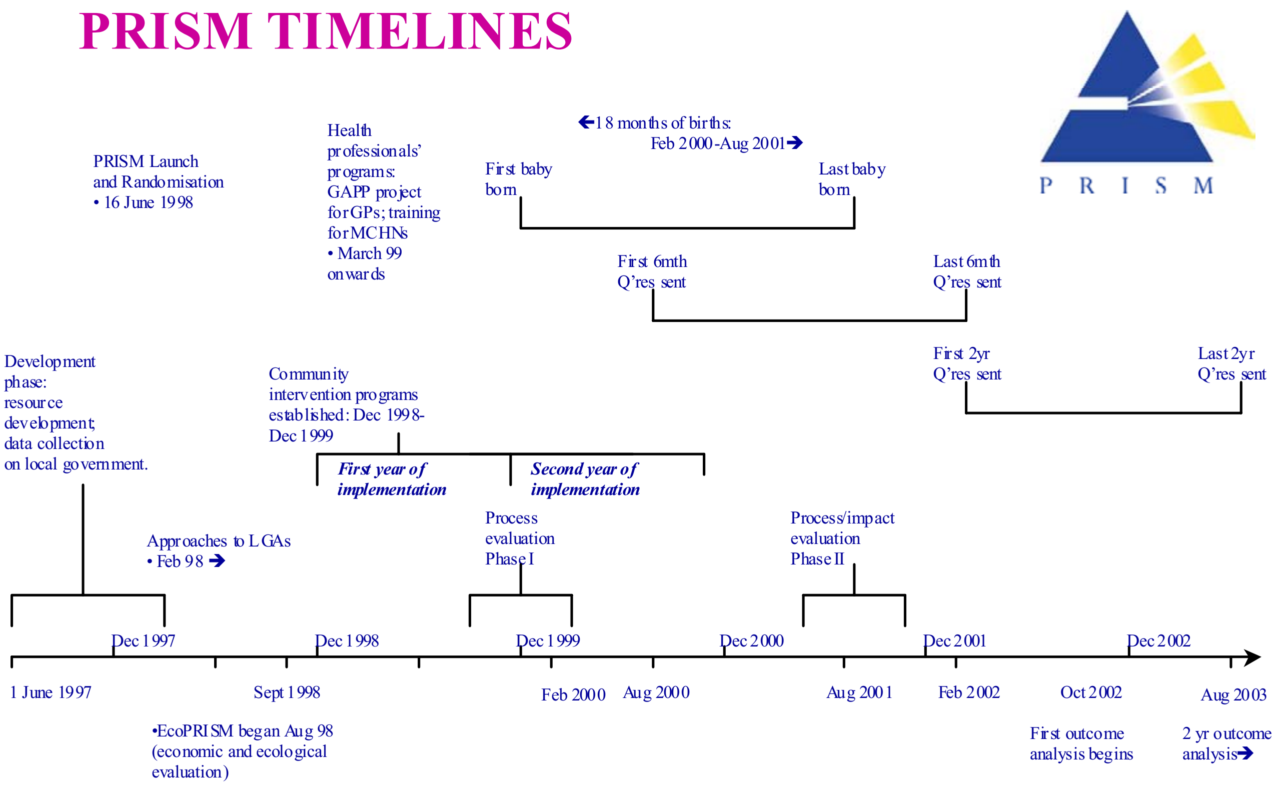

Figure 1
This file shows the planned timelines for planning, implementation and outcome measurement in PRISM at the time of the first successful grant application (1997).

that poorly conceived interventions risk extinguishing the benefits of natural (as opposed to professional) help practices. However, when befriending possibilities were discussed in intervention communities the factors affecting implementation were what key players, especially MCHNs, regarded as locally acceptable, feasible and appropriate.

Process elements relating to the delivery of specific aspects of the intervention were piloted in conjunction with the draft health outcomes questionnaire. There were also two pilots of the mail-out processes which included all 16 communities. This replaced the small separate survey, outlined in the original application. Feedback regarding program implementation and priorities, towards the end of the establishment phase, was sought via a postal survey of all steering committee members and other key stakeholders including councillors, local government staff, Divisions of General Practice and representatives of community organisations.

The follow-up study two years after birth had been planned to include only those women who were depressed at time one (six months after birth) and an equivalent random sample of women not depressed. The complexity of carrying out a second rolling mail-out over 18 months through LGAs, to follow on immediately after the original one, led to a decision to use exactly the same procedures as for the first mail-out, including sending all women who had been sent a questionnaire six months after birth, a second questionnaire when their babies were two years old.

Project website

A project website at http://www.latrobe.edu.au/csmch/prism was launched in June 2003 to make available, as

widely as possible, details about the rationale, aims, implementation and process evaluation of *PRISM* before the primary study outcomes are published. This dissemination strategy is intended to enable those interested in the implementation and evaluation of health and social interventions to read about, and critique, what actually happened in *PRISM*, in advance of the study findings.

List of abbreviations

C Comparison, comparison

CDO Community Development Officer

CI Confidence interval

CONSORT Consolidated Standards of Reporting Trials

EcoPRISM An ecological and economic evaluation of PRISM

EPDS Edinburgh Postnatal Depression Scale

GAPP Guidelines for Assessing Postnatal Problems

GP General Practitioner/General Practice

I Intervention, intervention

LGA Local Government Authority

MCHN Maternal and Child Health Nurse

PRISM Program of Resources, Information and Support for Mothers

SF-36 Short Form 36, Health status measure

VPDCU Victorian Perinatal Data Collection Unit

Competing interests

None declared.

Authors' contributions

JL, RS, LW and SB conceived the trial design and the intervention and wrote the first successful grant application. RS and SB were jointly responsible for the co-ordination and implementation of *PRISM*. RS and SB developed the training program for maternal and child health nurses, with input from JG. JG, who developed the training program for GPs within a separate but linked project, wrote the *Guidelines for Assessing Postnatal Problems* with input from JL, SB and RS. JL, RS, SB, CM, JG and LW were involved in the development of the questionnaire sent to women six months after birth. WD (to 1999), together with RS and SB, developed processes for mapping communities and seeking their participation. CM (from 1999), developed the data collection and monitoring systems with participating communities, and was responsible for data management, coding and cleaning. LW provided statistical expertise, oversight of the randomisation, and carried out the analysis with input from CM. All the authors were members of the research team, which met regularly through the project and all have commented on drafts of this protocol. JL was responsible for the overall direction of the project and wrote the protocol, using the original documents and records.

Notes

i Gunn J, Lumley J, Brown S, Small R, Robertson K. Guidelines for Assessing Postnatal Problems (GAPP), funded by a General Practice Evaluation Program Project Grant, Department of Health and Aged Care, 1999–2001.

ii One is the City of Melbourne, including the central business district of the capital city, which cannot be paired with any other Victorian LGA. The other is the City of Wodonga which has substantial crossover in service and business links with the adjacent City of Albury in New South Wales.

iii Shiell A, Hawe P, Lumley J, Watson L. *EcoPRISM*: an economic and ecological evaluation of *PRISM*, was funded by the National Health and Medical Research Council, 1998–2002. The research team comprised Shiell A, Hawe P, Riley T (University of Sydney, subsequently University of Calgary), Gold L (La Trobe University).

Additional material

Additional File 1
This file is a cross-tabulation of the six major strategies implemented in the intervention by the sources of information which will allow an assessment of the extent to which the intervention strategies met their intended goals in terms of implementation, reach, quality, participants' views of the strategy, and its impact.
Click here for file
[http://www.biomedcentral.com/content/supplementary/1471-2458-3-36-S1.pdf]

Acknowledgements

Funding: Research

The Victorian Health Promotion Foundation (VicHealth) provided core funding of the Centre for the Study of Mothers' and Children's Health through a Program Grant, from 1991–2003. The General Practice Evaluation Program funded the research project *Guidelines for Assessing Postnatal Problems (GAPP)*. The National Health and Medical Research Council funded the research component of *PRISM* and *EcoPRISM*. The NHMRC also awarded Stephanie Brown a Public Health Travelling Fellowship to facilitate liaison with researchers in the UK on postnatal care and the design of clus-

ter trials. Additional research funding for *PRISM* came from the Felton Bequest and the Department of Human Services.

Funding: Implementation

PRISM implementation in local communities was funded by VicHealth and the Victorian Department of Human Services, (Youth and Community Services, Public Health, Acute Health, Mental Health Divisions) following seed funding with an Industry Collaborative Grant from La Trobe University and the Department of Human Services. The Sidney Myer Fund, and the Felton Bequest also contributed to implementation. Participating local governments made in kind contributions and other significant support.

References

1. Brown S, Lumley J: **Maternal health after childbirth: results of an Australian population-based survey.** *Br J Obstet Gynaecol* 1998, **105:**156-161.
2. Astbury J, Brown S, Lumley J, Small R: **Birth events, birth experiences and social differences in postnatal depression.** *Aust J Public Health* 1994, **18:**176-184.
3. Cox JL, Holden JM, Sagovsky R: **Detection of postnatal depression: development of the 10-item Edinburgh Postnatal Depression Scale.** *Br J Psychiatry* 1987, **150:**782-786.
4. Harris B, Huckle P, Thomas R, John S, Fung H: **The use of rating scales to identify postnatal depression.** *Br J Psychiatry* 1989, **154:**813-817.
5. Murray L, Carothers AD: **The validation of the Edinburgh Postnatal Depression Scale on a community sample.** *Br J Psychiatry* 1990, **157:**288-290.
6. Boyce P, Stubbs R, Todd A: **The Edinburgh Postnatal Depression Scale: validation in an Australian sample.** *Aust NZ J Psychiatry* 1993, **27:**472-6.
7. Hickey AR, Boyce PM, Ellwood D, Morris-Yates AD: **Early discharge and risk for postnatal depression.** *Med J Aust* 1997, **167:**244-247.
8. Cogill SR, Caplan HL, Alexandra H, Robson KM, Kumar R: **Impact of maternal postnatal depression on cognitive development of young children.** *Br Med J* 1986, **292:**1165-1176.
9. Cutrona CE, Troutman BR: **Social support, infant temperament, and parenting self-efficacy: a mediational model of postpartum depression.** *Child Dev* 1986, **57:**1507-1518.
10. Murray L, Fiori-Cowley A, Hooper R, Cooper P: **The impact of postnatal depression and associated adversity on early mother-infant interactions and later infant outcomes.** *Child Dev* 1996, **67:**2512-2526.
11. Small R, Brown S, Lumley J, Astbury J: **Missing voices: what women say and do about depression after childbirth.** *J Reprod Inf Psychol* 1994, **12:**89-103.
12. Small R, Astbury J, Brown S, Lumley J: **Depression after childbirth. Does social context matter?** *Med J Aust* 1994, **161:**473-477.
13. Romito P: **Unhappiness after childbirth.** In: *Effective care in pregnancy and childbirth* Edited by: *Chalmers I, Enkin M, Keirse M. Oxford: Oxford University Press*; 1989:1433-1466.
14. MacArthur C, Knox EG: **Health after childbirth: an investigation of long term health problems beginning after childbirth in 11,701 women.** *London : HMSO* 1991.
15. Glazener C, Abdalla M, Stroud P, Naji S, Templeton A, Russell I: **Postnatal maternal morbidity: extent, causes, prevention and treatment.** *Br J Obstet Gynaecol* 1995, **102:**282-287.
16. Gjerdingen D, Froberg OG, Chaloner KM, McGovern P: **Changes in women's physical health during the first postpartum year.** *Arch Fam Med* 1993, **2:**277-283.
17. Brown S, Lumley J: **Physical health problems after childbirth and maternal depression at six to seven months postpartum.** *Br J Obstet Gynaecol* 2000, **107:**1194-1201.
18. Glazener CM: **Sexual function after childbirth; women's experiences, persistent morbidity and lack of professional recognition.** *Br J Obstet Gynaecol* 1997, **104:**330-5.
19. MacArthur C, Bick DE, Keighley MRB: **Faecal incontinence after childbirth.** *Br J Obstet Gynaecol* 1997, **104:**46-50.
20. Small R, Lumley J, Yelland J: **How useful is the concept of somatisation in cross-cultural studies of maternal depression? A contribution from the Mothers in a New Country project.** *J Psychosom Obstet Gynecol* 2003, **24:**45-52.
21. Small R, Lumley J, Yelland J: **Cross-cultural experiences of maternal depression: associations and contributing factors for Vietnamese, Turkish and Filipino immigrant women in Victoria, Australia.** *Ethnicity & Health* 2003, **8:**189-206.
22. Department of Human Services, Victoria: **Maternal and Child Health Program. Annual Report 1995–96. Melbourne.** *Department of Human Services* 1997.
23. Department of Human Services, Victoria: **Maternal and Child Health Consumer Survey. Project Report 1995–96.** 1997.
24. Gunn J, Lumley J, Young D: **Visits to general practitioners in the first six months of life.** *J Paediatr Child Health* 1996, **32:**310-315.
25. Gunn J, Lumley J, Young D: **The role of the general practitioner in postnatal care: a survey from Australian general practice.** *Br J Gen Pract* 1998, **48:**1570-74.
26. Stamp G, Williams AS, Crowther C: **Evaluation of antenatal and postnatal support to overcome postnatal depression. A randomised controlled trial.** *Birth* 1995, **22:**138-143.
27. Leverton TJ, Elliott SA: **Transition to parenthood groups: a preventive intervention for postnatal depression?** In: *The Free Woman: Women's Health in the 1990s* Edited by: *Van Hal EV, Everaerd W. Carnforth: The Parthenon Publishing Group*; 1989:479-486.
28. Gordon RE, Gordon KK: **Social factors in the prevention of postpartum emotional problems.** *Obstet Gynecol* 1960, **15:**433-437.
29. Fleming AS, Klein E, Corter C: **The effects of a social support group on depression, maternal attitudes and behaviour in new mothers.** *J Child Psychol Psychiatry* 1992, **33:**685-698.
30. Wickberg B, Hwang CP: **Counselling of postnatal depression: A controlled study on a population based Swedish sample.** *J Affect Disorders* 1996, **39:**209-16.
31. Holden JM, Sagovsky R, Cox JL: **Counselling in a general practice setting: controlled study of health visitor intervention in treatment of postnatal depression.** *Br Med J* 1989, **298:**223-26.
32. Gerrard JK, Holden JM, Elliott SA, McKenzie P, McKenzie J, Cox JL: **A trainer's perspective of an innovative programme teaching health visitors about the detection, treatment and prevention of postnatal depression.** *J Adv Nursing* 1993, **18:**1825-32.
33. Regier D, Hirschfeld RM, Goodwin FKJ, Burke JD, Lazar JB, Judd LL: **The NIMH depression awareness, recognition and treatment program.** *Am J Psychiatry* 1988, **145:**1351-57.
34. Gask L: **Teaching psychiatric interviewing skills to general practitioners.** In: *The prevention of depression and anxiety the role of the primary care team* Edited by: *R Jenkins, J Newton, R Young. London: HMSO*; 1992:39-45.
35. Davis DA, Thomson MA, Oxman AD, Haynes RB: **Changing physician performance.** *JAMA* 1995, **274:**700-705.
36. Johnson Z, Howell F, Molloy B: **Community mothers program: a randomised trial of a non professional intervention.** *BMJ* 1993, **306:**1449-1452.
37. Cox AD, Pound A, Mills M, Puckering C, Owen AL: **Evaluation of a home visiting and befriending scheme for young mothers: Newpin.** *J Roy Soc Med* 1991, **84:**217-220.
38. **Lay health advisors: a critical link to community capacity building.** *Health Educ Behavior (Special issue)* 1997, **24:**413-522.
39. Israel BA: **Social networks and social support: implications for natural helpers and community-level interventions.** *Health Educ Q* 1985, **21:**65-80.
40. Henderson C, ed: **Women's health after birth: what's happening in postnatal care?** *[Supplement] Br J Midwifery* 1997, **5:**608-622.
41. Stokols D: **Translating social ecological theory into guidelines for community health promotion.** *Am J Health Prom* 1996, **10:**282-298.
42. Garratt AM, Ruta DA, Abdalla MI, Buckingham JK, Russell IT: **The SF36 health survey questionnaire: an outcome measure suitable for routine use within the NHS?** *BMJ* 1993, **306:**1440-4.
43. Shipley MJ, Smith PG, Dramaix M: **Calculation of power for matched pair studies when randomization is by group.** *Int J Epidemiol* 1989, **18:**457-461.
44. McKinlay SM: **Cost-efficient designs of cluster unit trials.** *Prev Med* 1994, **23:**606-611.
45. Brown S, Lumley J: **Antenatal care: a case of the inverse care law?** *Aust NZ J Public Health* 1993, **17:**95-103.

46. Brown S, Lumley J: **The 1993 Survey of Recent Mothers: issues in survey design, analysis and influencing policy.** *Int J Qual Health Care* 1997, **9:**265-275.
47. Kellehear A: **The unobtrusive researcher. A guide to methods.** *Sydney, Allen and Unwin* 1993.
48. Moher D, Schulz KF, Altman DG: **The CONSORT statement: revised recommendations for improving the quality of reports of parallel group randomised trials.** *BMC Medical Research Methodology* 2001, **1:**2.
49. Elbourne DE, Campbell MK: **Extending the CONSORT statement to cluster randomized trials: for discussion.** *Stat Med* 2001, **20:**489-496.
50. Barrera M: **Social networks and the third law of ecology.** *Am J Comm Psychol* 1991, **19:**133-138.

Pre-publication history

The pre-publication history for this paper can be accessed here:

http://www.biomedcentral.com/1471-2458/3/36/prepub

Publish with **BioMed Central** and every scientist can read your work free of charge

"BioMed Central will be the most significant development for disseminating the results of biomedical research in our lifetime."

Sir Paul Nurse, Cancer Research UK

Your research papers will be:

- available free of charge to the entire biomedical community
- peer reviewed and published immediately upon acceptance
- cited in PubMed and archived on PubMed Central
- yours — you keep the copyright

Submit your manuscript here:
http://www.biomedcentral.com/info/publishing_adv.asp

BioMedcentral
